# General low-temperature reaction pathway from precursors to monomers before nucleation of compound semiconductor nanocrystals

**DOI:** 10.1038/ncomms12223

**Published:** 2016-08-17

**Authors:** Kui Yu, Xiangyang Liu, Ting Qi, Huaqing Yang, Dennis M. Whitfield, Queena Y. Chen, Erik J. C. Huisman, Changwei Hu

**Affiliations:** 1 Key Laboratory of Green Chemistry and Technology, Ministry of Education, College of Chemistry, Sichuan University, Chengdu 610065, China; 2 Security and Disruptive Technologies, National Research Council Canada, Ottawa, Ontario, Canada K1A 0R6; 3 College of Chemical Engineering, Sichuan University, Chengdu 610065, China

## Abstract

Little is known about the molecular pathway to monomers of semiconductor nanocrystals. Here we report a general reaction pathway, which is based on hydrogen-mediated ligand loss for the precursor conversion to ‘monomers' at low temperature before nucleation. We apply ^31^P nuclear magnetic resonance spectroscopy to monitor the key phosphorous-containing products that evolve from *MX*_*n*_+*E*=PPh_2_H+H*Y* mixtures, where *MX*_*n*_, *E*=PPh_2_H, and H*Y* are metal precursors, chalcogenide precursors, and additives, respectively. Surprisingly, the phosphorous-containing products detected can be categorized into two groups, Ph_2_P–*Y* and Ph_2_P(*E*)–*Y*. On the basis of our experimental and theoretical results, we propose two competing pathways to the formation of *M*_2_*E*_*n*_ monomers, each of which is accompanied by one of the two products. Our study unravels the pathway of precursor evolution into *M*_2_*E*_*n*_ monomers, the stoichiometry of which directly correlates with the atomic composition of the final compound nanocrystals.

## Abstract (web summary)

Little is known about the molecular pathway to monomers of semiconductor nanocrystals. Here, the authors report a general reaction pathway based on hydrogen-mediated ligand loss for the precursor conversion to ‘monomers' at low temperature prior to nucleation, via ^31^P NMR spectroscopic measurements.

## Graphical abstract

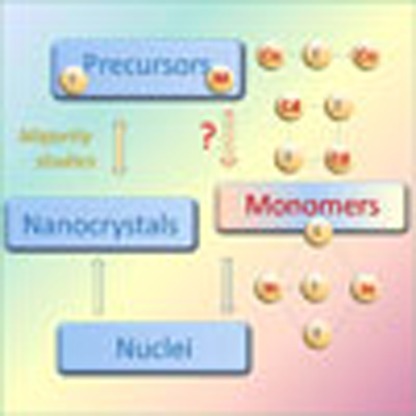

Colloidal semiconductor nanocrystal (NC) quantum dots (QDs) with distinct properties and well-acknowledged potential in many applications such as light-emitting diodes^1^,^2^, lasing^3^,^4^, photovoltaics^5^,^6^ and bio-labelling/imaging^7^,^8^,^9^ have been the focus of intense research ranging from fundamental science to applied technologies. For the past two decades, there have been significant efforts made to advance NC syntheses^10^,^11^,^12^,^13^,^14^,^15^,^16^,^17^,^18^,^19^,^20^,^21^,^22^,^23^,^24^,^25^,^26^,^27^,^28^,^29^,^30^,^31^,^32^,^33^,^34^,^35^,^36^,^37^,^38^. Mainly, the wet-chemical synthesis of colloidal metal chalcogenide NCs depends on the use of metal salts such as cadmium oleate (Cd(OA)_2_ or Cd(OOCC_17_H_33_)_2_) and tri-*n*-octylphosphine chalcogenides (*E*=P(C_8_H_17_)_3_ or *E*TOP, *E*=S, Se, and Te), together with beneficial additives in 1-octadecene (ODE) including diphenylphosphines^13^,^14^,^15^,^16^,^17^,^18^,^19^, primary alkyl amines^16^,^25^,^26^,^27^,^28^,^29^,^30^,^35^,^36^, thiols^22^,^29^,^30^,^31^ and alcohols^14^,^27^,^34^.

A major advance in the NC synthesis occurred with the recognition that commercial tertiary phosphine TOP contains dioctylphosphine (HP(C_8_H_17_)_2_, a secondary phosphine) that acts as an active impurity facilitating NC nucleation/growth but leading to low synthetic reproducibility (because of its varying amount from batch to batch)^13^,^14^,^15^,^23^. It was first suggested^15^ and then experimentally demonstrated^23^ that the use of commercial diphenylphosphine (HP(C_6_H_5_)_2_ or HPPh_2_, a secondary phosphine) resulted in an equilibrium of SeTOP+HPPh_2_⇌TOP+Se=PPh_2_H. Meanwhile, high metal-to-Se and low Se-to-TOP feed ratios were found to shift the equilibrium to the right^23^, which remarkably improved the NC synthesis with high particle yield and synthetic reproducibility at low reaction temperatures^17^,^18^,^19^,^21^,^22^,^38^. *E*=PPh_2_H is much more reactive than *E*TOP^15^,^23^. The use of *E*TOP+HPPh_2_ leading to the *E* precursor of *E*=PPh_2_H instead of *E*TOP has been shown to be beneficial for the synthesis of NCs such as PbSe (refs 17, 18) and CdSeS (ref. 38), while the direct use of *E*=PPh_2_H (made from *E*+HPPh_2_) is preferable to the synthesis of NCs such as ZnSe (ref. 19), ZnSeS (ref. 21) and CuInS_2_ (ref. 22). With the large number of recipes developed, recent studies have demonstrated clearly that the control of precursor reactivity has a strong impact on the reproducibility, particle yield, and size and size distribution of the resulting NCs^17^,^18^,^19^,^21^,^22^,^36^,^37^,^38^. For example, the reactivity of thiourea precursors was shown to control the size, yield and batch-to-batch consistency of PbS NCs^37^.

Generally, the current state-of-the-art in NC synthesis is principally empirical, with little insight into the stepwise pathway by which monomers are generated. There exists ‘an induction period' before nucleation occurs, which was briefly addressed for CdSe from Cd*X*_2_+SeP*R*_3_ (*X*=carboxylate and *R*=alkane groups ref. 33). During the induction period, the consumption of SeP*R*_3_ was visible, but NC absorbance did not appear. The consumption of SeP*R*_3_ was claimed to accumulate ‘solutes' that may be composed of multiple monomer units; afterwards, nucleation took place. Accordingly, the formation of ‘monomers' from precursors takes place at the beginning of the ‘induction period'.

With the monomer in the form of Cd_1_Se_1_ instead of Cd_2_Se_2_, the pathway from precursors to Cd_1_Se_1_ monomers was recently documented for CdSe NCs in the presence of C_18_H_35_NH_2_ as one additive from the reaction of Cd(OA)_2_+SeTOP+HPPh_2_. The P-containing products Ph_2_P-OOCC_17_H_33_ (**1a**), Ph_2_P−PPh_2_ (**1b**), Ph_2_P-NHC_18_H_35_ (**1c**) and Ph_2_P(Se)–NHC_18_H_35_ (**2c**) were detected, and the equilibrium of **1c**+Se=PPh_2_H⇌**2c**+HPPh_2_ was demonstrated^25^. Different NC systems are supposed to follow different reaction pathways to their monomers; for this seemingly obvious reason, we decided to investigate individual pathways from precursor evolution to monomers at low reaction temperatures in each of the reactions of *MX*_*n*_+*nE*=PPh_2_H+H*Y*, where *M*=Cu (I), Cd (II), Zn (II), Ge (II), Pb (II) and In (III), *E*=S, Se and Te, and H*Y*=*R*COOH, HPPh_2_, *R*NH_2_, *R*SH and *R*OH. The anion *X* for the starting metal cations was chosen such that *MX*_*n*_ is soluble under the reaction conditions and can often be a long-chain alkyl carboxylate or thiolate. This reaction system has proven to be practical for the synthesis of various NCs with high quality, enhanced reproducibility and yield^17^,^18^,^19^,^21^,^22^,^38^. However, the pathway from precursors to monomers of the reaction *MX*_*n*_+*nE*=PPh_2_H+H*Y* is perplexing to study because of the inevitable presence of H*X* and HPPh_2_ (as explained by the below equations 1, 2, 3, 4, 5, 6).

Here, we present our study on the reaction pathway from precursors to *M*_2_*E*_*n*_ monomers for the reaction of *MX*_*n*_+*nE*=PPh_2_H+H*Y*. Conclusively, various P-containing compounds are detected for the metal *M*=Cu (I), Cd (II), Zn (II), Ge (II), Pb (II) and In (III) in combination with the chalcogen *E*=S, Se and Te. Most importantly and surprisingly, we are able to categorize these P-containing compounds into two groups of Ph_2_P–*Y* (**1**) and Ph_2_P(*E*)–*Y* (**2**), which are summarized in Supplementary Table 1 and Supplementary Note 1 (with *Y*=–OOCC_17_H_33_ (**a**), –PPh_2_ (**b**), –NHC_18_H_35_ (**c**), –SC_12_H_25_ (**d**) and –OC_12_H_25_ (**e**)), together with the detailed information on their assignment that includes our calculation of ^31^P NMR chemical shifts and a summary of the experimental information available in the literature. Accordingly, we propose two competing pathways leading to *M*_2_*E*_*n*_ monomers as illustrated by equations 1 and 2 (with further explanation in equations 3, 4, 5, 6).


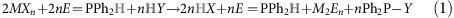



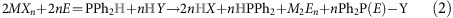


where *M*=cation Cu (I), Cd(II), Zn (II), Ge (II), Pb (II) and In (III), *n*=1, 2 and 3 of the oxidation state of monovalent, divalent and trivalent *M*, respectively, *X*=anion (such as carboxylate C_17_H_33_COO^−^), *E*=S, Se and Te, and additive H*Y*=*R*COOH (**a**), HPPh_2_ (**b**), *R*NH_2_ (**c**), *R*SH (**d**) and *R*OH (**e**). A monomer in the form of *M*_2_*E*_*n*_ is proposed, which leads to NC nucleation followed by growth. The H atoms involved in the first and second H-mediated ligand loss steps are denoted as **H**′ and **H**″, respectively. It is noteworthy that other secondary phosphines such as dicyclohexylphosphine (HPCy_2_) leads to precursor *E*=PCy_2_H to start with; the correlation between the reactivity of *E*=P*R*_2_H (such as with *R*=Ph or Cy) and the size of the resulting NCs will be the subject of another study. Our general stepwise pathway from precursor evolution to *M*_2_*E*_*n*_ monomers at low reaction temperatures should result in a much more in-depth fundamental understanding, which may advance the design and synthesis of colloidal semiconductor NCs and advance the realization of their potential.

## Results

### NMR study of various reaction systems

**Figure 1:**
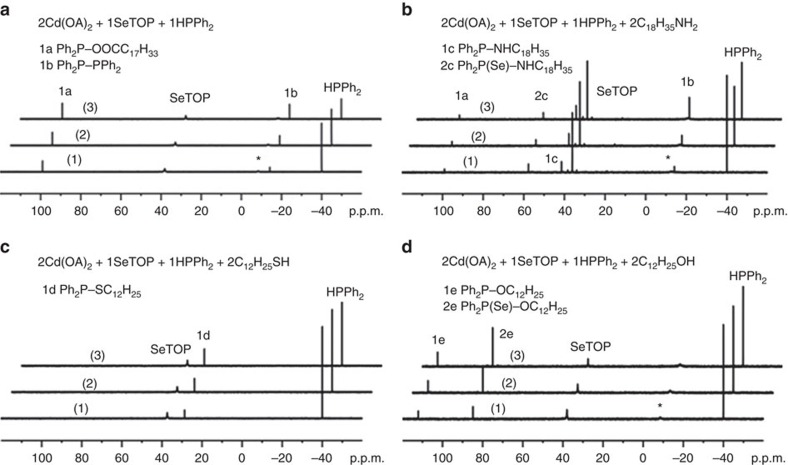
^31^P NMR spectra of CdSe reaction mixtures with Se precursor. (**a**) 2Cd(OOCC_17_H_33_)_2_+SeTOP+HPPh_2_. (**b**) 2Cd(OOCC_17_H_33_)_2_+SeTOP+HPPh_2_+2C_18_H_35_NH_2_. (**c**) 2Cd(OOCC_17_H_33_)_2_+SeTOP+HPPh_2_+2C_12_H_25_SH. (**d**) 2Cd(OOCC_17_H_33_)_2_+SeTOP+HPPh_2_+2C_12_H_25_OH. The experiments were performed at RT for 15 (1), 30 (2) and 60 min (3). The peak denoted with an asterisk (*) is TOP complexed to Cd (ref. 23). Interestingly, the P-containing products detected from the four reactions can be grouped into two product types, Ph_2_P–*Y* (**1**) and Ph_2_P(Se)–*Y* (**2**). The slowest disappearance of SeTOP is with the use of amine, the trend of which is in agreement with our kinetics study (Supplementary Figs 28–31 and Supplementary Note 5) based on our putative pathway proposed (Fig. 6) and the consideration of constitutional isomers (Fig. 7).

**Figure 2:**
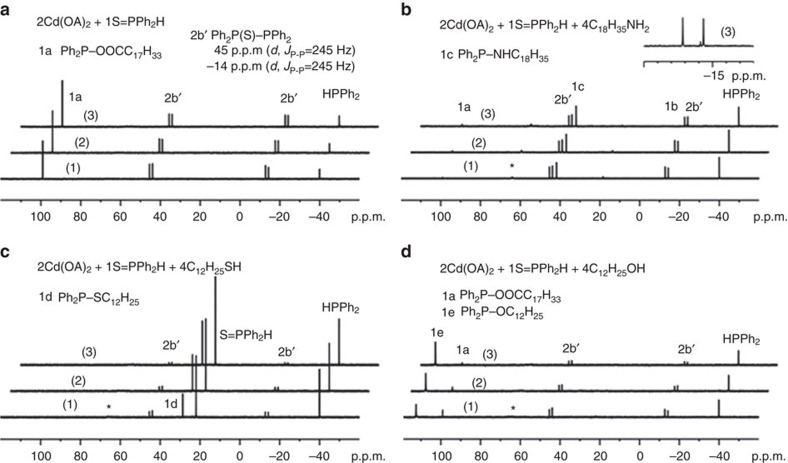
^31^P NMR spectra of CdS reaction mixtures with S precursor. (**a**) 2Cd(OOCC_17_H_33_)_2_+S=PPh_2_H. (**b**) 2Cd(OOCC_17_H_33_)_2_+S=PPh_2_H+4C_18_H_35_NH_2_. (**c**) 2Cd(OOCC_17_H_33_)_2_+S=PPh_2_H+4C_12_H_25_SH. The **1d**−to−S=PPh_2_H ratio increased from 0.28 (1), 0.36 (2) to 0.41 (3) is shown in Supplementary Fig. 2c. (**d**) 2Cd(OOCC_17_H_33_)_2_+S=PPh_2_H+4C_12_H_25_OH. The experiments were carried out at RT for 15 (1), 30 (2) and 60 min (3). The peak denoted with an asterisk (*) is probably Cd(S_2_PPh_2_)_2_ (ref. 36).

**Figure 3:**
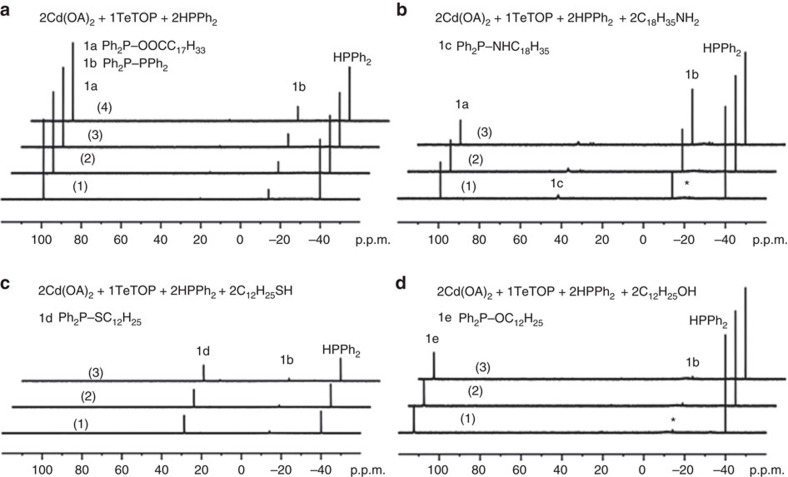
^31^P NMR spectra of CdTe reaction mixtures with Te precursor. (**a**) 2Cd(OOCC_17_H_33_)_2_+TeTOP+HPPh_2_. (**b**) 2Cd(OOCC_17_H_33_)_2_+TeTOP+HPPh_2_+2C_18_H_35_NH_2_. (**c**) 2Cd(OOCC_17_H_33_)_2_+TeTOP+HPPh_2_+2C_12_H_25_SH. (**d**) 2Cd(OOCC_17_H_33_)_2_+TeTOP+HPPh_2_+2C_12_H_25_OH. The experiments were performed at RT for 15 (1), 30 (2), 45 (3) and 60 min (4) for **a**, and 15 (1), 30 (2) and 60 min (3) for the rest. The peak denoted with an asterisk (*) is TOP complexed to Cd (ref. 23).

**Figure 4:**
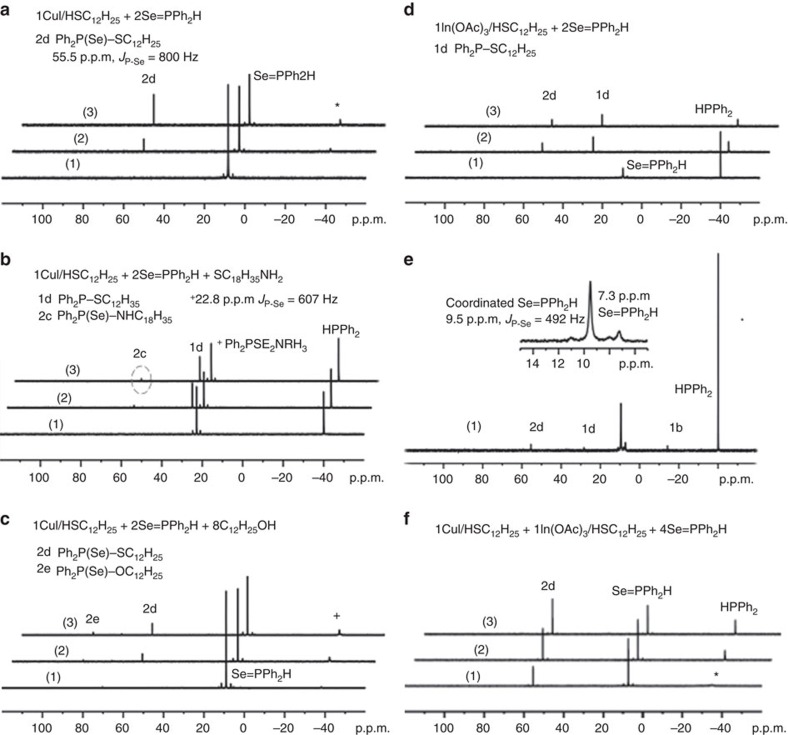
^31^P NMR spectra of Cu/In-containing reaction mixtures. (**a**) CuI/C_12_H_25_SH+2Se=PPh_2_H, with RT (1), 80 °C per 10 min (2) and 100 °C per 10 min (3). (**b**) CuI/C_12_H_25_SH+2Se=PPh_2_H+8C_18_H_35_NH_2_, with RT per 10 min (1), 100 °C per 15 min (2) and 100 °C per 75 min (3). (**c**) CuI/C_12_H_25_SH+2Se=PPh_2_H+8C_12_H_25_OH, with RT (1), 80 °C per 10 min (2), 100 °C per 10 min (3). (**d**,**e**) In(OAc)_3_/C_12_H_25_SH+2Se=PPh_2_H, with RT per 15 min (1), 80 °C per 10 min (2), 100 °C per 10 min (3) for **d** and RT per 15 min (1) for **e** (repeated with 512 scans). (**f**) CuI/C_12_H_25_SH+In(OAc)_3_/C_12_H_25_SH+4Se=PPh_2_H, with RT per 15 min (1), 80 °C per 5 min (2) and 100 °C per 5 min (3). The peak denoted with an asterisk (*) was assigned to HPPh_2_ complexed to Cu (ref. 22).

**Figure 5:**
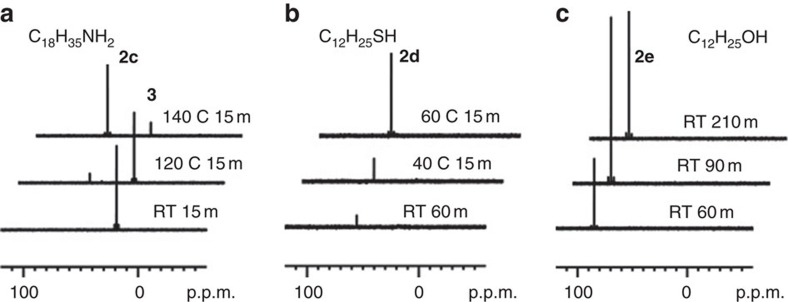
^31^P NMR spectra of reaction mixtures of 3+6Cd(OA)_2_+HY. (**a**) 16RNH_2_. (**b**) 4RSH. (**c**) 16ROH. The slowest disappearance of **3** occurs with the use of amine, the trend of which is in agreement with that shown in Fig. 1. The relevant pathway to the formation of monomers and **2** is shown in Supplementary Fig. 25.

**Figure 6:**
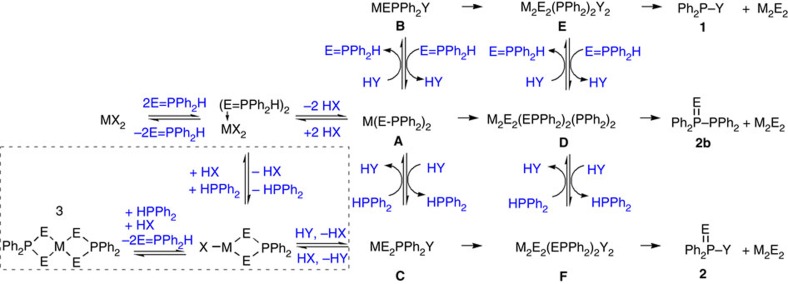
Schematic of the general pathway for formation of *M*_2_*E*_*n*_ monomers. For simplicity, this figure is drawn for *M* (II) only at low temperature from metal carboxylate (*MX*_2_) and dialkylphosphine chalcogenide (*E*=PPh_2_H) with the use of additive H*Y*. Specific schemes for the other metal valences can be constructed accordingly. *E*=S, Se or Te. When H*Y*=*R*COOH (**a**), HPPh_2_ (**b**), *R*NH_2_ (**c**), *R*SH (**d**) and *R*OH (**e**), Compounds **1** and **2** are labelled as **1a**–**e** and **2a**–**e**, respectively. The equilibrium of **1**+*E*=PPh_2_H⇌**2**+HPPh_2_ is worthy of notice (Supplementary Table 2 and Supplementary Note 2). Note that another secondary phosphine, dicyclohexylphosphine (HPCy_2_), was also tested (as shown in Supplementary Figs 1 and 3); precursor *E*=PCy_2_H instead of *E*=PPh_2_H also leads to Compound Cy_2_P–*Y* (**1**) and Cy_2_P(*E*)–*Y* (**2**). The correlation between the reactivity of *E*=P*R*_2_H (with *R*=Ph or Cy) and the size of resulting NCs is the subject of another study. The dotted box is for a system to start from single-source precursors (such as **3** shown in Supplementary Fig. 25 with *E*=Se and *M*=Cd (II)). See Supplementary Fig. 26 and Supplementary Note 3 for the difference of the putative mechanisms proposed by ref. 15 and by the present study. With *Y*=PPh_2_, intermediate **D** in nature is **F**. Here two competing pathways are proposed for the formation of *M*_2_*E*_*n*_ monomer: one is **A**–(**B** or **D)**–**E**–**1**+*M*_2_*E*_*n*_, the other is **A**−(**C** or **D**)–**F**–**2**+*M*_2_*E*_*n*_.

**Figure 7:**
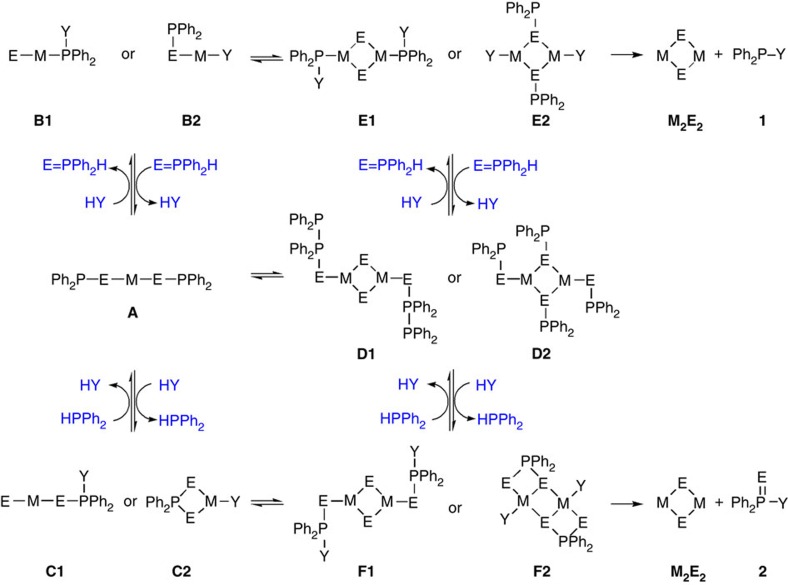
Possible isomers with binding skeletons. Here two of the most probable constitutional isomers of intermediates **B** to **F** are shown; there are 99 CdSe-containing isomer structures computed as shown Supplementary Tables 4–17. With *M*=Cd and *E*=Se, **B1** and **C1** are favoured for *Y*=NHR, whereas **B2** and **C2** are preferred for *Y*=SR, OOCR. For *Y*=OR, the distinction is not clear cut. For the immediate precursors **E** and **F** leading to monomers Cd_2_Se_2_ with Compounds **1** and **2**, respectively, **E1** and **F1** are favoured for *Y*=NHR and OR, whereas **E2** and **F2** are preferred for *Y*=SR and OOCR. For *Y*=PPh_2_, **D2** is more stable than **D1**. Possibly, the release of Compound **1** is more apparent from **E1** (via the *M*–P bond cleavage) than from **E2**, while Compound **2** from **F1** (via the *M*–*E* bond cleavage) than from **F2**.

The reactions studied are presented in Fig. 1 for CdSe and Supplementary Figs 1–9 for *M* (II) Se, Fig. 2 for CdS and Supplementary Figs 10–13 for CdS and ZnS, and Fig. 3 for CdTe and Supplementary Figs 14–21 for GeTe. Figure 4 is for Cu_2_Se, In_2_Se_3_ and CuInSe_2_, and Supplementary Figs 22–23 are for Cu_2_S, In_2_S_3_ and CuInS_2_. Figure 5 deals with [Cd(Se_2_PPh_2_)]_2_ (**3**)+Cd(OA)_2_+H*Y* for the demonstration of equation 2, which leads to Cd_2_Se_2_+**2** as shown by Supplementary Fig. 25 and which is supported by Supplementary Fig. 24 with the reaction of [Cd(Se_2_PPh_2_)]_2_ (**3**)+Cd(OA)_2_+H*Y*+HPPh_2_. On the basis of our *in situ*^31^P NMR monitoring of a large number of reactions dealing with six metal cations (*M*) and three chalcogens (*E*) in the presence of the five types of H*Y* additives, we propose a conceptual pathway (Fig. 6) that demonstrates the probable reactions from precursors to monomers. This distinct pathway starts with the coordination of *n E*=PPh_2_H molecules per *MX*_*n*_, followed by the H-mediated ligand loss of *n* H*X* molecules to result in one *M*(*E*PPh_2_)_*n*_ (**A**). Afterwards, **A** undergoes dimerization to **D** that reacts with H*Y* to **E** and/or **F**, or reacts with H*Y* leading to **B** and/or **C** that undergoes dimerization to **E** and/or **F**, respectively. *M*_2_*E*_*n*_ and **1** are then produced from **E** (equation 1), while *M*_2_*E*_*n*_ and **2** from **F** (equation 2). Metathesis equilibria are involved, in which there are reversible exchanges of small ligand molecules, HPPh_2_, *E*=HPPh_2_ and H*Y* (around metal chalcogenide centres, such as **D**+H*Y*⇌**E**+*E*=PPh_2_H and **D**+H*Y*⇌**F**+HPPh_2_), and chalcogenide exchange reactions such as **1**+Se=PPh_2_H⇌**2**+HPPh_2_, which affect the detection of **1**, **2** and HPPh_2_. The chalcogenide exchange equilibria were examined by density functional theory (DFT) shown in Supplementary Table 2 and Supplementary Note 2. Furthermore, we performed extensive DFT calculations for the probable isomers of the intermediates **A**–**F** shown in Fig. 6; therefore, we are able to elucidate further the pathway we proposed in Fig. 7, in which the probable isomers with detailed bonding skeletons of each intermediate **A**–**F** are illustrated, providing a much deeper understanding.

Figure 1 presents our ^31^P NMR spectra collected from four representative mixtures of Cd(OA)_2_+SeTOP+HPPh_2_ (**a**) and with the additional additives of oleylamine (C_18_H_35_NH_2_, **b**), dodecylthiol (C_12_H_25_SH, **c**) and dodecylalcohol (C_12_H_25_OH, **d**). It is Se=PPh_2_H rather than SeTOP that reacts with Cd(OA)_2_ because of SeTOP+HPPh_2_⇌Se=PPh_2_H+TOP (refs 15, 23). The products **1a** (Ph_2_P–OOCC_17_H_35_) and **1b** (Ph_2_P–PPh_2_) equilibrate via Ph_2_P–COO*R* (**1a**)+HPPh_2_⇌*R*COOH+Ph_2_P–PPh_2_ (**1b**), which is weighted to the right at room temperature (RT)^21^,^24^. The additional products of **2a** (Ph_2_P(Se)–OOCC_17_H_35_) and **2b** (Ph_2_P(Se)–PPh_2_) from the mixtures of Cd(OA)_2_, Zn(OA)_2_ or Ge(OA)_2_+Se=PPh_2_H are shown in Supplementary Figs 3–5. The addition of a primary amine C_18_H_35_NH_2_ to the mixture of Fig. 1a, as shown in Fig. 1b, resulted in additional **1c** (Ph_2_P–NHC_18_H_35_) and **2c** (Ph_2_P(Se)–NHC_18_H_35_). The use of the thiol C_12_H_25_SH generated **1d** (Ph_2_P–SC_12_H_25_) without the detection of **1a** and **1b** (Fig. 1c). Similarly, the use of the alcohol C_12_H_25_OH produced **1e** (Ph_2_P–OC_12_H_25_) and **2e** (Ph_2_P(Se)–OC_12_H_25_; Fig. 1d). The same P-containing compounds were observed from the mixtures with Pb(OA)_2_ replacing Cd(OA)_2_ (Supplementary Fig. 5), which strongly suggests that Compounds **1a–e** (Ph_2_P–*Y*) have their own similar pathways (for different *Y*), and Compounds **2a–e** (Ph_2_P(Se)–*Y*) have their own similar pathways (for different *Y*). Thus, we propose that Compounds **1** and **2** follow two different paths for their formation from their own immediate precursors (Figs 1 and 2).

The temporal evolution of the absorption of growing CdSe NCs (shown in Supplementary Figs 6–7) suggests that the amount of additives, thiol C_12_H_25_SH or alcohol C_12_H_25_OH affects nucleation/growth, in addition to other experimental parameters such as the temperature and amount of HPPh_2_ used. Focusing on the identification of the reaction pathway before nucleation, the present study does not address the control of the size and size distribution, which could be affected by various experimental parameters including cation-to-anion feed molar ratios and the nature of Se=P*R*_2_H as shown by Supplementary Figs 8 and 9. In addition, the size and size distribution of the CdSe NCs synthesized with SeTOP+HP*R*_2_ (dicyclohexylphosphine (or HPCy_2_) and HPPh_2_) are different from those with Se=PCy_2_H and Se=PPh_2_H. Previously, the reaction of Cd(OA)_2_+Se=PCy_2_H was reported to lead to Compound Cy_2_P–OOCC_17_H_33_ (**1a** analogue) and Cy_2_P(Se)–OOCC_17_H_33_ (**2a** analogue)^24^. Therefore, the present study on the general reaction pathway from precursors to *M*_2_*E*_*n*_ monomers before nucleation at low reaction temperatures should benefit the field by leading to a better understanding of the ‘induction periods' to tailor, optimize and manipulate nucleation/growth, which offers finer control of the size and size distribution of NCs produced.

Figure 2 shows our ^31^P NMR spectra collected from four representative mixtures of Cd(OA)_2_+S=PPh_2_H (**a**) and with the additional additives of oleylamine (C_18_H_35_NH_2_, **b**), dodecylthiol (C_12_H_25_SH, **c**) and dodecylalcohol (C_12_H_25_OH, **d**). The chalcogenide S is generally less reactive than Se and Te under QD formation conditions^10^,^11^,^12^. Rather than employing an *in situ* generation of Se=PPh_2_H (Fig. 1 and Supplementary Figs 1–9) or Te=PPh_2_H (Fig. 3 and Supplementary Figs 14–21), the analogue S=PPh_2_H is sufficiently stable to be used directly (Supplementary Figs 10–13)^21^,^22^. The P-containing products detected from the S=PPh_2_H-related reactions with Cd(OA)_2_ (without additional HPPh_2_ but with free HPPh_2_ present) are similar to other chalcogenide-related reactions (Figs 1 and 3). Again, the products from the four reactions (Fig. 2) are grouped into Compounds **1** and **2**. For example, the products formed are elucidated as follows: **1a** without an additive (Fig. 2a), **1a** and **1c** with an amine additive (Fig. 2b), **1d** with a thiol additive (Fig. 2c) and **1e** and **1a** with an alcohol additive (Fig. 2d). The major difference between the Cd+S reactions (Fig. 2) and the Cd+Se reactions (Fig. 1) is the formation of **2b′** (Ph_2_P(S)–PPh_2_) under all conditions. Compound **2b′** was also detected from a mixture of Zn(OA)_2_+S=PPh_2_H shown in Supplementary Figs 10–12.

TeTOP is much more reactive than SeTOP and STOP in QD engineering^10^,^11^,^12^. Under the reaction conditions, this fact is readily discernible, as TeTOP (Supplementary Fig. 14) reacts completely when the first spectrum (1) of the each reaction shown in Fig. 3 was collected. Again, the products **1a** (Fig. 3a), **1c** (together with **1a**, in the presence of amine Fig. 3b), **1d** (in the presence of thiol Fig. 3c) and **1e** (in the presence of alcohol Fig. 3d) were detected in addition to **1b**. The same P-containing products were detected from the Ge(OA)_2_+TeTOP+HPPh_2_+H*Y* reactions (Supplementary Fig. 19). As shown in Supplementary Figs 15–17, the amount of HPPh_2_ used affects the ratio of **1a** and **1b** detected in the mixture of Cd(OA)_2_+TeTOP+HPPh_2_: the more HPPh_2_ is used, the more diphosphine compound **1b** is detected, the trend of which is similar to what was reported for CdSe because of the equilibrium of Ph_2_P−COO*R* (**1a**)+HPPh_2_⇌*R*COOH+Ph_2_P−PPh_2_ (**1b**) being weighted towards the right at RT^21^,^24^. Notably under these conditions, no Compound **2** (Ph_2_P(Te)–*Y*) was observed. The Te–P bond strength is lower than that of Se–P or S–P and, thus, Ph_2_P(Te)–*Y* might be too reactive to be detected.

For the S, Se and Te chalcogenide series with Cd (II) under all examined conditions (Figs 1, 2, 3), Ph_2_P–*Y* (**1a–e**) and/or Ph_2_P(*E*)–*Y* (**2a–e**) are identified as major P-containing products. For the other divalent metal salts of Zn, Ge and Pb studied, the same trends were discovered (Supplementary Figs 1–21). For *E*=Se (Supplementary Figs 1–2) in the absence of additional additives, **1a** and **1b** were predominantly found. With amine addition, **1c** and **2c** are also formed. With thiol addition, **1d** is formed as a main product, and with alcohol addition, both **1e** and **2e** are formed. For *E*=S, **1a–e** were detected along with **2b′** (Supplementary Figs 10–13). For *E*=Te, none of Ph_2_P(Te)–*Y* but **1a–e** were observed (Supplementary Figs 14–21). Thus, for all the combinations investigated, the reaction of *MX*_2_+*E*=PPh_2_H+H*Y* appeared to follow equation 1 to produce **1** and/or equation 2 to produce **2** along with the formation of *M*_2_*E*_2_ monomers.

More interestingly, the detection of P-containing compounds for Cu (I) and In (III) is similar to that for *M* (II). C_12_H_25_SH has been used as a solvent and a ligand to improve the synthesis of CuInSe_2_ and CuInS_2_ QDs^22^,^29^,^30^,^31^. Representative ^31^P NMR data for the synthesis of Cu_2_Se, In_2_Se_3_ and CuInSe_2_ using Se=PPh_2_H and S=PPh_2_H as the Se and S precursors are shown in Fig. 4, and for the synthesis of Cu_2_S, In_2_S_3_ and CuInS_2_ in Supplementary Figs 22 and 23. In the presence of CuI, C_12_H_25_SH and Se=PPh_2_H (Fig. 4a), **2d** (Ph_2_P(Se)–SC_12_H_25_) is formed as Se=PPh_2_H is consumed. The absence of **1d** (Ph_2_P–SC_12_H_25_) could be because of the equilibrium of **1d**+Se=PPh_2_H⇌**2d**+HPPh_2_ being weighted towards the right (Supplementary Fig. 1, Supplementary Table 2 and Supplementary Note 2). As mentioned before, **1d** and **2d** should have their own formation path. The addition of amine (Fig. 4b) leads to the product **2c** (Ph_2_P(Se)–NHC_18_H_35_), in addition to **1d** and salt Ph_2_P(Se_2_)NH_2_C_18_H_35_ (which can have resulted from the reaction of Se=PPh_2_H+C_18_H_35_NH_2_)^16^,^24^,^25^. The addition of alcohol (Fig. 4c) results in **2e** (Ph_2_P(Se)–OC_12_H_25_), in addition to **2d**. The absence of **1e** (Ph_2_P–OC_12_H_25_) could be because of the equilibrium of **1e**+Se=PPh_2_H⇌**2e**+HPPh_2_ being weighted towards the right (Supplementary Fig. 2, Supplementary Table 2 and Supplementary Note 2).

Indium, as expected, is more reactive under the same reaction conditions where Se=PPh_2_H completely reacts at 80 °C per 10 min (Figs 4d–2) compared with Fig. 4a^22^. Intriguingly, at RT per 15 min (Fig. 4e with expansion), the additional peaks near free Se=PPh_2_H (∼7.3 p.p.m.) are readily interpreted as coordinated Se=PPh_2_H to In (Fig. 4e and Supplementary Fig. 22). The products **1d** and **2d** are observed with the absence of Se=PPh_2_H (Fig. 4d, the In-only experiment), whereas only **2d** is formed in the reaction with the presence of Se=PPh_2_H (Fig. 4f with both the presence of Cu and In). Thus, the observation of Compounds **1** and **2** could be affected by several factors, including the equilibrium of **1**+Se=PPh_2_H⇌**2**+HPPh_2_, which could be weighted towards the right (Supplementary Table 2 and Supplementary Note 2), similar to TOP+Se=PPh_2_H⇌SeTOP+HPPh_2_ (refs 15, 23), except for **1b**+Se=PPh_2_H⇌**2b**+HPPh_2_ (refs 21, 23, 24, 25).

It is critical to perform additional experimental investigation regarding the formation of Compound **2** from a direct path. Figure 5 shows the corroborative evidence for equation 2 (Figs 6 and 7 and Supplementary Figs 1–9). These experiments relied on the independent preparation of cadmium bis(diselenophosphinate) (Cd(Se_2_PPh_2_)_2_, **3**)^24^, which reacted with Cd(OA)_2_ in the presence of H*Y* of C_18_H_35_NH_2_ (**a**), C_12_H_25_SH (**b**) and C_12_H_25_OH (**c**). The reaction of **3**+Cd(OA)_2_+H*Y* leads to **2c**, **2d** and **2e**, respectively. It is noteworthy that **1** was not detected. The addition of oleic acid did not lead to **2a** (not shown). These results suggest that equation 2 is active at the appropriate temperatures tested (with the amine, thiol and alcohol, but not with the acid). According to the previous study on **3**+Cd(OA)_2_ (ref. 24), it is reasonable that the presence of HPPh_2_ could speed up equation 2. As shown by Supplementary Fig. 24, the catalytic amount of HPPh_2_ (0.05 eq. based on **3**) accelerated significantly each of the three reactions, with **2** still being the main product. With more HPPh_2_ (1.00 eq. based on **3**), additional **1c** (with **1b**), **1d** (with **1a** and **2a**) and **1e** were detected, respectively. Thus, HPPh_2_ could also initiate another equation 1 to **1**+Cd_2_Se_2_ (via **A** (Cd(SePPh_2_)_2_) as shown in Supplementary Fig. 25). The results shown by Fig. 5 and Supplementary Fig. 24 clearly support that the equilibrium of **1e**+Se=PPh_2_H⇌**2e**+HPPh_2_ is weighted towards the right, which is in agreement with our DFT examination shown in Supplementary Table 2 and Supplementary Note 2.

Figure 6 presents a schematic interpretation of our experimental results (shown in Figs 1, 2, 3, 4, 5 and Supplementary Figs 1–24). When a mixture of metal carboxylate and chalcogenide TOP compound (*E*TOP) was mixed with dialkylphosphine such as HPPh_2_ with or without the presence of additives such as amines, thiols and/or alcohols, the formation of NCs begins with chalcogen *E* exchange, namely *E*TOP+HPPh_2_⇌TOP+*E*=PPh_2_H (refs 15, 23, 24, 25), the exchange of which activates the chalcogenide. Subsequently, the activated *E*=PPh_2_H reacts by coordinating the chalcogenide atom to the metal centre (equation 3). In the absence of HPPh_2_, the dioctylphosphine impurity in TOP could play the same chemical function, which was only realized recently^15^,^17^,^18^,^19^,^20^,^21^,^22^,^23^.


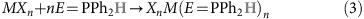



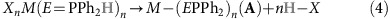



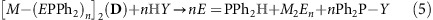



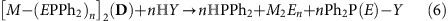


Following the coordination (equation 3), as experimentally demonstrated by Fig. 4e, intermediate **A** (*M*–(*E*PPh_2_)_*n*_) is formed, accompanied by H−*X* (equation 4). It seems reasonable that the H–P bond of *E*=PPh_2_H is strong enough to sustain the coordination to the metal, but weak enough for H to leave and to form H*X*. The chalcogen coordination to the metal not only increases the acidity of the P-bound H but also makes H accessible to the adjacent *X* group. Thus, in the forward reaction direction, the P-bound H leads to the elimination of the ligand *X* resulting in **A** and H*X* (equation 4). In the next step, we propose **A** reacts with H*Y* first to give **B** and/or **C**, which then dimerize towards **E** and/or **F**, respectively. At the same time, **A** could first dimerize towards **D**, [*M*−(*E*PPh_2_)_*n*_]_2_, and then reacts with **HY** to give **E** and/or **F**. For example, dimer **D** reacts with H*Y* to produce *E*=PPh_2_H+*M*_2_*E*_*n*_+Ph_2_P−*Y* (**1**; equation 5) or HPPh_2_+*M*_2_*E*_*n*_+Ph_2_P(*E*)−*Y* (**2**; equation 6). Our proposed pathway leading to the observation of **1** and **2** significantly differs from the pathway proposed in 2010 (ref. 15), as detailed in Supplementary Fig. 26 and Supplementary Note 3. One major difference is intermediate **A** and its formation and subsequent evolution (equations 3, 4, 5, 6), which were not addressed in the work of 2010 but are clearly elucidated in the present study. Note that the pathway proposed in 2010 does not address at all the detection of **1b** (PPh_2_−PPh_2_, 14 p.p.m.) from the reactions of Pb(OA)_2_+Se=PPh_2_H and Cd(OA)_2_+Se=PPh_2_H at RT.

Figure 6 is formulated for the case of *M* (II), but also applies to *M* (I) and *M* (III) where their monomers are accordingly proposed to be *M*_2_*E* and *M*_2_*E*_3_, respectively. Obviously, **A** and **B** are connected by equilibrium **A**+H*Y*⇌**B**+*E*=PPh_2_H, while **A** and **C** by **A**+H*Y*⇌**C**+HPPh_2_. Consequently, **B** and **C** are correlated by **B**+*E*=PPh_2_H⇌**C**+HPPh_2_, similarly to Compounds **1** and **2** by **1**+*E*=PPh_2_H⇌**2**+HPPh_2_. These equilibria are clearly affected by the relative amount of HPPh_2_, *E*=PPh_2_H and H*Y*. The formation of the monomer *M*_2_*E*_2_ occurs via the ligand loss of **1** from intermediate **E** (equation 1) and/or **2** from intermediate **F** (equation 2). With *Y*=PPh_2_, intermediate **D** in nature is **F**; thus, **D** can result in **2b**+*M*_2_*E*_*n*_ (equation 2).

### DFT study

To further understand the fundamental chemistry involved in the putative pathway proposed in Fig. 6, let us turn our attention to the possible isomers with their bonding skeletons of each of the intermediate species **A** to **F** proposed in Fig. 6. In addition to metal ions (*M*), chalcogenides (*E*) and diphenylphosphinio species (Ph_2_P), intermediates **B** to **F** contain the various *Y* groups. Consequently, each intermediate has multiple possible constitutional isomers, while most possible combinations of bonds, such as P–*E*, *E*–*E*, P–P, P–*Y* and *E*–*Y* bonds, exist in well-known compounds, and all such bonds can in principle coordinate to metal ions leading to multiple possibilities. For example, for *Y*=NHR in Fig. 6, the N could bond to Cd, P or Se; if N is bound to P, two bonding arrangements (*M*–P–N and *M*–N–P) could in principle be expected. These uncertainties are amenable to DFT calculations, which provide useful information to minimize positional isomers, with the cancellation of errors in the DFT approximation^39^,^40^,^41^,^42^. In this way, the calculated bonding trends should be reliable. The possibilities in Fig. 7 are distinguished by DFT calculations at the M06//B3LYP/6-31++G (d, p), Stuttgart/Dresden (SDD) level in ODE media. Our DFT-calculated structures and energies of many more possible isomers are shown in Supplementary Tables 3–25 including structural, geometric and rotational isomers. An additional description and discussion of the isomers of each intermediate **A** to **F** can be found immediately before Supplementary Tables 3–17.

The most stable **A** is with the P–Se–Cd–Se–P skeleton among the seven isomers computed (Supplementary Table 3). For the two predominant species **B1** and **B2** found, they have the Se–Cd–**P**–***Y*** and P–Se–**Cd**–***Y*** skeletons, respectively. **B1** versus **B2** includes P–*Y* versus *M*–*Y* bonds, without *E*–*Y* bonds. With *Y*=NH*R* for CdSe (Supplementary Table 4), the **B1** isomer was calculated to be 10.1 kJ mol^−1^ (free energy Δ*G*) more stable than **B2**. This energy trend of **B1**<**B2** was not found for the other *Y*. For *Y*=S*R* (Supplementary Table 4), the distinction is quite clear that the direct metal-bound **B2** isomer P–Se–**Cd**–**S*R*** was calculated to be 82.5 kJ mol^−1^ more stable than the **B1** isomer with Se–Cd–**P**–**S*R***. For *Y*=O*R* (Supplementary Table 5), the **B2** isomer with the P–Se–**Cd**–**O*R*** skeleton was found to be at the lowest energy, but the **B1** isomer with the Se–Cd–**P**–**O*R*** skeleton was only 6.9 kJ mol^−1^ higher—an energy difference that is close to the accuracy of the DFT method and could be affected by the exact nature of various *R* groups, the solvent used and the temperature employed. For *Y*=OOC*R* (Supplementary Table 6), the directly metal-bound **B2** isomer is 111.3 kJ mol^−1^ more stable than the **B1** isomer with Se–Cd–**P**–**OOC*R***. For *Y*=PPh_2_ (Supplementary Table 6), **B2** is 58.7 kJ mol^−1^ more stable than **B1**.

Intermediate **C** with an extra chalcogen *E* atom compared with intermediate **B** evidently has more constitutional possibilities. Intriguingly, the connectivity follows similar patterns to that of intermediate **B**. For *Y*=NH*R* (Supplementary Table 7), **C1** with the Se–Cd–Se–**P**–N connectivity has the lowest energy. Note that there is an extra Se inserted between the Cd and P atoms. The most stable Cd–N-bound species **C2** was found to contain the four-membered N–Cd*–Se–P–Se–(Cd*) ring, which was 18.7 kJ mol^−1^ calculated. For *Y*=S*R* (Supplementary Table 8), **C2** with a direct Cd–SR bond is favoured much more than the other isomers considered. Complexes with this **C2-**type connectivity but with *Y*=SSPPh_2_ have been characterized experimentally^35^. For *Y*=O*R* (Supplementary Table 9), **C1** and **C2** differ by only 2.3 kJ mol^−1^ and can therefore be considered iso-energetic. For *Y*=OOC*R* (Supplementary Table 10), **C2** with the direct Cd–OOC*R* bonding is much more stable, similar to the case of *Y*=S*R*.

Consequently, for *Y*=NH*R*, **B1** and **C1** are preferred. For *Y*=O*R*, the selectivity is not obvious. For *Y*=S*R* and OOC*R*, **B2** and **C2** are favoured. Thus, the preference on the bonding skeleton calculated for intermediates **B** and **C** is similar. The nature of the chalcogenide also affects the relative stability of **B1** versus **B2** (Supplementary Table 18) as well as that of **C1** versus **C2** (Supplementary Table 19). For *Y*=NH*R* specifically, **B2** and **C2** are stabilized for *E*=S, whereas **B1** and **C1** are more stable for *E*=Te than for *E*=Se. For CdSe, our preliminary efforts on the kinetics associated with the putative pathway **A**+H−*Y*→**B**+Se=PPh_2_H are presented in Supplementary Figs 28–31 and Supplementary Note 5 with **A1b** for **A** and **B2a** for **B**. The trend of the kinetics computed seems to be in agreement with our experimental data showing the slowest disappearance of SeTOP (Fig. 1) and of **3** (Fig. 5) is from the batch with H*Y*=*R*NH_2_.

Intermediates **E** and **F** are proposed as the very immediate precursors leading to monomers *M*_2_*E*_*n*_ with Compounds **1** and **2**, respectively. **E1** could have resulted from dimerization of **B1** and **E2** from **B2**. **F1** could have resulted from dimerization of **C1** and **F2** from **C2**. Again, DFT calculations were performed to address the question of whether the various *Y* species are bound to Cd or to Se or to P. Clearly, **E** and **F** are computationally demanding. Generally, **E** isomers follow the trend of **B** isomers, and **F** follows **C**: low-energy **B** isomers lead to low-energy **E**, and **C** to **F**. In all cases, the four-membered ring Cd*–Se–Cd–Se–(Cd*) was found by minimization. For *Y*=NH*R* (Supplementary Table 11), **E1** with the P–N bond is much more stable than **E2** with the Cd–N bond by ∼150 kJ mol^−1^. For *Y*=S*R* (Supplementary Table 11), **E2** with the Cd–S bond is more stable than **E1**, but the difference is smaller (32.7 kJ mol^−1^) than that (82.5 kJ mol^−1^) of **B2** versus **B1**. For *Y*=O*R* (Supplementary Table 12), **E1** is much more stable than **E2**, whereas **B1** is similar to **B2**. For *Y*=OOC*R* (Supplementary Table 13), isomers such as **E2** (with the Cd–*Y* bond) are the most stable ones found.

Intermediate **F** consists of two more *E* atoms than **E**. For *Y*=NH*R* (Supplementary Table 14), **F1**, with Se inserted between the Cd and P, namely Cd–Se–P–N, is 196.6 kJ mol^−1^ more stable than **F2** with the direct Cd–N bond. For *Y*=S*R* (Supplementary Table 15), **F2** is 78.0 kJ mol^−1^ more stable than **F1**. For *Y*=O*R* (Supplementary Table 15), **F1** is 126.1 kJ mol^−1^ more stable than **F2**. For *Y*=OOC*R* (Supplementary Table 16), **F2** is 78.8 kJ mol^−1^ more stable than **F1**. For *Y*=PPh_2_, **D2** (a dimer of **A**) is more stable than **D1** (equivalent to **F1**) by 86.6 kJ mol^−1^.

Although speculative, our current proposal is that **E** and **F** (or possibly higher oligomers such as from the dimerization of **E** and **F**)^43^ facilitate the release of **1** and **2**, respectively. The release of Compound **1** is more apparent from **E1** (via the *M*–P bond cleavage) than from **E2**; the Cd–P bond expected to break for **E1** to lose **1** has a length of 2.66 Å (longer than 2.58 Å in **B1**). In addition, the release of Compound **2** is more apparent from **F1** (via the *M*–*E* bond cleavage) than from **F2**; the Cd–Se bond expected to break for **F1** to lose **2** has a length of 2.74 Å (longer than 2.69 Å in **C1**). For the release of **1** and **2** from **E2** and **F2**, respectively, it seems reasonable that the formation of a *Y*–P bond (via the interaction of *Y* with Ph_2_P and with Ph_2_P(*E*)) could be accompanied by the cleavage of *M*–*Y* and P–*E* bonds^44^. It has been suggested that the oligomerization to [Cd_2_Se_2_]_*m*_ is accompanied by a decrease in free energy for at least *m*=6 (ref. 24); this thermodynamic stability of [*M*_2_*E*_*n*_]_*m*_ may be the driving force of the overall reaction^45^.

## Discussion

The molecular pathway of precursor evolution to monomers responsible for nucleation at low reaction temperature to semiconductor NCs has been recognized as a major challenge in advancing the design and synthesis of high-quality NCs with high synthetic reproducibility and particle yield. We have successfully rationalized a general reaction pathway for precursor evolution to monomers at low reaction temperatures from the mixture of *MX*_*n*_+*E*TOP+HPPh_2_+H*Y* or *MX*_*n*_+*E*=PPh_2_H+H*Y*. On the basis of the experimental and computational investigations, we propose the monomer of *M*_2_*E*_*n*_ and its formation accompanied by the loss of ligand Ph_2_P–*Y* (**1**) and Ph_2_P(*E*)–*Y* (**2**) via two competing paths. Experimentally, the combination of six metal ions of monovalent, divalent or trivalent, three chalcogenides and five types of additive H*Y* (of carboxylic acid, dialkylphosphine, amine, thiol or alcohol) results in the P-containing products of Ph_2_P–*Y* (**1**) and Ph_2_P(*E*)–*Y* (**2**). The in-depth interpretation of the mechanism is supported by our DFT calculations. Our proposed pathway features a series of H-mediated ligand loss/exchange reactions triggered by dialkylphospine chalcogenides (such as *E*=PPh_2_H) to form intermediate **A** (*M*–(*E*PPh_2_)_*n*_), which leads to intermediate **E** (*Y*Ph_2_P–*ME*_*n*_*M*–PPh_2_*Y*, equation 1) and intermediate **F** (*Y*Ph_2_P*E*–*ME*_*n*_M–*E*PPh_2_*Y*, equation 2), the formation of which consists of dimerization and reaction with H*Y*. The disassociation of ligand **1** from **E** and ligand **2** from **F** results in *M*_2_*E*_*n*_ monomers. Clearly, H*Y* participates in the formation of monomers and thus could accelerate nucleation; meanwhile, a large amount of H*Y* plays the role of a solvent and, thus, could retard nucleation. Importantly, the general pathway applies to metal chalcogenide NCs made from both toxic metals such as Cd (II) and Pb (II) and more benign metals such as Cu (I), Zn (II) and In (III). The insights into the chemical nature of the *M*_2_*E*_*n*_ monomer the building block, could provide the basis for the field to enable the manipulation of the chemical processes for rational design and synthesis of a variety of NCs with complex stoichiometry. The use of secondary phosphines together with beneficial additive H*Y* should be a general and practical avenue to engineer metal chalcogenide NCs at low reaction temperatures with high quality, enhanced synthetic reproducibility and particle yield. We anticipate that the insight gained on the molecular pathway for precursor evolution into various types of *M*_2_*E*_*n*_ monomers may enable the field to synthesize sophisticated NCs, including phase-change materials, with better-controlled chemical processes via cation exchange as well as doping and co-doping with monovalent and trivalent metal ions^46^,^47^,^48^,^49^,^50^,^51^,^52^. We are actively exploring the correlation between the pathway of monomer formation with the formation of magic-size and regular QDs, aiming at the control of product properties including the size and size distribution. In addition, we believe that, similar to the endeavour of the development of organic syntheses, the basic chemistry reported embraces the advance of the NC synthesis from an empirical art to science with pathway-enabled design leading towards the full realization of the NC potential^53^,^54^,^55^,^56^,^57^.

## Methods

### ^31^P NMR measurements

^31^P NMR was performed on a Bruker AV-III 400 spectrometer operating at 161.98 MHz, referenced with an external standard, 85% H_3_PO_4_. Usually, we used D1=2 s (64 scans total taking ∼3 min; unless mentioned otherwise). NMR samples were usually prepared and loaded in NMR tubes in a glovebox and properly sealed. All chemicals used are commercially available from Sigma-Aldrich and were used as received (or otherwise specified). The used ligands and additives are oleic acid (OA, tech. 90%), diphenylphosphine (HPPh_2_, 99%, Strem Chemicals), oleic amine (OLA, C_18_H_35_NH_2_, tech. 70%), 1-dodecanethiol (C_12_H_25_SH, 98%) and lauryl alcohol (C_12_H_25_OH, 98%). The elemental chalcogens used are sulfur (S, precipitated, Anachemia), selenium (Se, 200 mesh, 99.999%, Alfa Aeser) and tellurium (Te, 200 mesh, 99.8%). For the assignment of Compounds **1** and **2**, sodium hydride (NaH, 95%, dry), chlorodiphenylphosphine (Ph_2_P–Cl, 97%, Alfa Aeser) were used. Compounds **1** (Ph_2_P–*Y*) and **2** (Ph_2_P(*E*)−*Y*) detected with NMR are related to the formation of monomers/solutes/NCs, and have been use to explore the formation of monomers since 2006 (refs 13, 15, 23, 24, 25). The P-containing products detected with ^31^P NMR are listed as follows: **1a** (Ph_2_P–OOCC_17_H_33_), **1b** (Ph_2_P–PPh_2_), **1c** (Ph_2_P–NHC_18_H_35_), **1d** (Ph_2_P–SC_12_H_25_), **1e** (Ph_2_P–OC_12_H_25_), **2a** (Ph_2_P(Se)–OOCC_17_H_33_), **2b** (Ph_2_P(Se)–PPh_2_), **2b′** (Ph_2_P(S)–PPh_2_), **2c** (Ph_2_P(Se)–NHC_18_H_35_), **2d** Ph_2_P(Se)–SC_12_H_25_) and **2e** Ph_2_P(Se)–OC_12_H_25_).

### Computational

Our DFT calculations were performed using Gaussian 09, with ethyl groups (-C_2_H_5_) applied to represent the alkyl group of C_17_H_33_COO-, C_18_H_35_NH-, C_12_H_25_S- and C_12_H_25_O-; no simplicity was applied for the phenyl group of -PPh_2_. Full geometry optimizations were carried out to locate all of the stationary points via a hybrid B3LYP functional method with the SDD basis set and the corresponding effective core potential for the Cd, Se and Te atoms, and the all-electron 6-31++G(d, p) basis set for the other atoms of C, H, O, N, P and S, namely B3LYP/6-31++G(d, p), SDD. The use of effective core potential and all-electron basis was the same as before^25^. Systematic harmonic frequency calculations were performed to ensure that all the structures obtained are true minima on the potential energy surfaces. A polarized continuum model (PCM-SMD) with dielectric constant *ɛ*=2.0 was utilized to simulate the solvent effect of ODE via a hybrid M06 functional method with the same basis sets as mentioned above by performing single-point calculation on the optimized structures at the B3LYP/6-31++G(d, p), SDD level, namely M06//B3LYP/6-31++G(d, p), SDD. The charges and dominant occupancies of natural bond orbitals have been analysed with the help of the natural bond orbital analysis.

### Data availability

The authors declare that all relevant data supporting the findings of this study are available from the authors on request.

## Additional information

**How to cite this article:** Yu, K. *et al*. General low-temperature reaction pathway from precursors to monomers before nucleation of compound semiconductor nanocrystals. *Nat. Commun.* 7:12223 doi: 10.1038/ncomms12223 (2016).

## Supplementary Material

Supplementary Information Supplementary Figures 1-31, Supplementary Tables 1-25, Supplementary Notes 1-5, Supplementary Methods, Supplementary Discussion and Supplementary References.
