# Supplementary material for: General low-temperature reaction pathway from precursors to monomers before nucleation of compound semiconductor nanocrystals
Source: Nat Commun. 2016 Aug 17;7:12223. doi: 10.1038/ncomms12223 (PMC4992053; doi:10.1038/ncomms12223)
Supplement: Supplementary Information — Supplementary Figures 1-31, Supplementary Tables 1-25, Supplementary Notes 1-5, Supplementary Methods, Supplementary Discussion and Supplementary References. [file ncomms12223-s1.pdf]

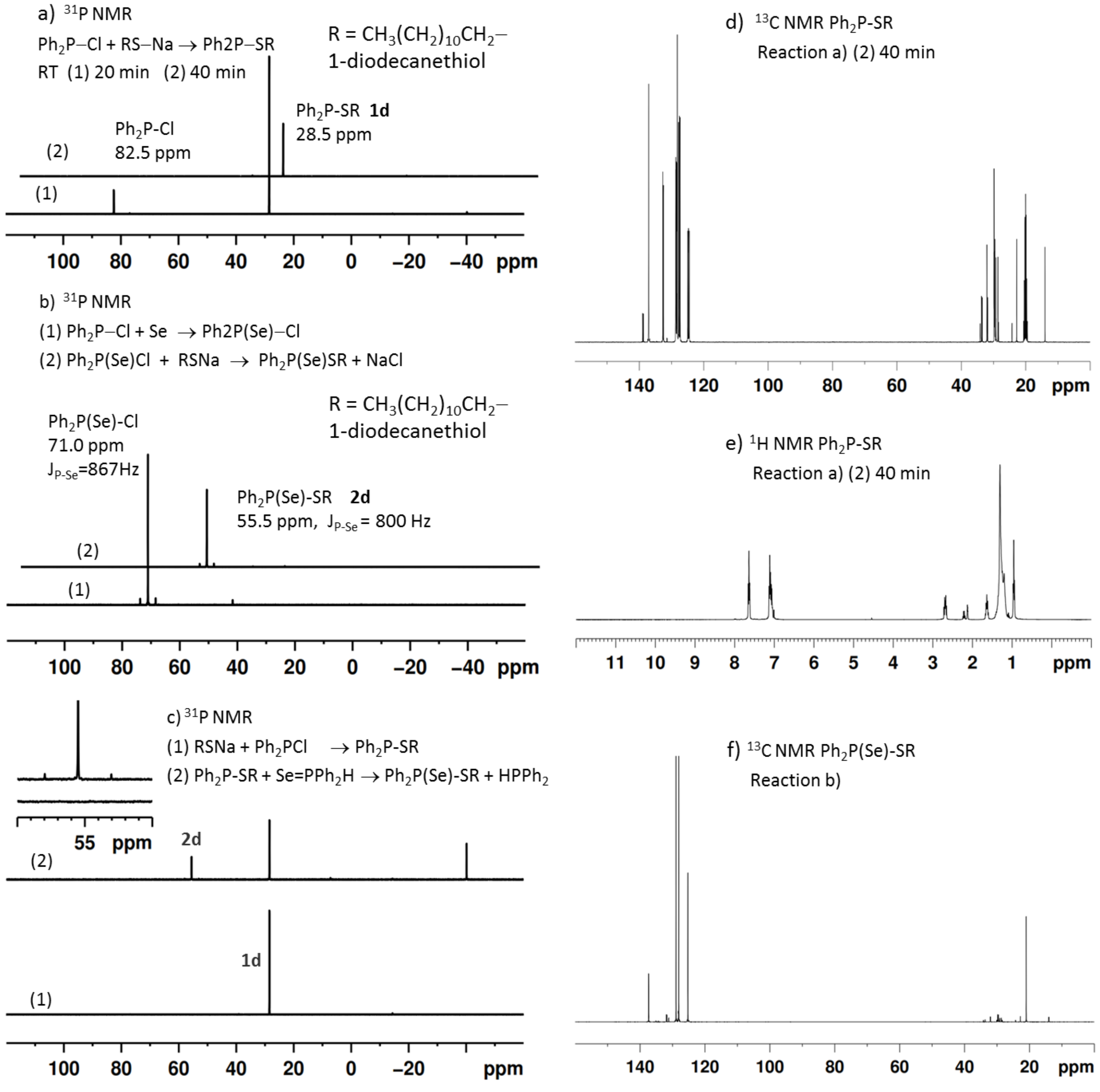

Supplementary Figure 1. NMR of the mixtures indicated.

a) ^{31}PNMR shows a mixture of Ph_2PCl and $Ph_2P–SC_{12}H_{25}$ (**1d**). NaH (1.641 mmol, 39.4 mg) was mixed with $C_{12}H_{25}SH$ (1.65 mmol, 334 mg, d= 0.845 g/ml) to give $C_{12}H_{25}SNa$ as a white solid. Then, $C_{12}H_{25}S–Na$ (0.125 mmol, 28 mg) in Tol_d8 was added to Ph_2Cl (0.12 mmol, 26.5 mg) at RT, and a colorless solution with white precipitate formed[1]. To the NMR tube, additional 0.3 mg NaH and 3 μl $C_{12}H_{25}SH$ (0.0125 mmol, around 1% extra) added. ^{31}PNMR shows complete conversion of Ph_2PCl to $Ph_2P–SC_{12}H_{25}$.

b) Se (2.052 mmol, 160 mg) was mixed with Ph_2PCl (2.017 mmol, 445 mg), and heated to get an almost clear brown-yellow solution. After cold 2.1988 g of toluene was added, the mixture was heated for 10min. A brown yellow solution (0.72 mmol/g) was obtained. Then, 0.2155 g (0.16 mmol) mixture in 0.4 ml of toluene was taken for ^{31}PNMR measurements. Meanwhile, NaH (0.17 mmol, 4.1 mg) was mixed with $C_{12}H_{25}SH$ (0.165 mmol, 33.4 mg) to give $C_{12}H_{25}SNa$ as a white solid. 0.2 ml of toluene was added, which was mixed with the NMR sample of $Ph_2P(Se)Cl$. ^{31}PNMR indicates that $Ph_2P(Se)S–C_{12}H_{25}$ (**2d**) was formed but not to completion. Therefore ca 20% additional $C_{12}H_{25}SNa$ was added to completed the reaction.

c) Again, **1d** was prepared, and reacted with $Se{=}PPh_2H$.

d) and e) ^{13}C NMR and ^{1}HNMR of **1d**, respectively. f) ^{13}CNMR of **2d**.

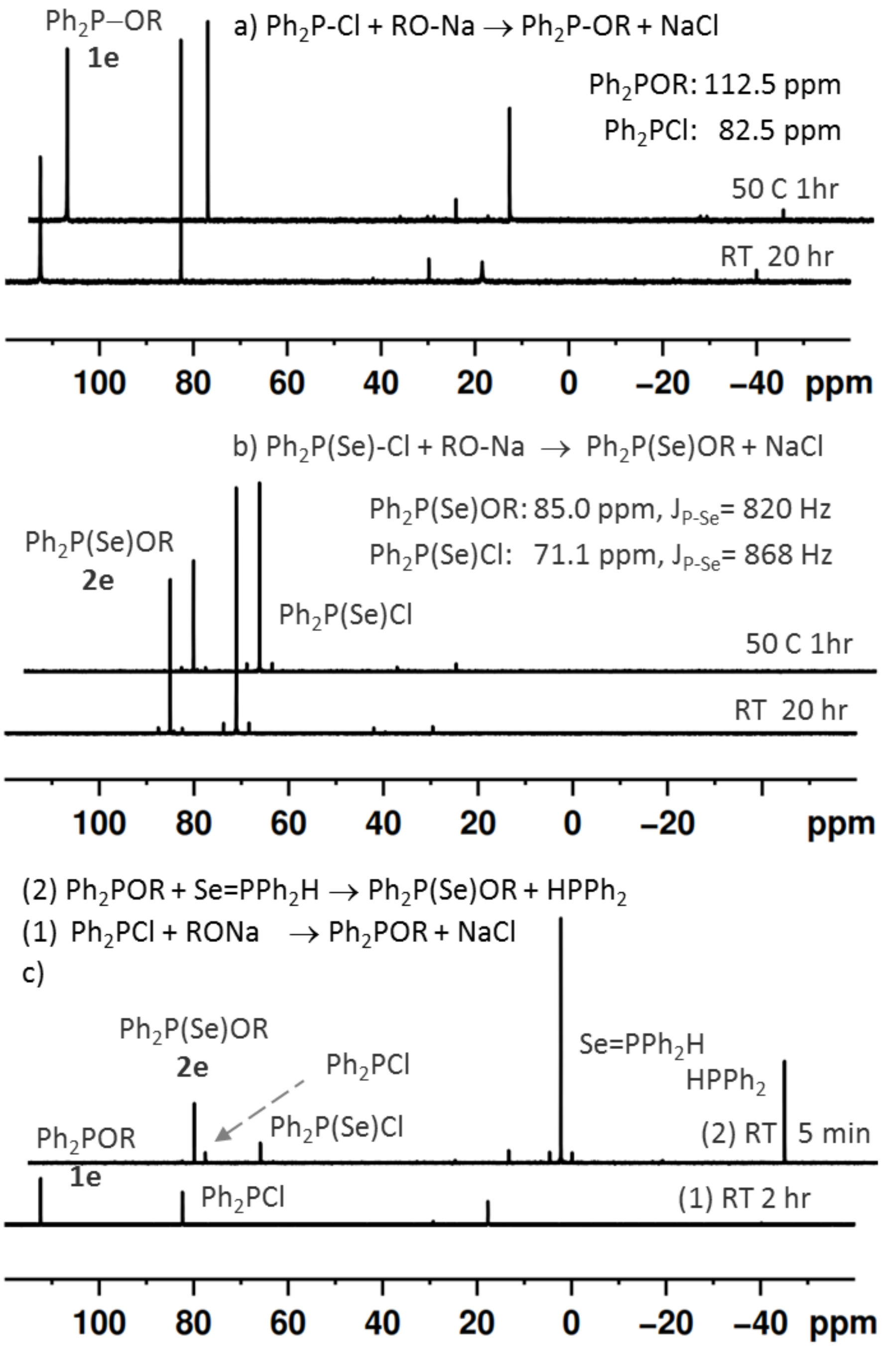

Supplementary Figure 2. NMR of the mixtures indicated.
a) A mixture of $C_{12}H_{25}OH$ (65.20 mg, 0.35 mmol) and NaH (9 mg, 0.375 mmol) was heated gently. After cooled to RT, 1.1 g tol was added to give a suspension of RONa (= 0.318 mmol/g in tol). To a suspension of $C_{12}H_{25}ONa$ (0.05 mmol, 159.2 mg of 0.318 mmol/g suspension in Tol) was added Ph_2PCl (0.046 mmol, 10.2 mg), giving a white suspension of **1e**.

b) To a suspension of $C_{12}H_{25}ONa$ (0.05 mmol, 161 mg of 0.318 mmol/g suspension in tol) was added, a yellow solution of $Ph_2P(Se)Cl$ (0.047 mmol, 54mg of 0.874 mmol/g solution in tol) was obtained giving a white/yellow suspension of **2e**.
c) The Se exchange from $Se{=}PPh_2H$ to **1e**.

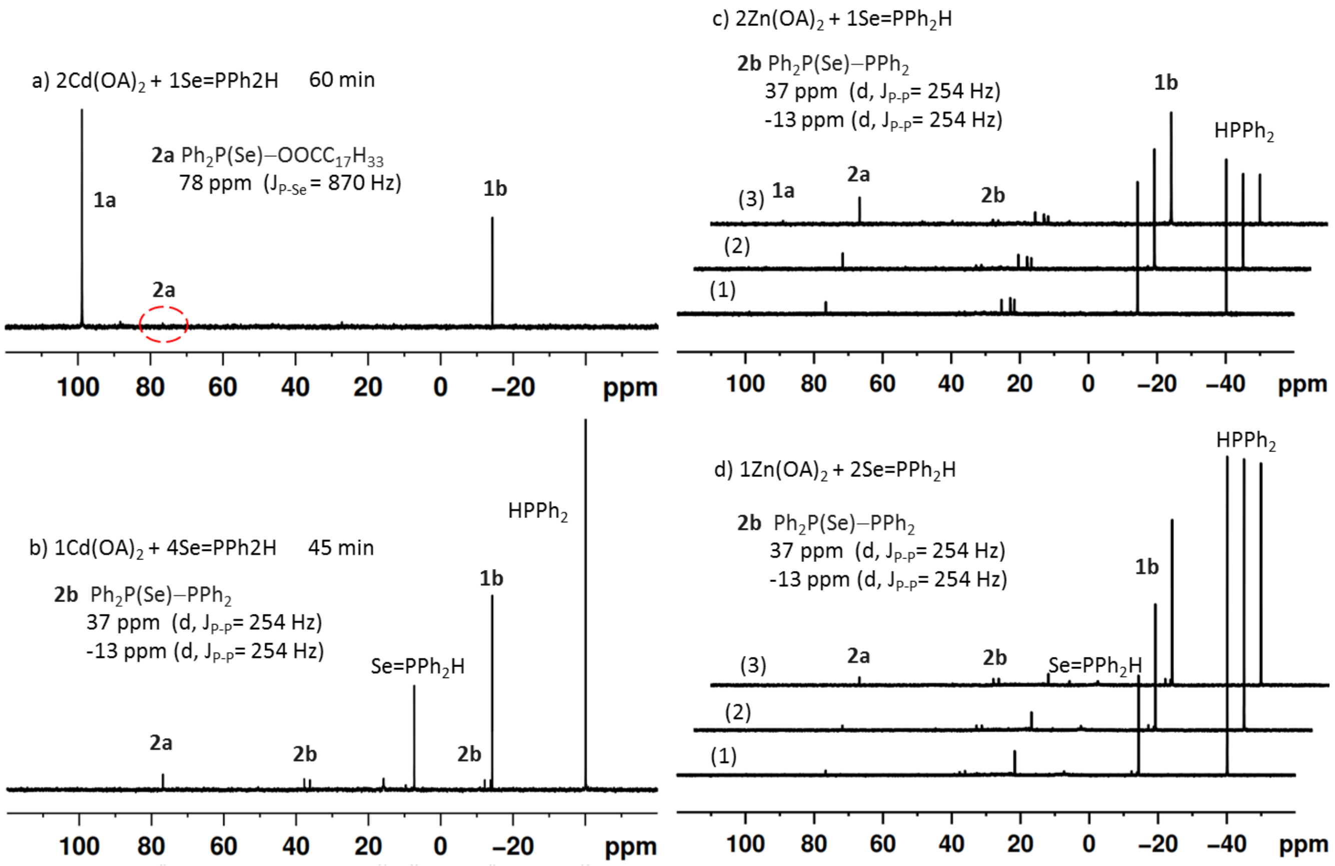

Supplementary Figure 3. ^{31}P NMR spectra collected from the four mixtures indicated.
a) RT/60 min and [Se] = ~30 mmol/Kg.
b) RT/45 min and [Se] = ~30 mmol/Kg.
c) RT/15-30-60 min and [Se] = ~40 mmol/Kg.
d) RT/15-30-60 min and [Zn] = ~40 mmol/Kg.

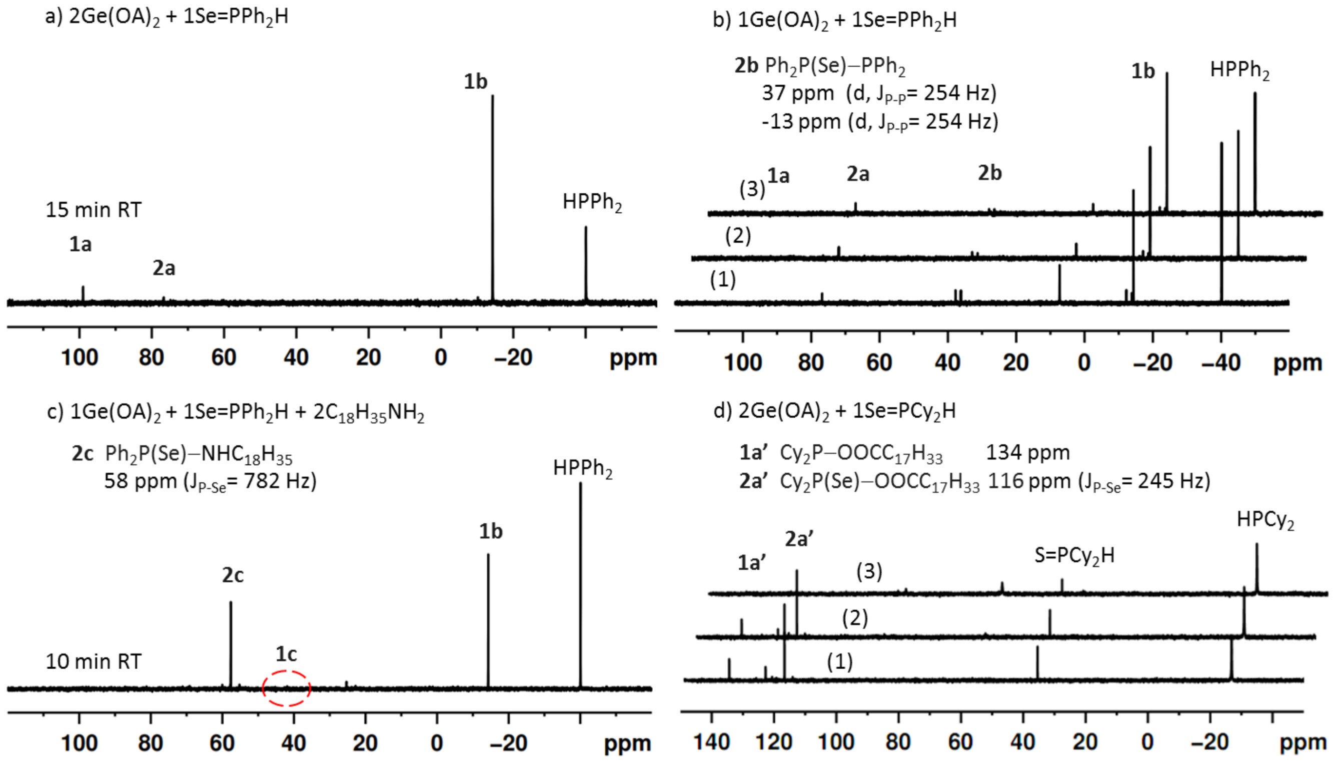

Supplementary Figure 4. ^{31}P NMR spectra collected from the four mixtures indicated. [Se] = 25 mmol/Kg.

a) RT/15 min.
b) RT/10-60-210 min.
c) RT/10 min.
d) RT/60 min-RT/180 min-80 °C/30 min. Dicyclohexylphosphine (DCHP, 97 % Sigma-Aldrich) was used to prepare $Se=PCy_2H$[2].

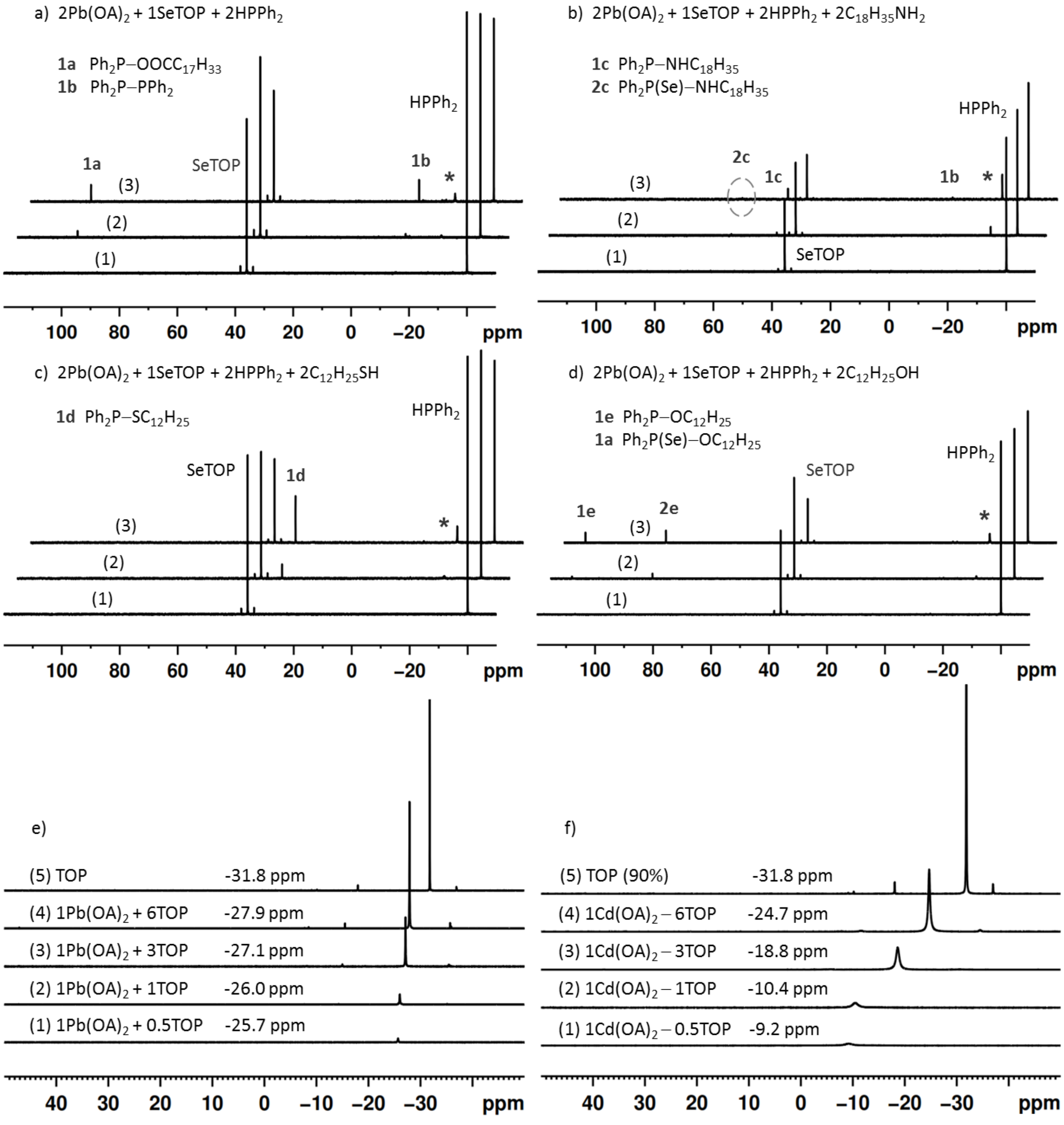

Supplementary Figure 5. ^{31}P NMR spectra collected from the mixtures indicated. [Se] = 16 mmol/Kg. a-d) RT/30 min (1) – 60 °C/30 min (2) – 80 °C/30 min (3). e) [Pb] = 33 mmol/Kg. Pb coordination to TOP is less effective as compared to Cd to TOP shown in f). For the RT/30 min data,
a) SeTOP 36.1 ppm, $J_{P\text{-}Se}$ = 712 Hz. b) SeTOP 35.7 ppm, $J_{P\text{-}Se}$ = 720 Hz.
c) SeTOP 35.9 ppm, $J_{P\text{-}Se}$ = 715 Hz. d) SeTOP 36.0 ppm, $J_{P\text{-}Se}$ = 714 Hz. Note that the ^{31}P NMR signal of SeTOP fluctuates for each of the four batches a) to d). To analyze an ongoing reaction with the

change of temperature, the use of the product-to-reactant ratio should be a practical way (rather than the increase of a product or the decrease of a reactant). Here, it is the product-to-SeTOP ratio that should be compared for each of the three spectra collected for Batches a) to d). In such a sense, the amount of SeTOP decreased in each of the batch as the temperature increased.

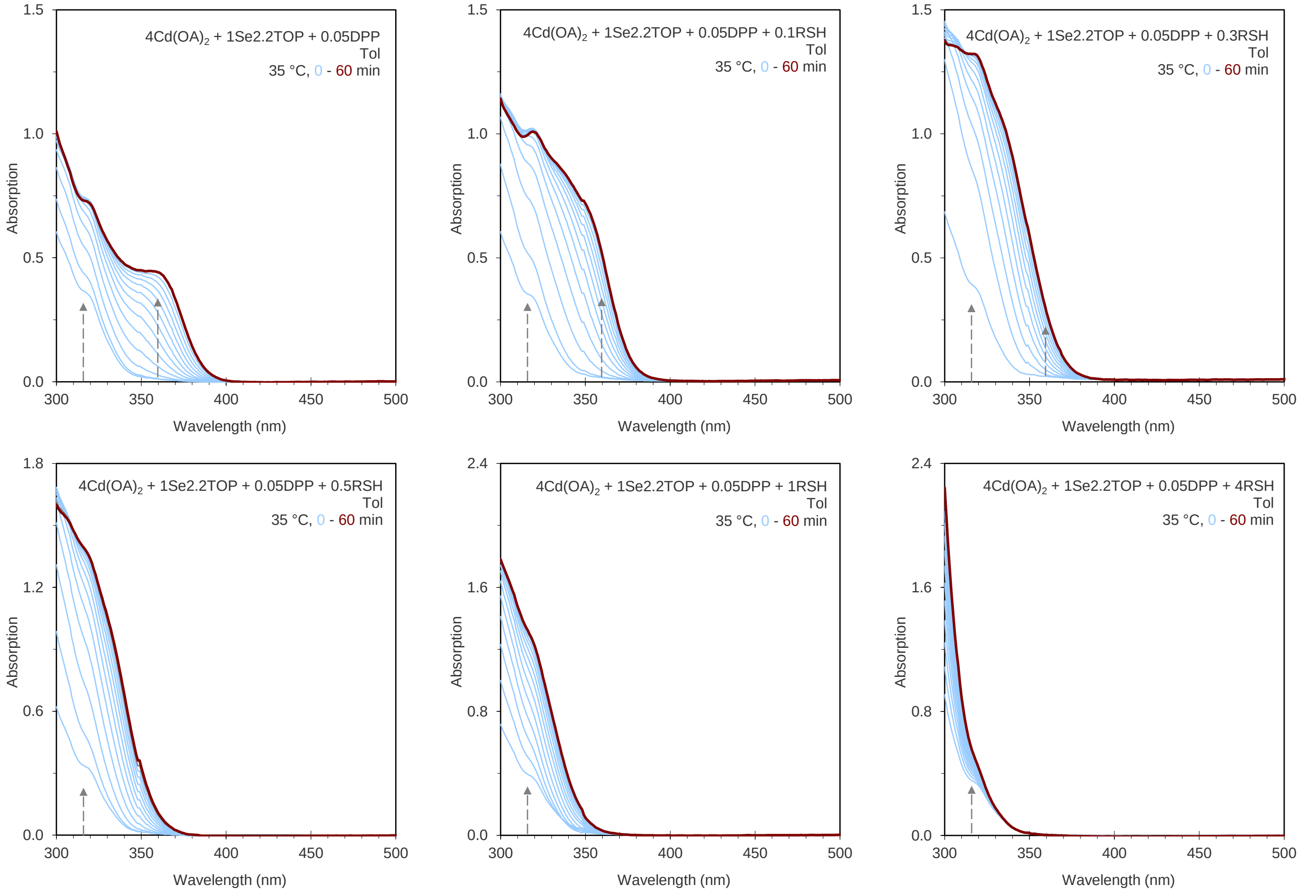

Supplementary Figure 6. The temporal evolution of absorption of growing CdSe NCs from the batches at 35 °C with the use of $0.05HPPh_2$ to illustrate the promotion or inhibition by the additive of $R_{12}H_{25}SH$ on nucleation/growth. The spectra were collected (by Cary 300 with an optical light path of 2 mm) from the 4Cd-to-1Se molar ratio batches with [Se] of 17 mmol kg^{-1} in ODE and a total weight of ~2.3 g. Commercial 90 % TOP was used to make our 1M SeTOP.

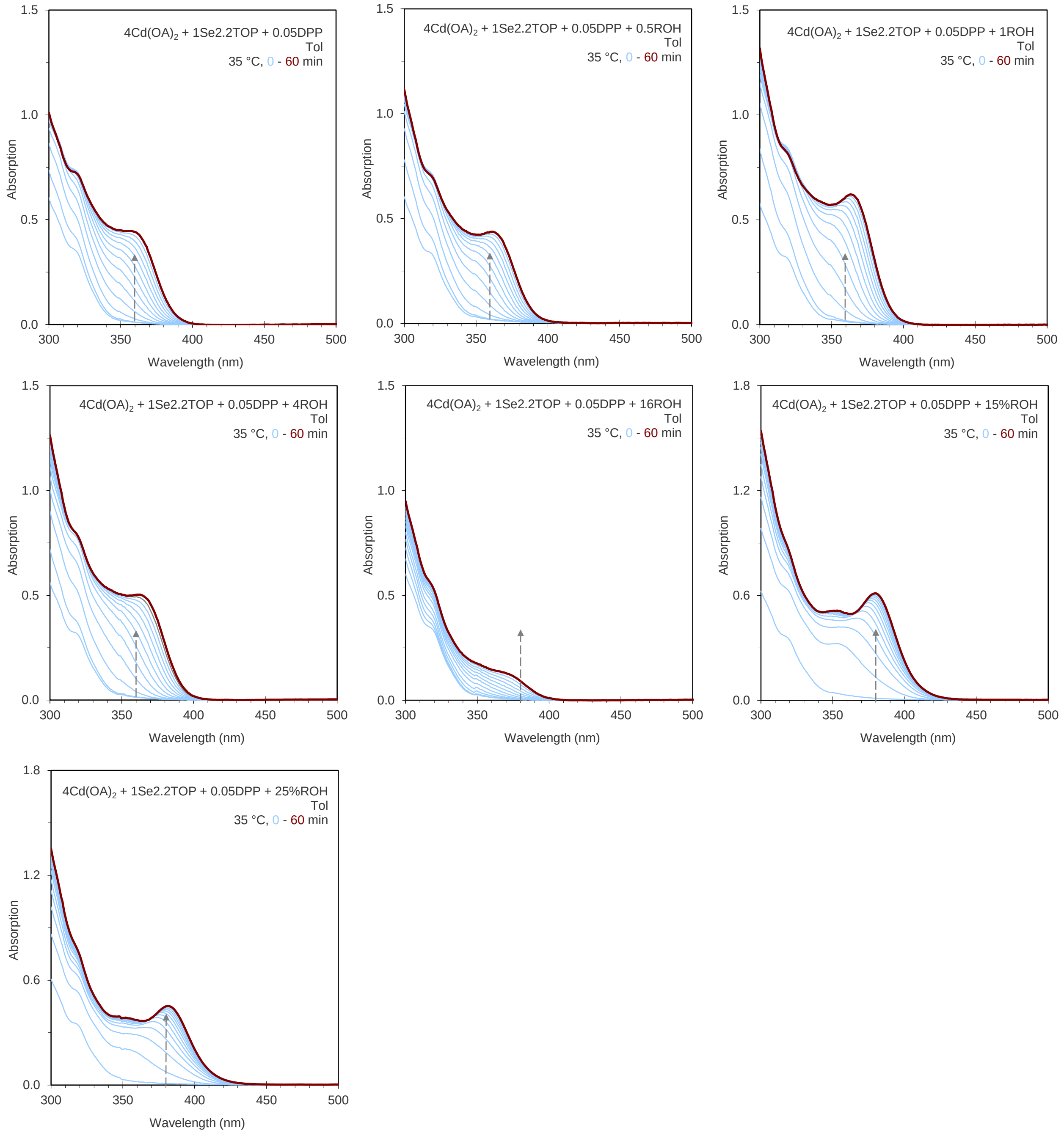

Supplementary Figure 7. The temporal evolution of absorption of growing CdSe NCs from the batches at 35 °C with the use of $0.05HPPh_2$ to illustrate the promotion or inhibition by the additive of $R_{12}H_{25}OH$ on nucleation/growth. The spectra were collected (by Cary 300 with an optical light path of 2 mm) from the 4Cd-to-1Se molar ratio batches with [Se] of 17 mmol kg^{-1} in ODE and a total weight of ~2.3 g. Commercial 90% TOP was used to make our 1M SeTOP.

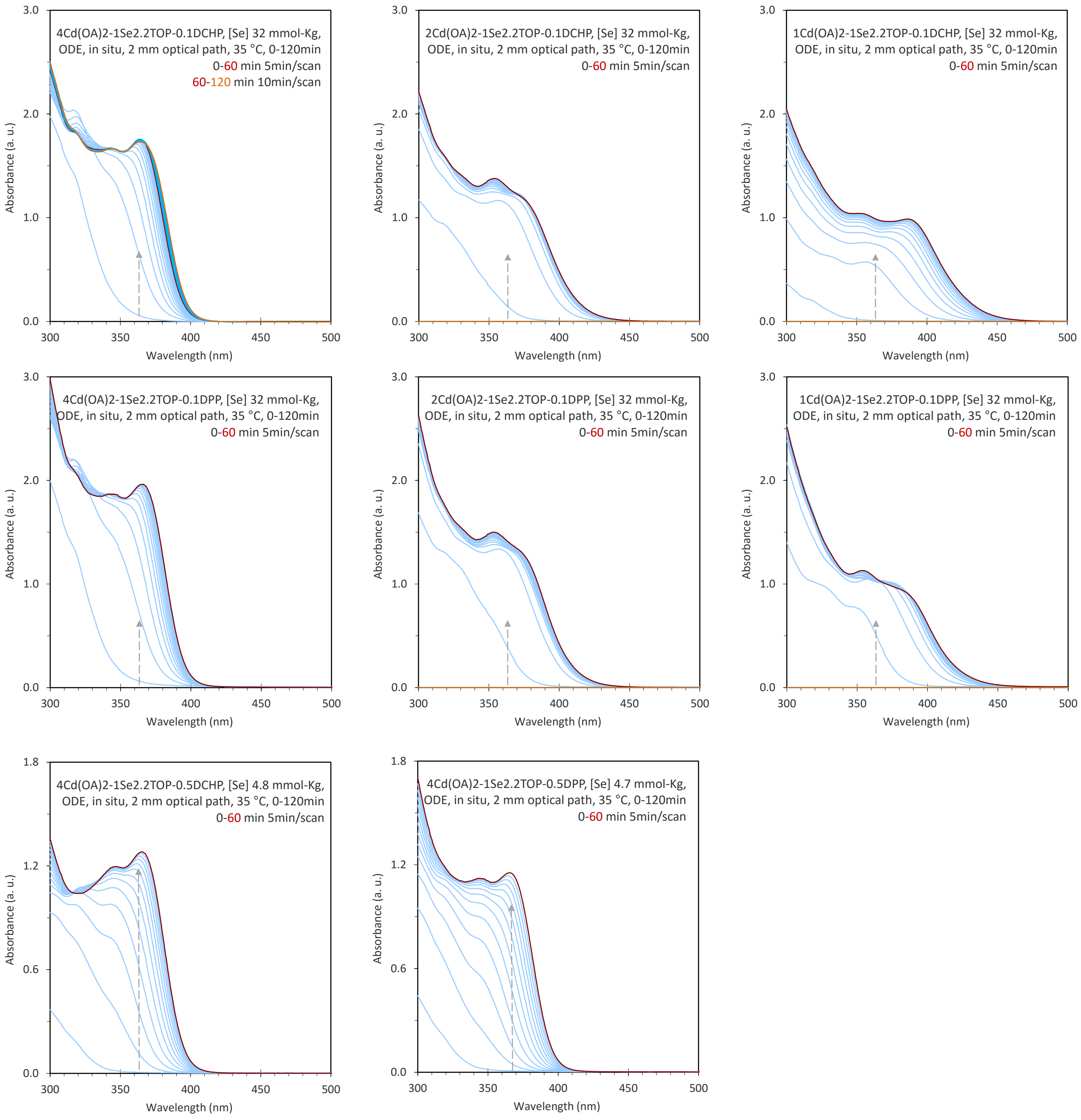

Supplementary Figure 8. The temporal evolution of absorption of growing CdSe NCs from the batches at 35 °C to illustrate effect of Cd-to-Se feed molar ratios on nucleation/growth.

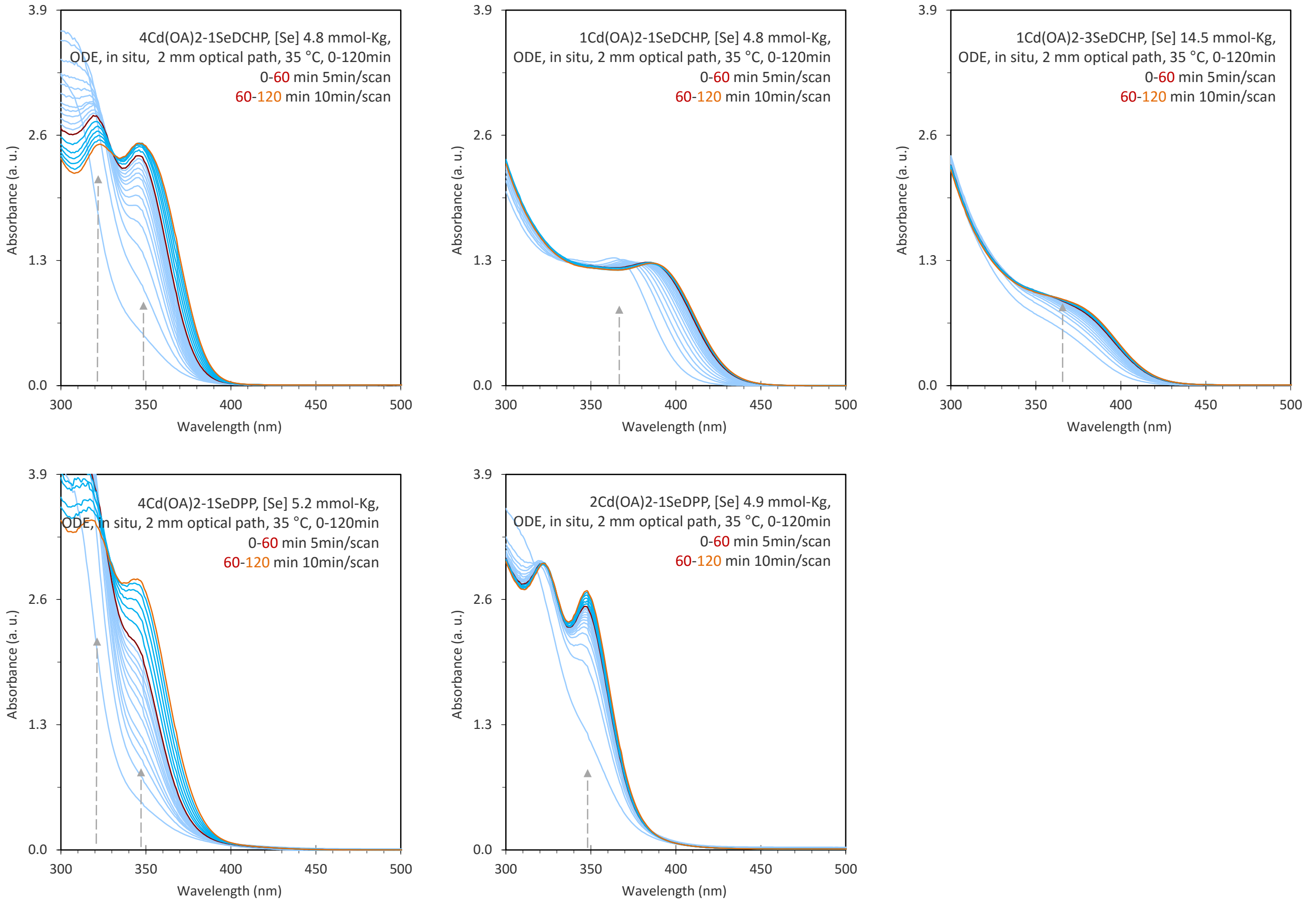

Supplementary Figure 9. The temporal evolution of absorption of growing CdSe NCs from the batches at 35 °C to illustrate effect of SeDPP and SeDCHP on nucleation/growth, together with the effect of Cd-to-Se feed molar ratios.

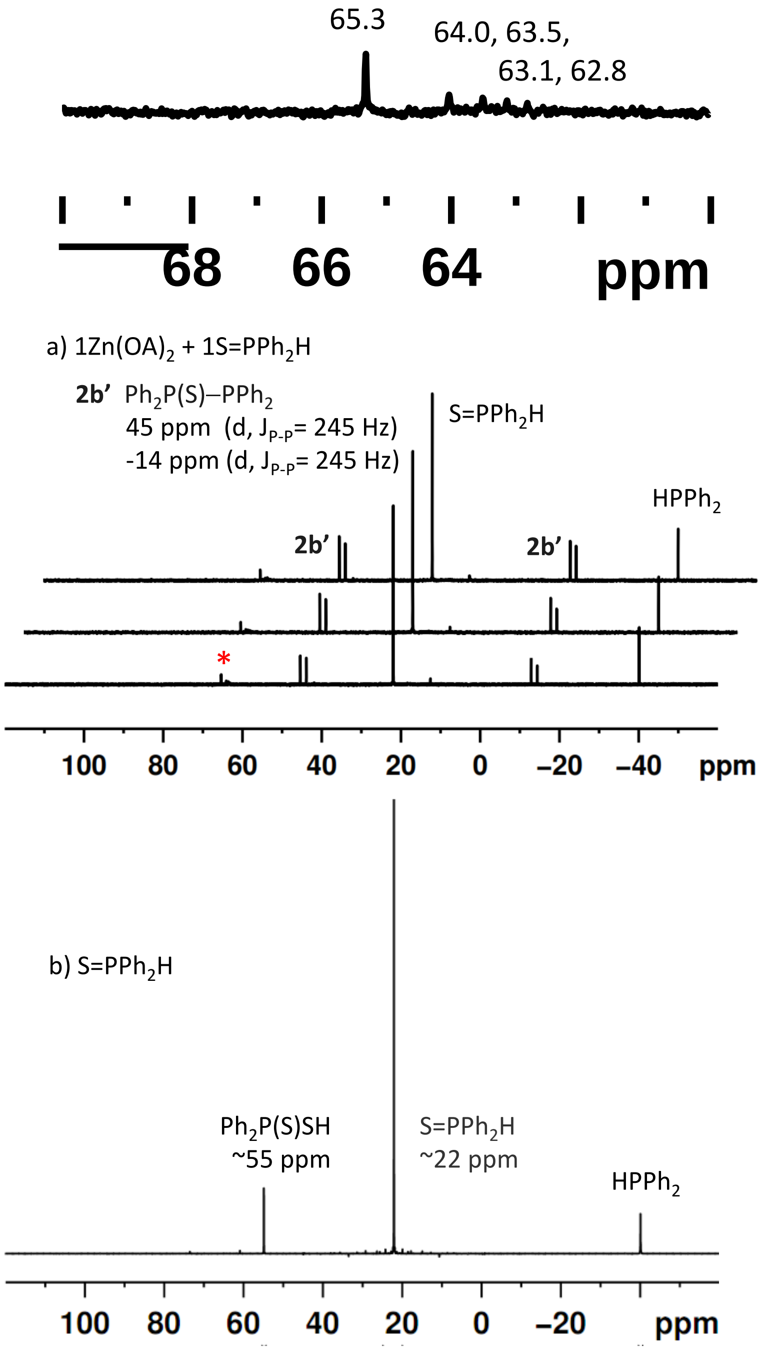

Supplementary Figure 10. ^{31}P NMR spectra collected from the mixtures indicated.

a) RT/15-30-60 min and [S] = 42 mmol/Kg. The red * is highlighted for the possible MS_2PPh_2 structure existing which we expect for our R group to be ~60 ppm. Note that Hendricks, M. P. *et al* documented $Cd(S_2PPh_2)_2$ 65.7 ppm, $Cd(S_2PPh_2)_2$(TMEDA) 64.01 ppm and [NHB](PP) 60.86 ppm[3]. McCleverty, J. A. *et al* discussed $Zn(S_2PPh_2)_2$ 62.8 ppm, $[NEt_4][Zn(S_2PPh_2)_3]$ 61.6 ppm and $[C_5H_5NH](S_2PPh_2)$ 61.3 ppm[4]. Goda, K. *et al* reported the synthesis of $Ph_2P(S)$–SR with ^{31}P NMR of R = CH_3, no ^{31}P data, R= -CH_2-CH=CH_2, 62.4 ppm, R= -CH_2Ph, 62.9 ppm and R=Ph, 64.8 ppm[5]. Al-Shboul, T. M. A. *et al* addressed ^{31}P NMR of E_2PPh_2 around ~62 ppm[6].

b) The prepared S=PPh_2H contains free $HPPh_2$.

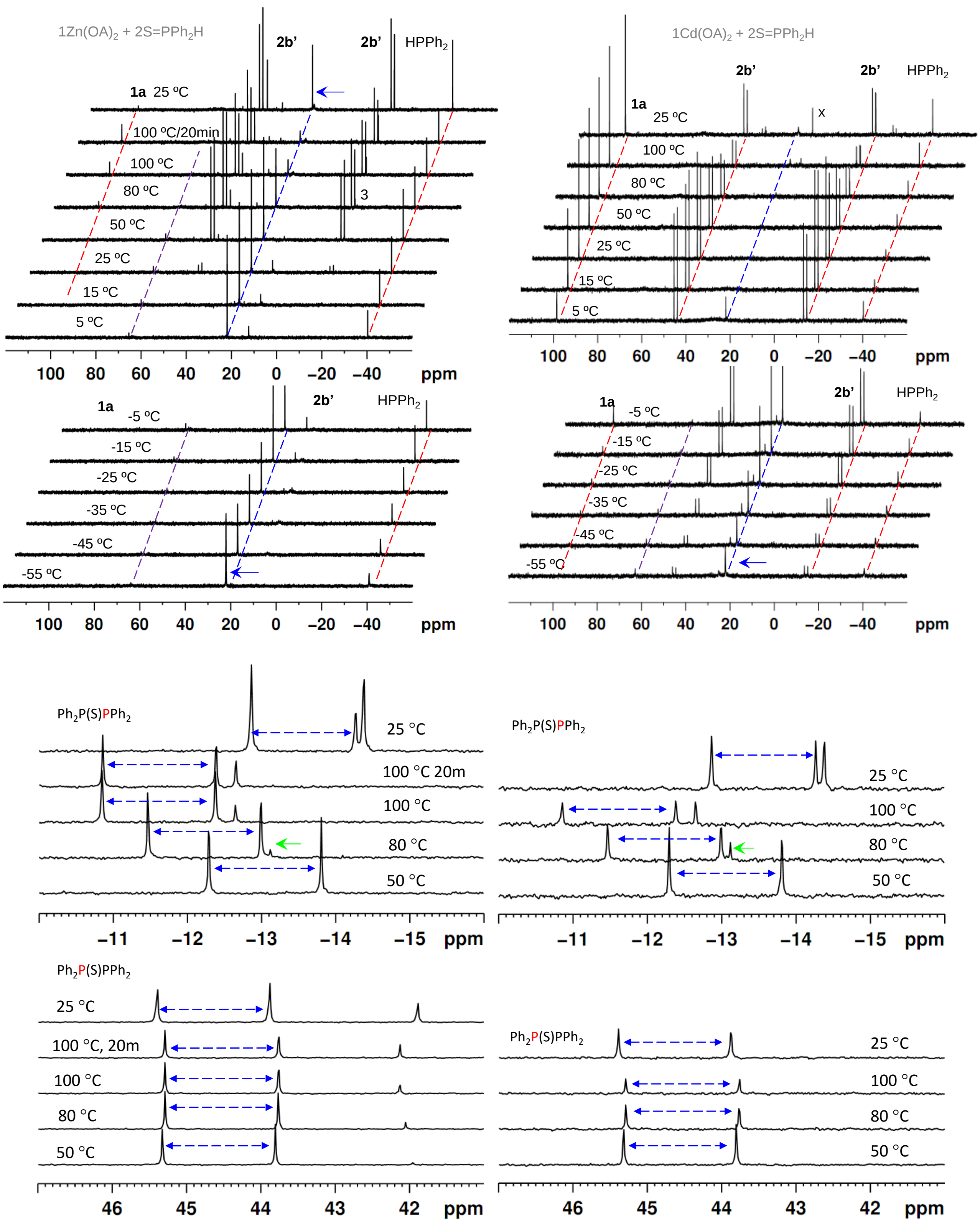

Supplementary Figure 11. ^{31}P NMR spectra collected from the two mixtures (from -55 °C to 100 °C and back to 25 °C, left Zn = 42 mmol/Kg and right Cd = 48 mmol/Kg) indicated for the study of **2b'** and **1b**. For both the reactions, **1b** came up around 80 °C.

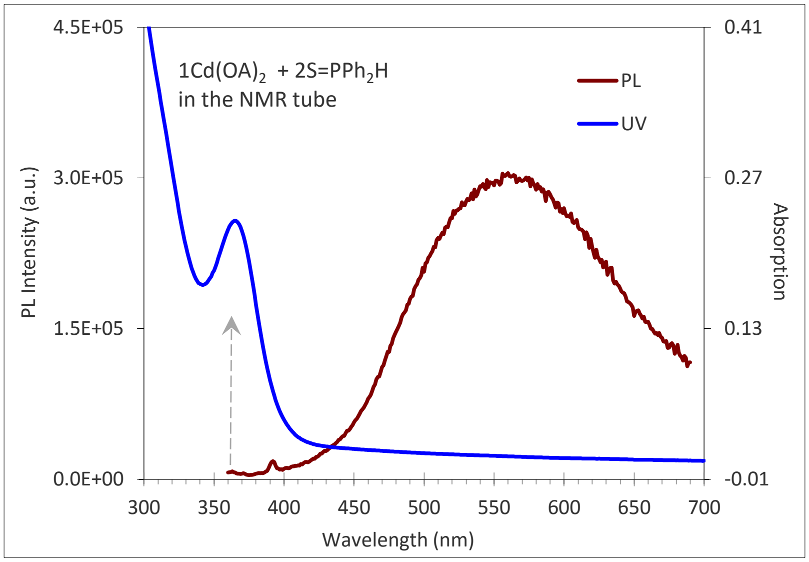

Supplementary Figure 12. Optical properties of Batch Supplementary Fig. 11 (Cd = 48 mmol/Kg) (5 uL/1mL toluene; excitation wavelength is 350 nm).

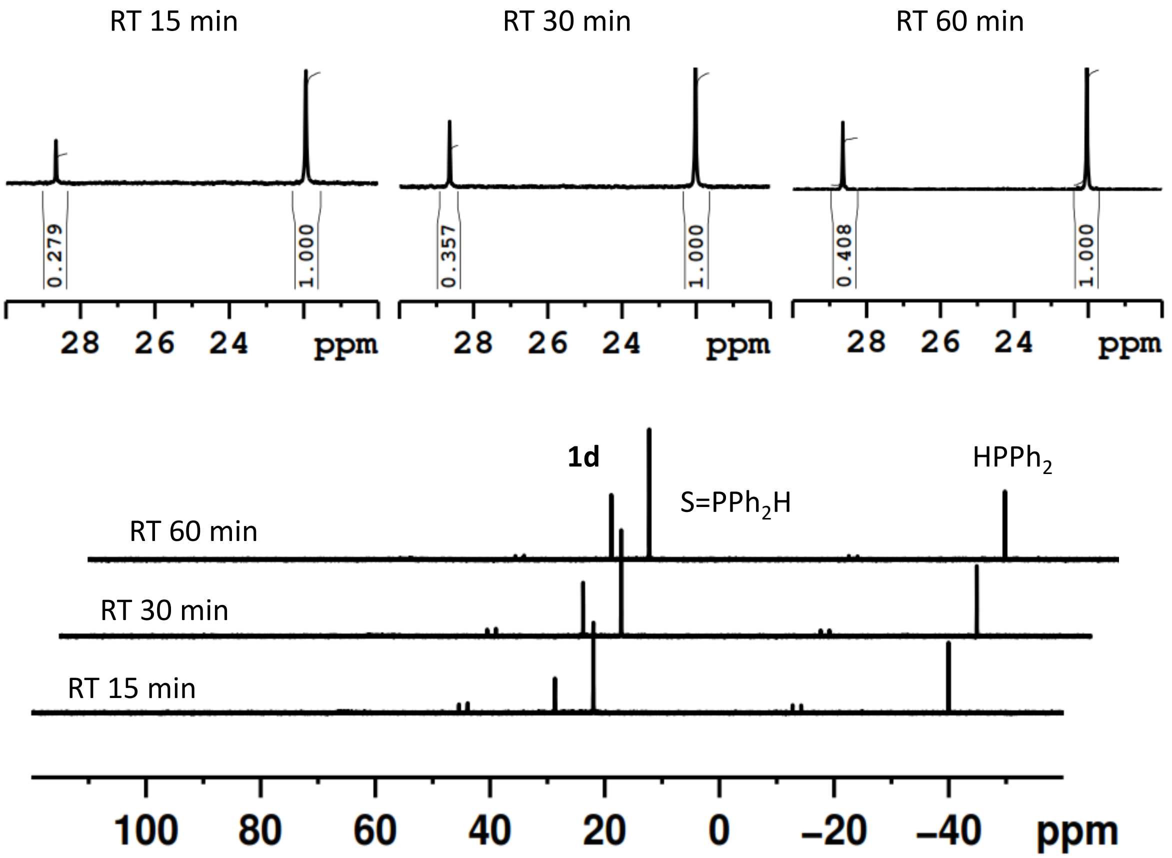

Supplementary Figure 13. The **1d**–to–S=PPh$_2$H ratio increased from 0.28 (15 min), 0.36 (30 min), to 0.41 (60 min) is demonstrated for the reaction of 2Cd(OOCC$_{17}$H$_{33}$)$_2$ + 1S=PPh$_2$H + 4C$_{12}$H$_{25}$SH shown by Fig. 2**c** (in our main text). Here, we used D1=2 seconds (64 scans total taking ~3 mins). To explore a reaction at one temperature and at different reaction times, the disappearance of a reactant (R), the increase of a product (P), or the increase of the ratio of P/R could be meaningful. Thus, to judge the consumption of one starting precursor (S=PPh$_2$H) when it is still present along a reaction such as Fig. 2**c**, we have always been paying attention to the product-to-reactant ratio of **1d**-to-S=PPh$_2$H, which obviously increased, indicating the formation of **1d** and the consumption of S=PPh$_2$H. Regarding our qualitative and not quantitative ^{31}P NMR, it is meaningful to consider the reaction progress by comparing the product-to-reactant ratio, although ^{31}P NMR quantitation is not as accurate as ^{1}H NMR. Critically, when we set the integral of the 15 min spectrum S=PPh$_2$H to be 1, we obtained the integral of 1.00 (15 min), 1.06 (30 min), and 1.07 (60 min); the S=PPh$_2$H signal seems to become narrower along the progress of the reaction.

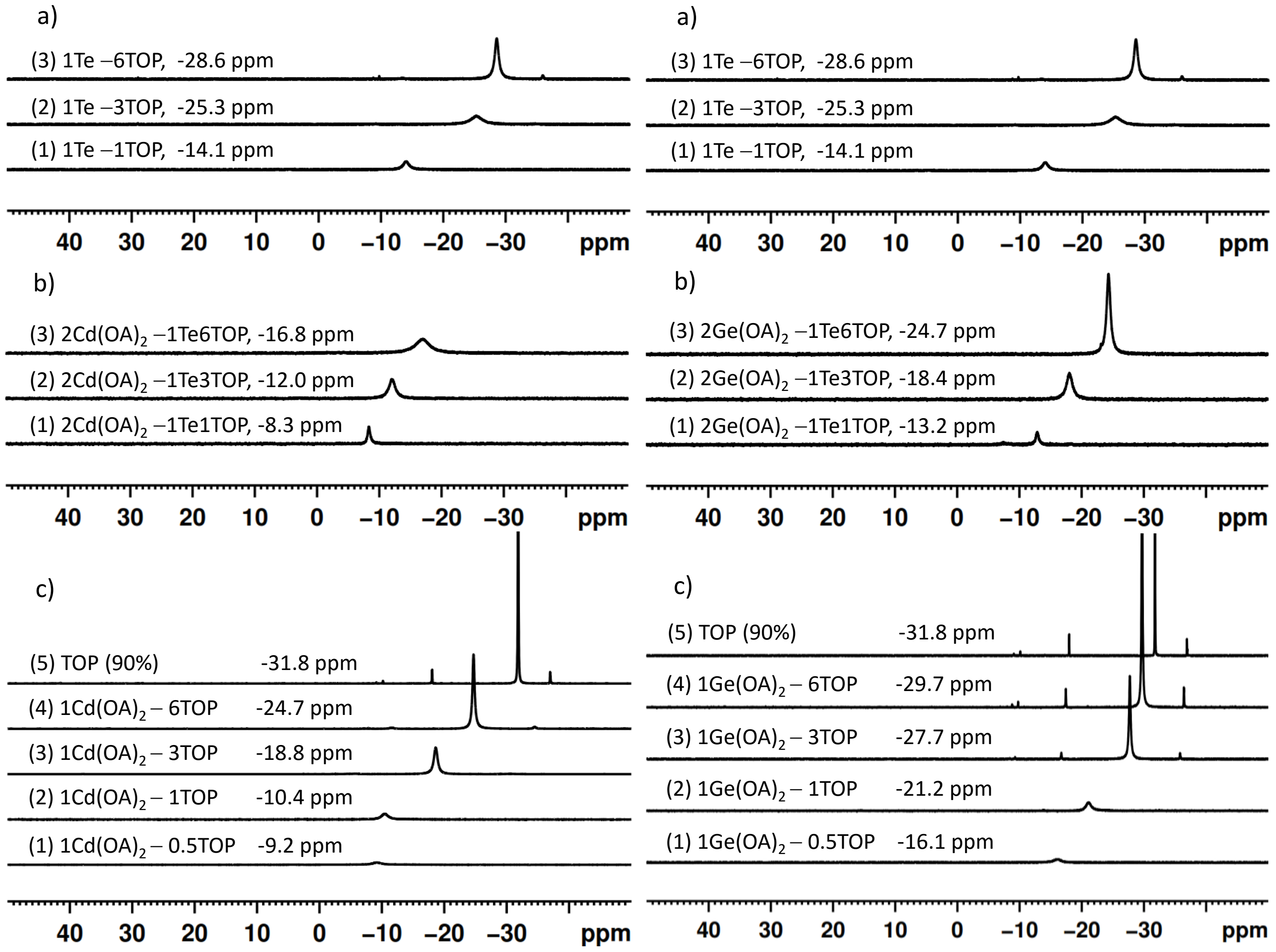

Supplementary Figure 14. ^{31}P NMR spectra collected from the mixtures indicated. The Te-TOP coordination is indicated in (a, both left and right). It is clear that Cd-TeTOP coordination is (left-b) stronger than Ge-TeTOP coordination (right-b). Also, Cd-TOP coordination (left-c) is stronger than Ge-TOP coordination (right-c).

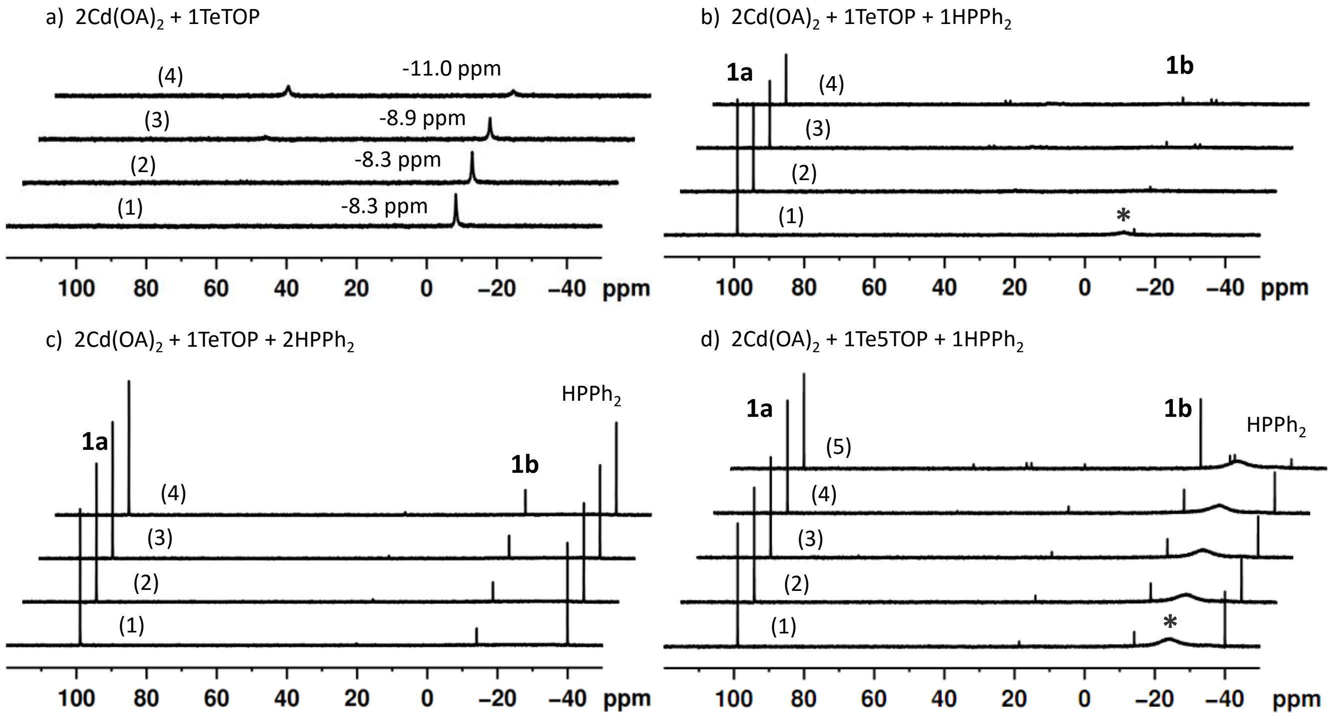

Supplementary Figure 15. ^{31}P NMR spectra collected from the mixtures indicated. [Te] = 20 mmol/Kg. The Cd-related coordination is indicated by *.

a) 15 min at RT-80 °C-120 °C-160 °C.
b) RT/15min, 80 °C/15-30-45 min.
c) RT/15-30-45-60 min.
d) RT/15-30-45-60-1080 min. The evident TOP coordinated to $Cd(OA)_2$ could result in low precursor reactive as well as the equilibrium of TeTOP + $HPPh_2$ $\Leftrightarrow$ TOP + Te=PPh_2H to the left.

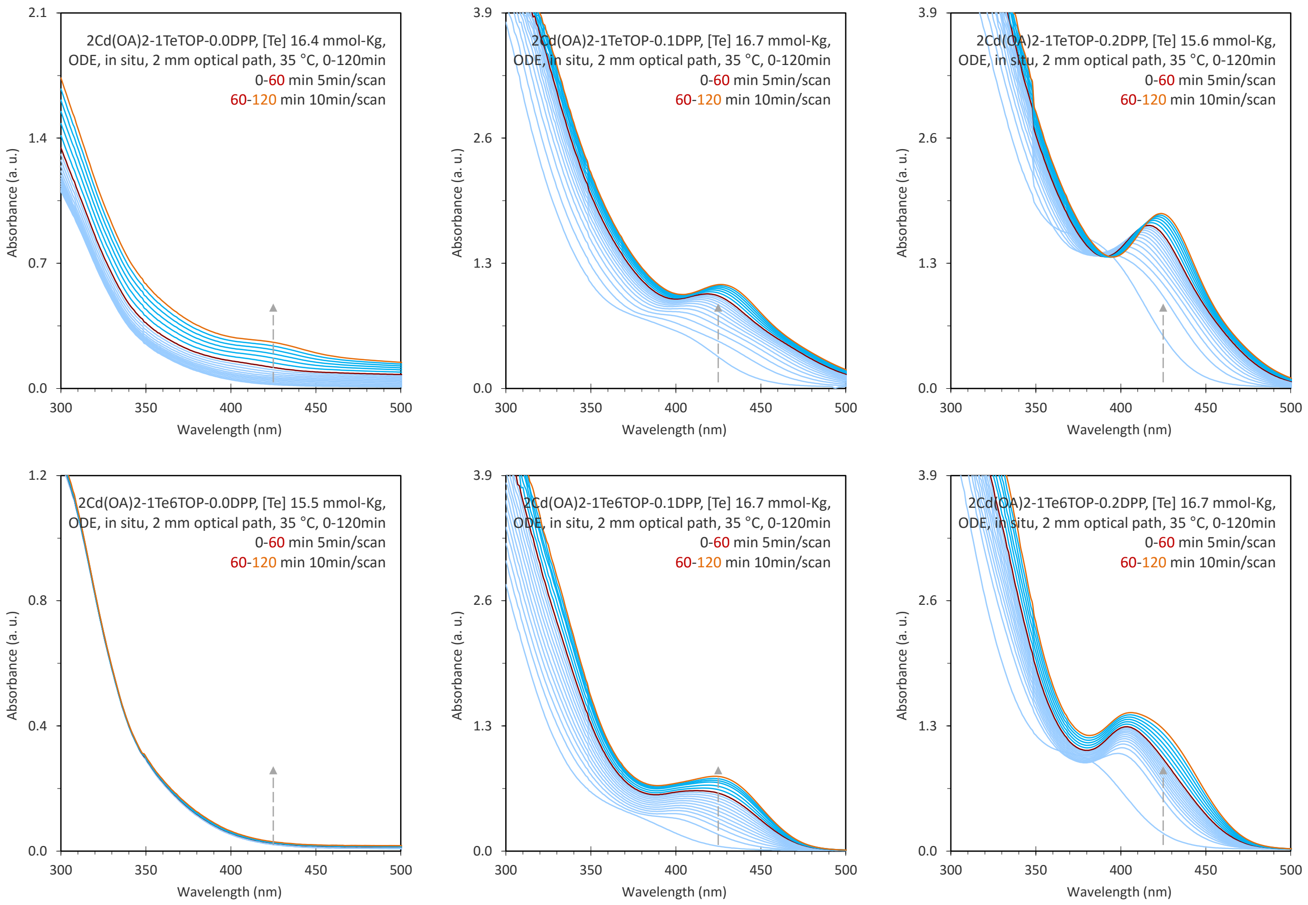

Supplementary Figure 16. In-situ absorption study of the batches indicated. The use of $HPPh_2$ can promote the precursor reactivity. Clearly, the growth of the CdTe NCs is also affected by the amount of TOP and $HPPh_2$ used. The particle numbers obtained in the top panel with less TOP are much more than those in the bottom panel with more TOP. Therefore, the use of $HPPh_2$ combined with limited amount of TOP (via high M-to-E and low E-to-TOP feed molar ratios) is practical to synthesize NCs at low temperature with high particle yield.

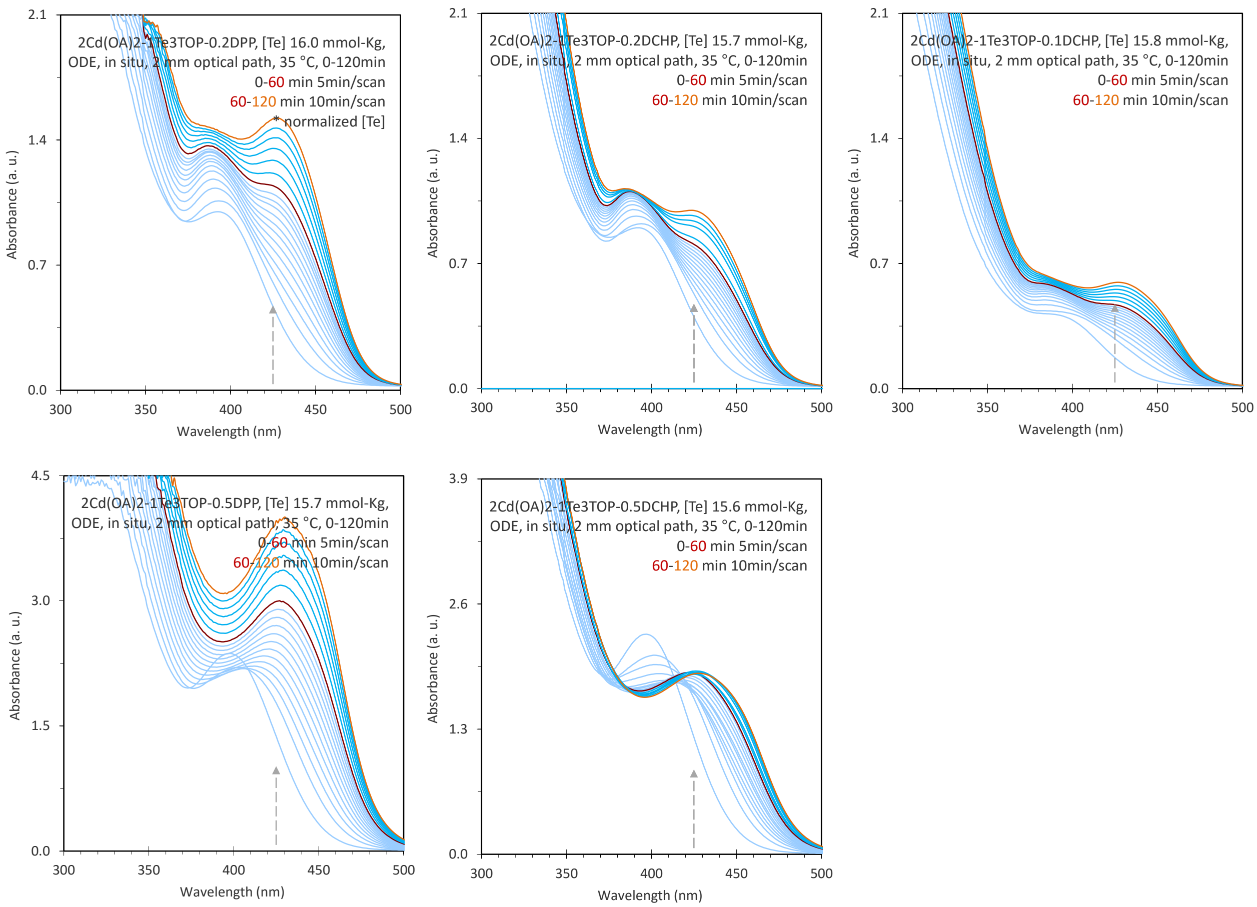

Supplementary Figure 17. In-situ absorption study of the batches indicated. The use of DCHP and $HPPh_2$ is compared. Clearly, the growth of the CdTe NCs is also affected by the nature of the secondary phosphine used.

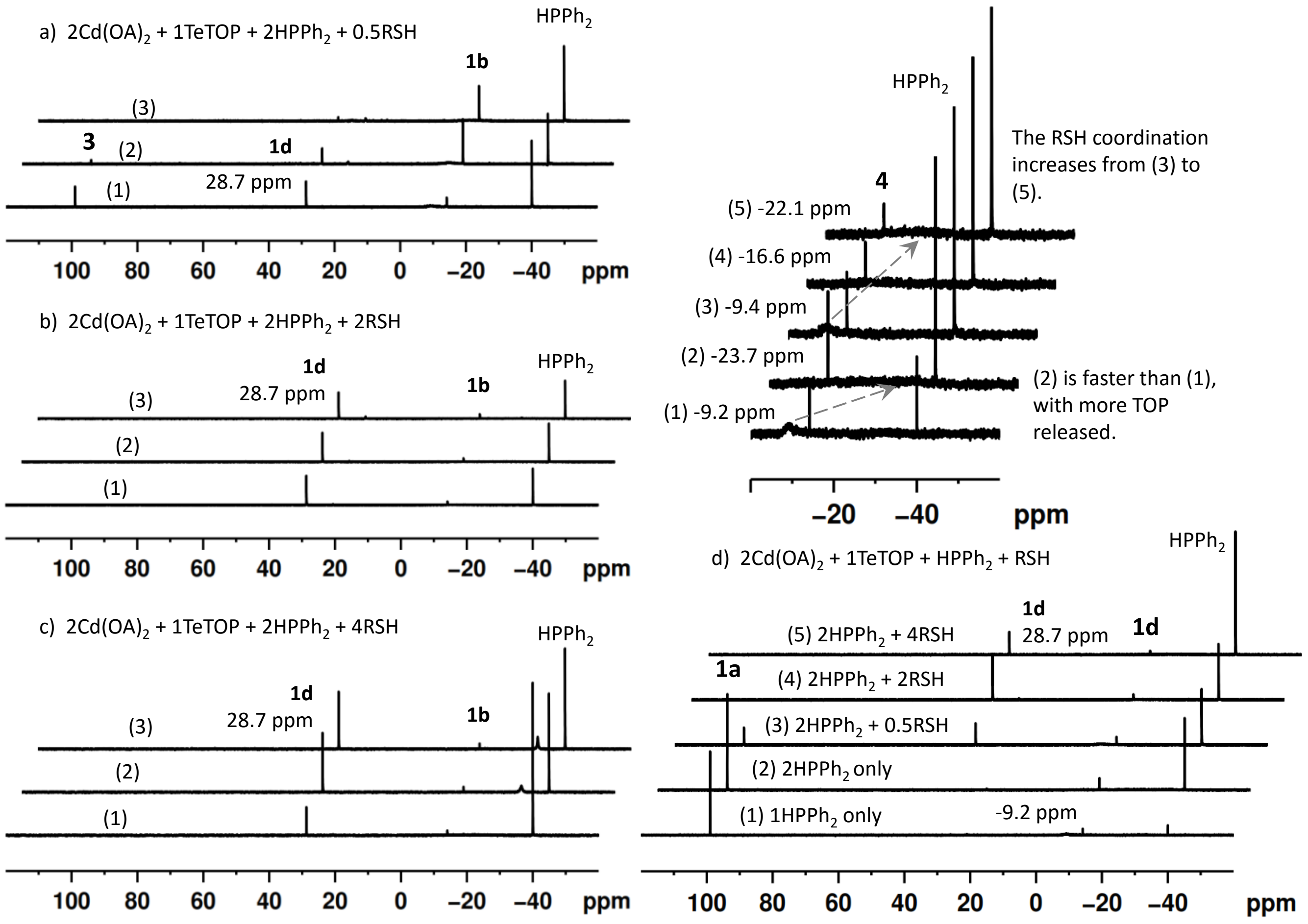

Supplementary Figure 18. ^{31}P NMR spectra collected from the mixtures indicated. [Te] = 20 mmol/Kg. The amounts of $HPPh_2$ and $C_{12}H_{25}SH$ affect the kinetics of the reactions. a-c) 30 min at RT-80 °C-120 °C. d) RT/30 min.

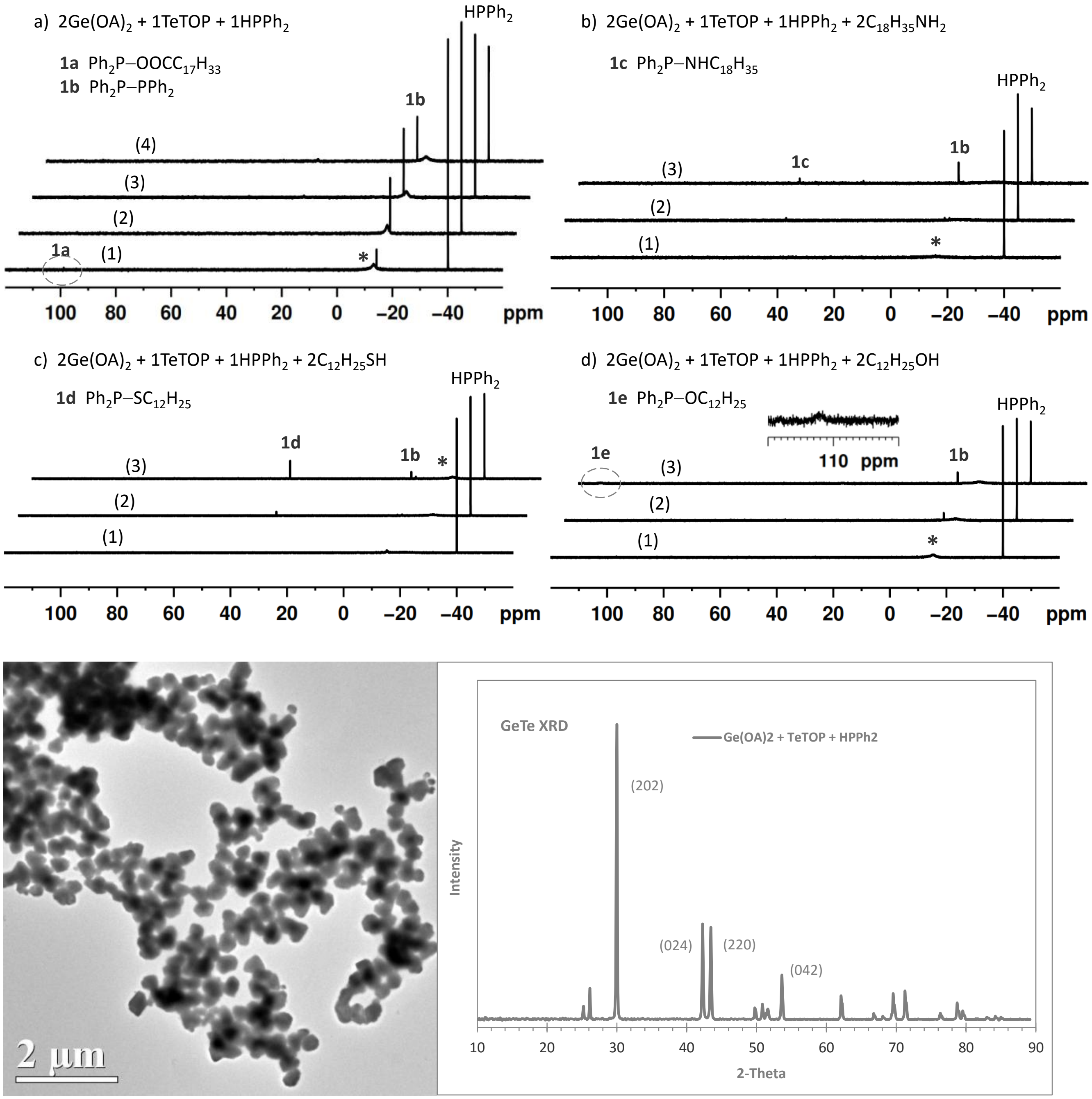

Supplementary Figure 19. (Top) ^{31}P NMR spectra collected from the mixtures indicated. [Te] = 15 mmol/Kg. The Ge-related coordination is indicated by *. a) 30 min at RT-50 °C-80 °C-100 °C. b-d) 30min at RT-80 °C-120 °C. (Bottom). TEM (left) and XRD (right) of GeTe nanocrystals prepared.

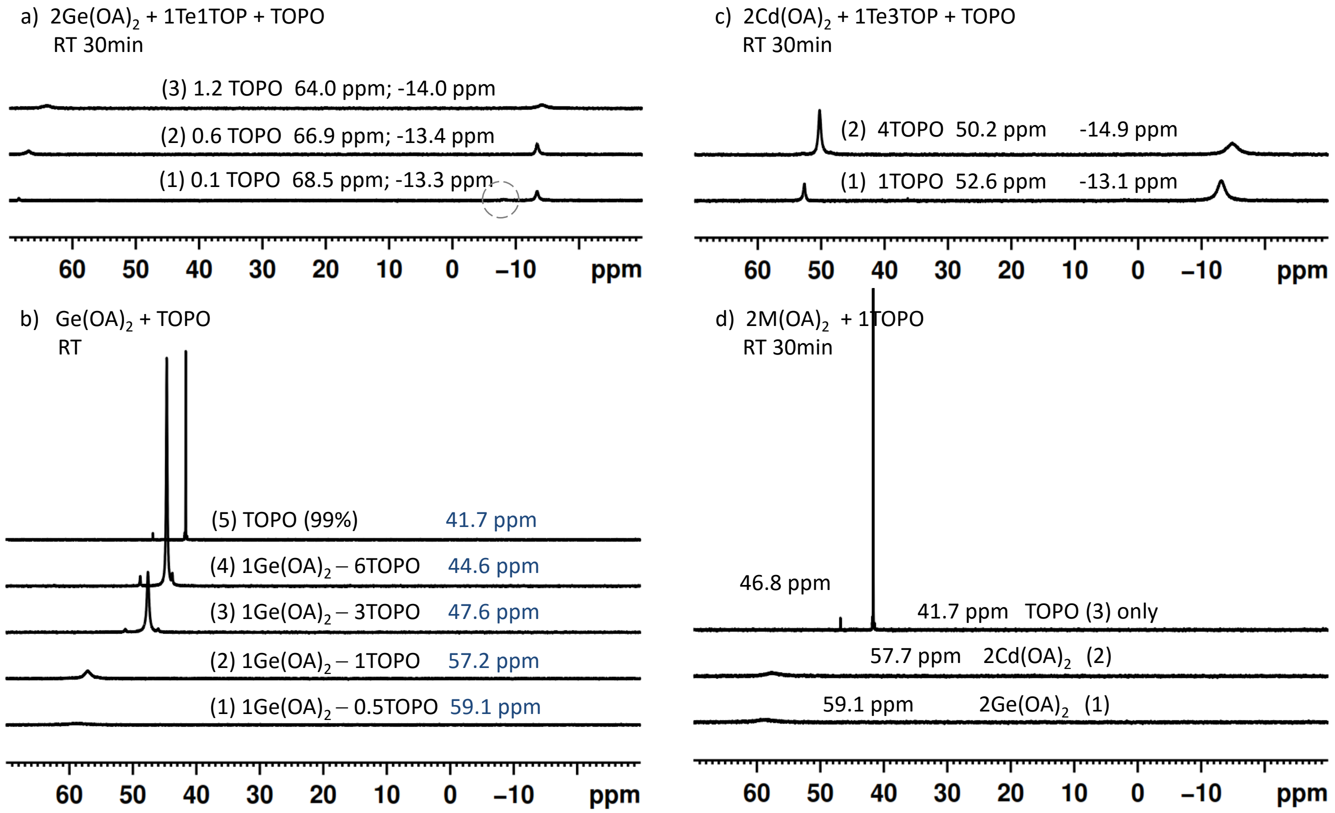

Supplementary Figure 20. ^{31}P NMR spectra collected from the mixtures indicated. It is clear that Cd-TOPO coordination is weaker than Ge-TOPO coordination. TOPO ^{31}P NMR chemical shift has been documented[7,8].

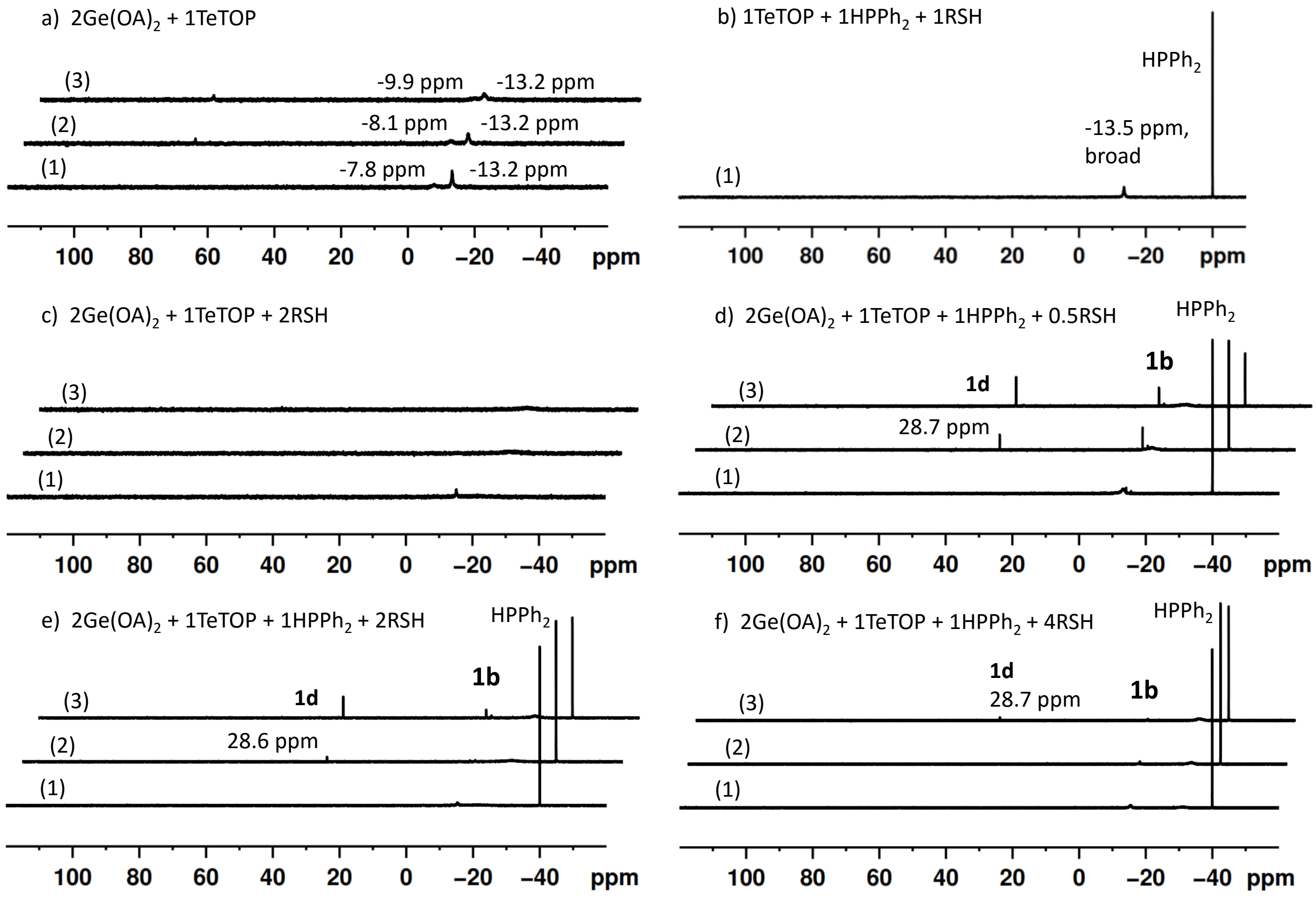

Supplementary Figure 21. ^{31}P NMR spectra collected from the mixtures indicated. [Te] = 20 mmol/Kg. a, c-f) 30 min at RT-80 °C-120 °C. b) RT/10 min.

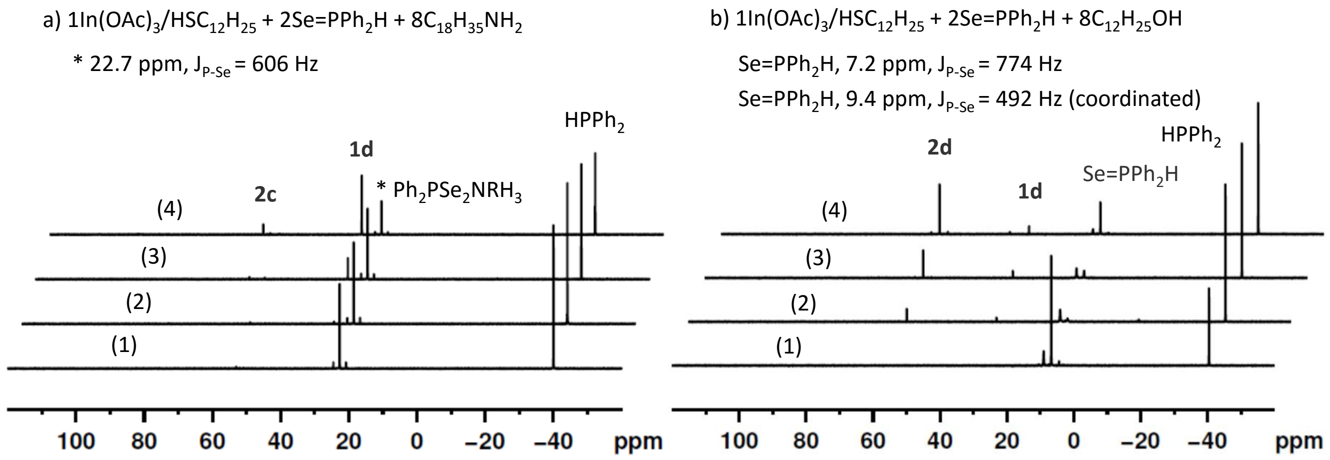

Supplementary Figure 22. ^{31}P NMR spectra collected from the mixtures indicated. [Se] = ~40 mmol/Kg. a-b) 10 min at RT-60 °C-80 °C-100 °C. The synthesis of * in a) can also be found[9]. It is helpful to mention the paper “Coordination chemistry of diselenophosphinate complexes: the X-ray single-crystal structures of $[K(Se_2PPh_2)(THF)_2]_2$ and $[In(Se_2PPh_2)_3]$•L (L = THF, PhMe)”[10].

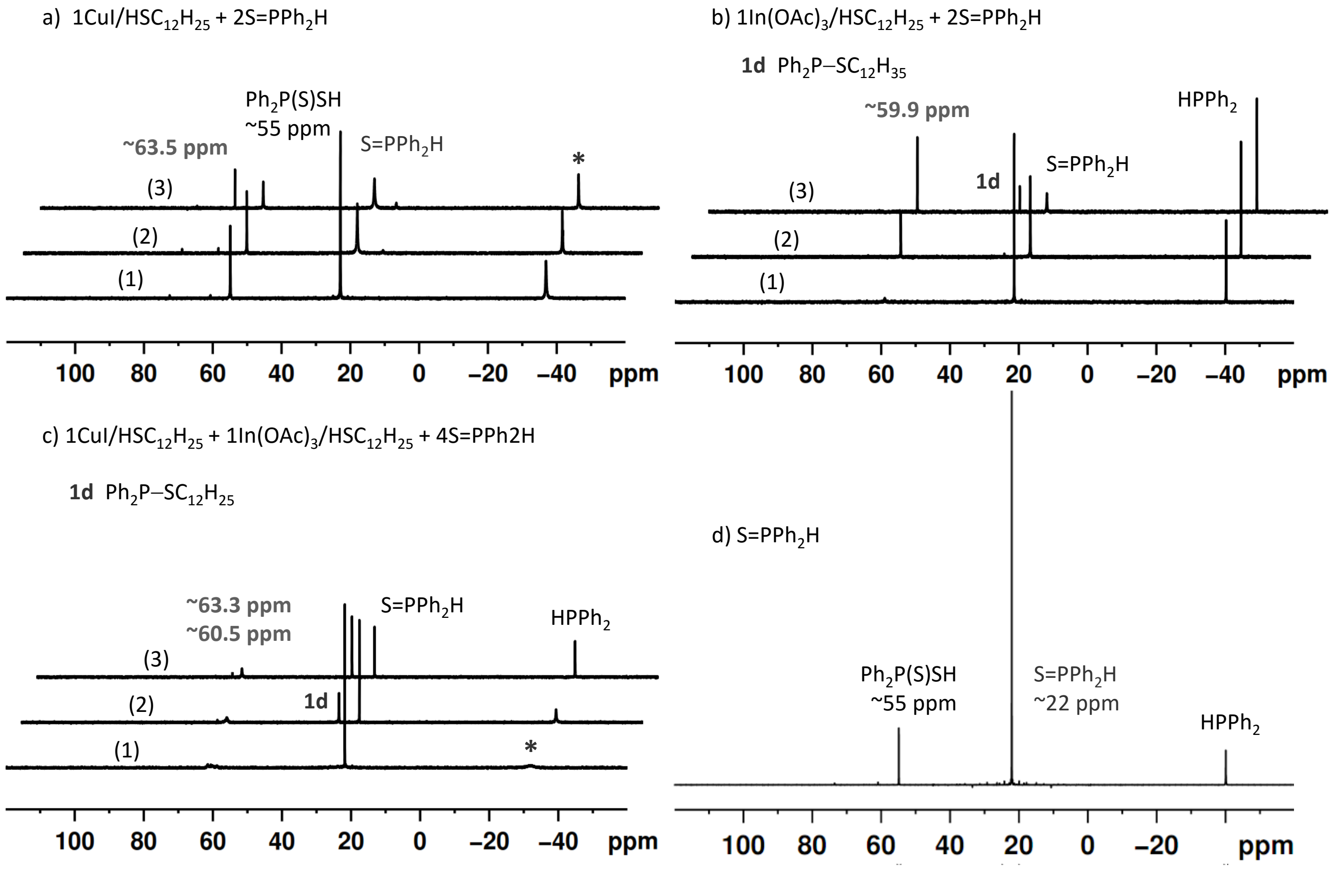

Supplementary Figure 23. ^{31}P NMR spectra collected from the mixtures indicated. [S] = 80 mmol/Kg. a-c) RT/10 min-80 °C/10 min-100 °C/10 min. Again, the peak 60-65 ppm could be $M(S_2PPh_2)$ (M = Cu or In or Zn (Supplementary Fig. 2)) type intermediates and * indicates $HPPh_2$[11] coordinated to Cu[12-15].

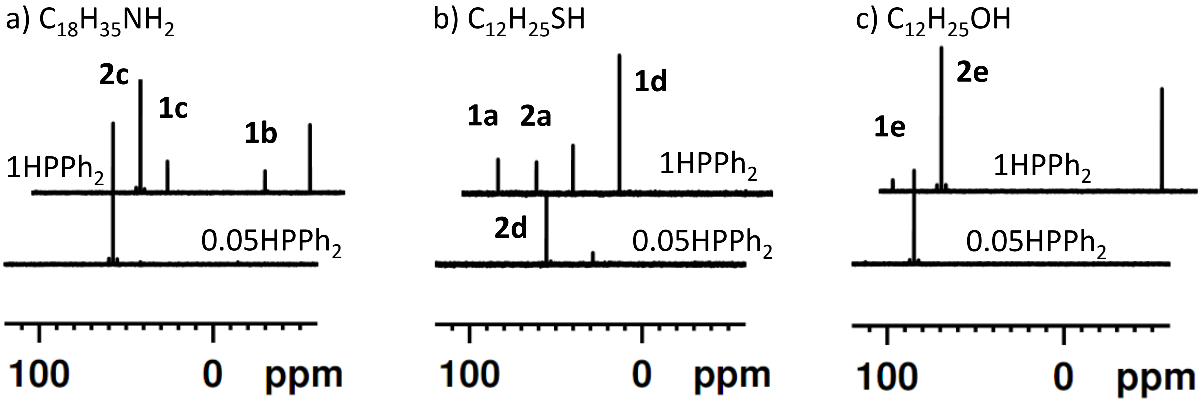

Supplementary Figure 24. ^{31}P NMR monitoring of the formation of **1** (Ph_2P–Y) and/or **2** ($Ph_2P(Se)$–Y) as main species when HY and $HPPh_2$ were added to the mixture of Compound **3** + $6Cd(OA)_2$ (as mentioned in Fig. 5, at RT after 10 min (0.05 $HPPh_2$) or 10 min (1$HPPh_2$)). **a**, $16RNH_2$. **b**, 4RSH. **c**, 16ROH.

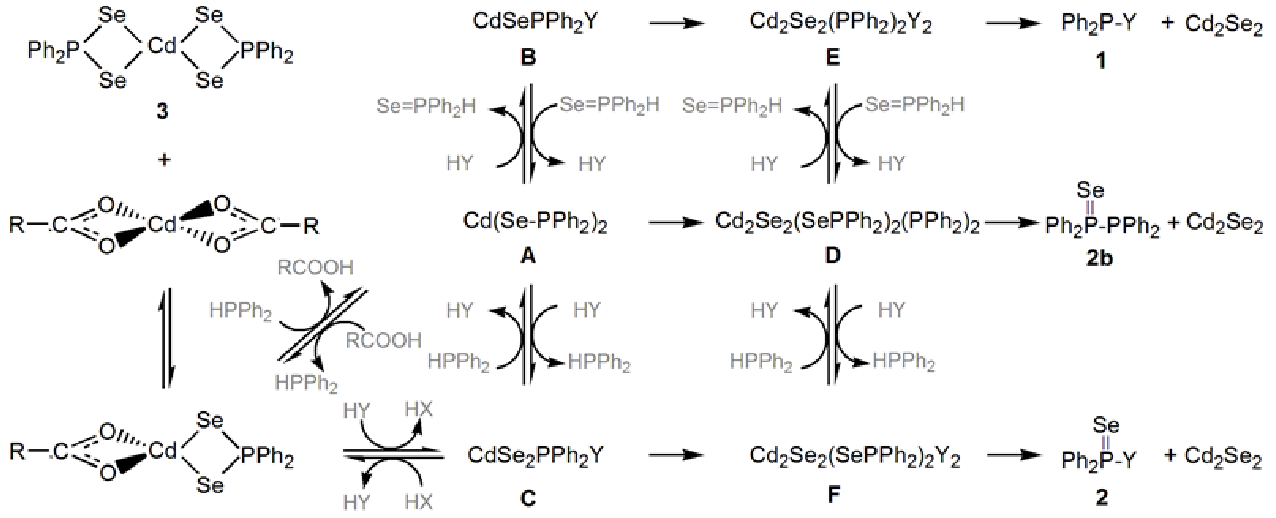

Supplementary Figure 25. Schematic drawing of Pathway 2 (from $RCOOCdSe_2PPh_2$ to **C** to **F** to **2**) involved in Fig. 5 without the presence of $HPPh_2$, and of Pathway 1 (from $RCOOCdSe_2PPh_2$ to **A** to (**B** or **D**) to **E** to **1**) with the presence of a certain amount of $HPPh_2$ involved in Supplementary Fig. 24.

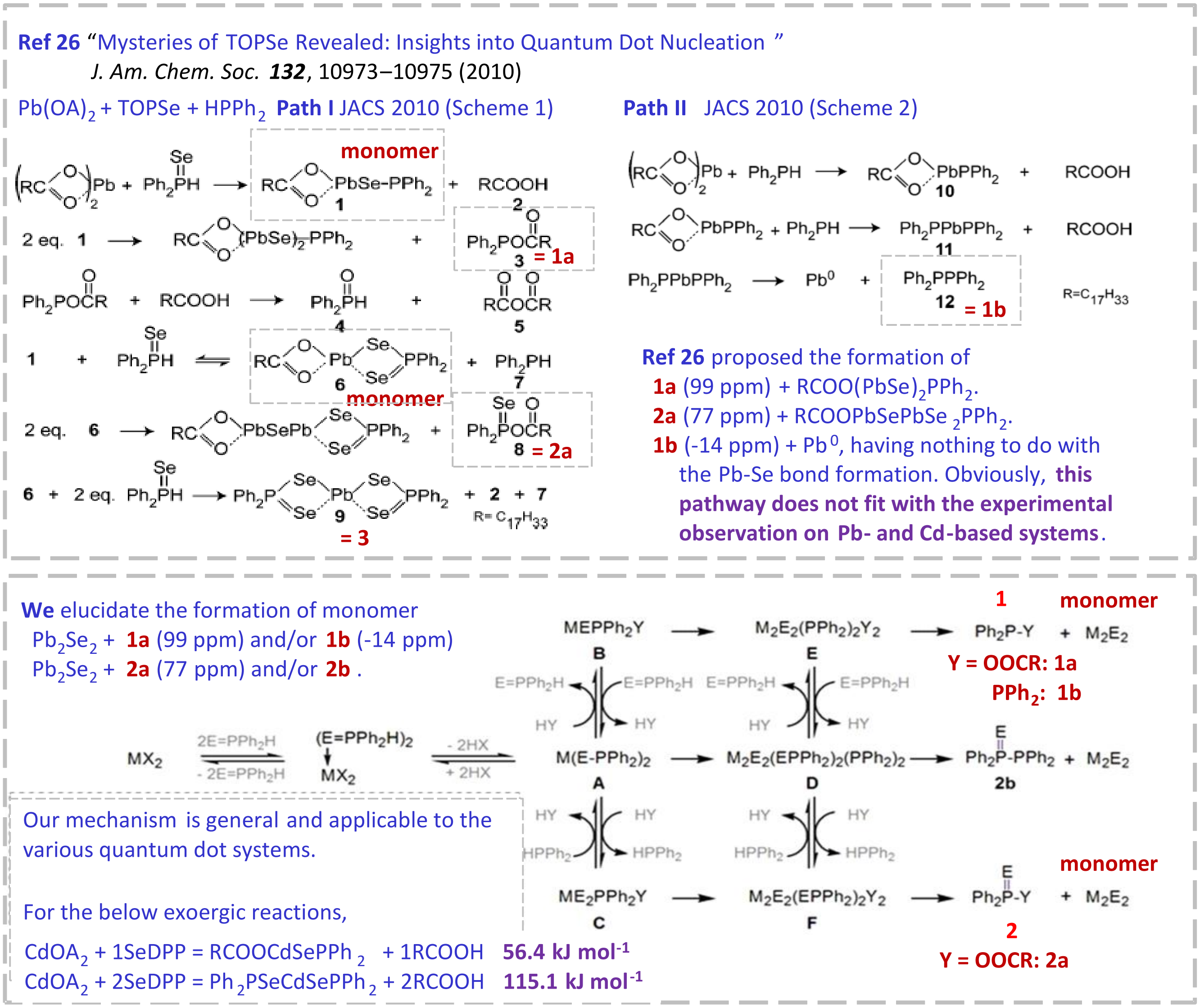

Supplementary Figure 26. The mechanistic difference between the two studies, Ref 26 (JACS 2010) and the present investigation. Top is from JACS 2010 (with 3 = **1a**, 8 = **2a**, 12 = **1b** 9 = **3** and labeled) and bottom from our Fig. 1. Clearly, with Y = OOCR or PPh_2, the two studies detected the same P-containing chemicals **1a** (99 ppm, Ph_2P–OOCR), **2a** (77 ppm, $Ph_2P(Se)$–OOCR) and **1b** (–14 ppm, $Ph_2P–PPh_2$) by ^{31}P NMR. However, our important IM **A** ($Pb(SePPh_2)_2$ with M = Pb and E = Se) and its dimer **D**, together with their reactions with H–Y leading to IM **E** and **F** were not mentioned in the JACS 2010 study.

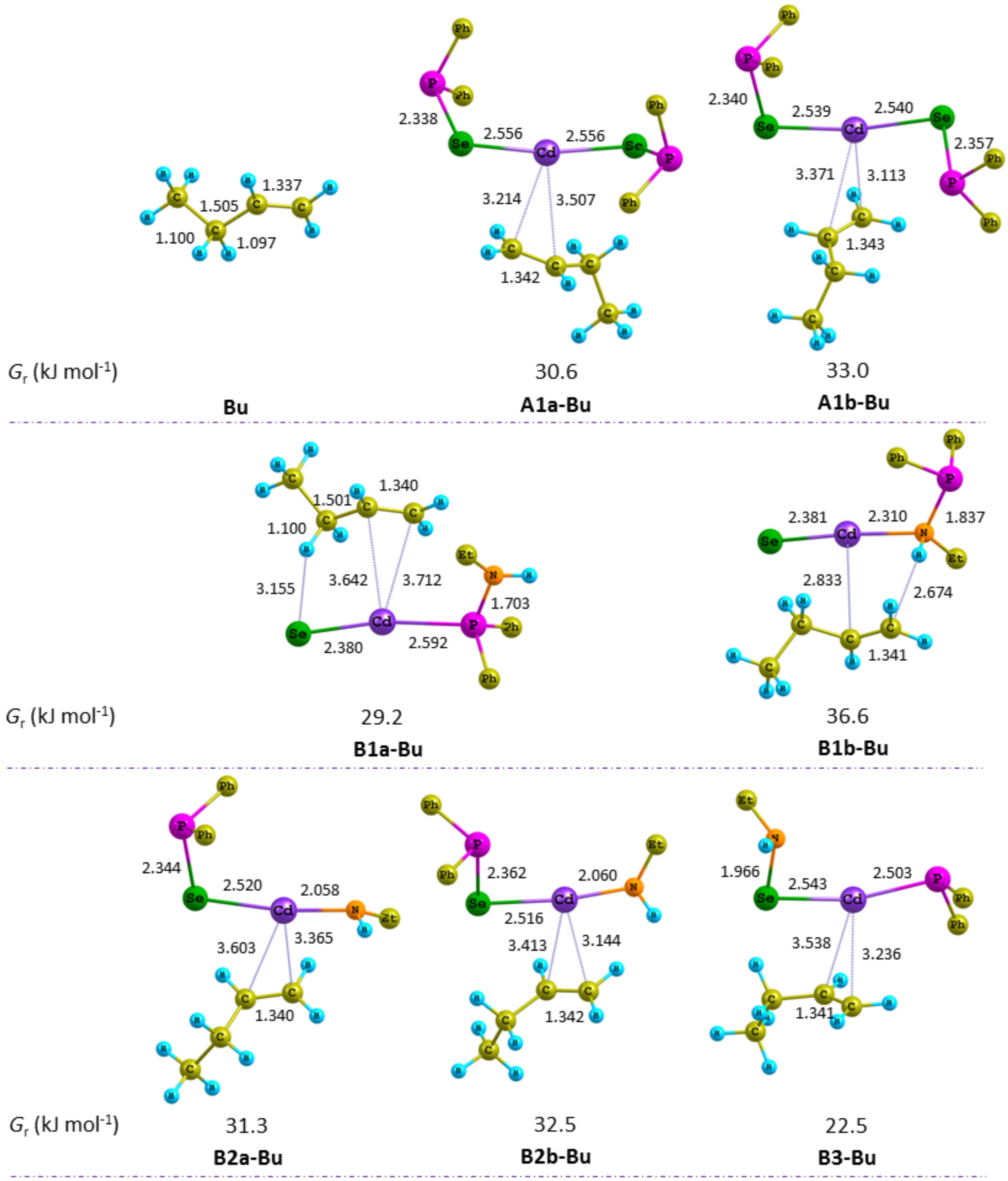

Supplementary Figure 27. The geometric structures of the intermediates indicated, and the relative Gibbs free energies (G_r) (kJ mol^{-1}) relative to the discrete reactants calculated at the M06//B3LYP/6-31++G(d, p), SDD level are shown. Bond lengths are reported in Å.

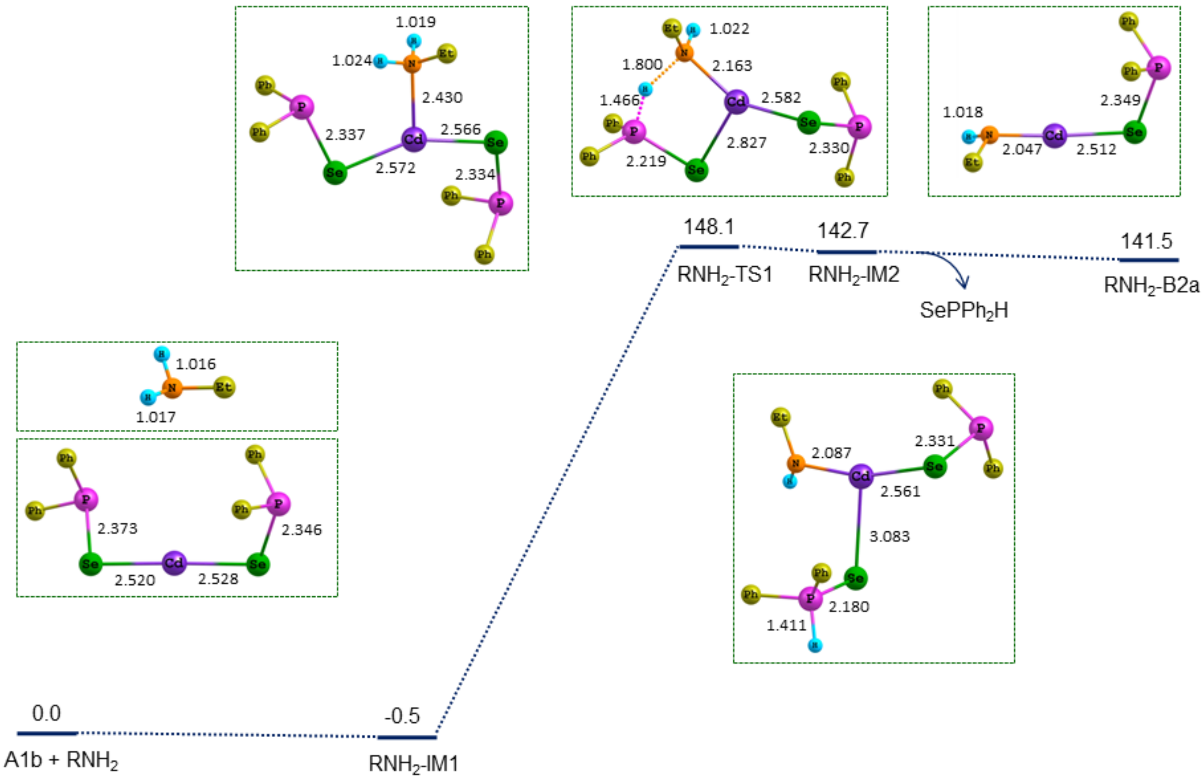

Supplementary Figure 28. The geometric structures of the species indicated and the schematic energy diagrams for the reaction of Ph_2PSe–Cd–$SePPh_2$ (**A1b**) + RNH_2 → RN–CdSe–PPh_2 (**RNH_2-B2a**) + $SePPh_2H$ calculated at the M06//B3LYP/6-31++G(d, p), SDD level are shown. Bond lengths are reported in Å. Relative Gibbs free energies (G_r) (kJ mol^{-1}) for the corresponding species are shown relative to **A1b** + RNH_2. For the additive RNH_2, the activation energy barriers for the N–H bond cleavage is 148.1 kJ mol^{-1}, resulting in the release of $SePPh_2H$ from **A1b** to **B2a**.

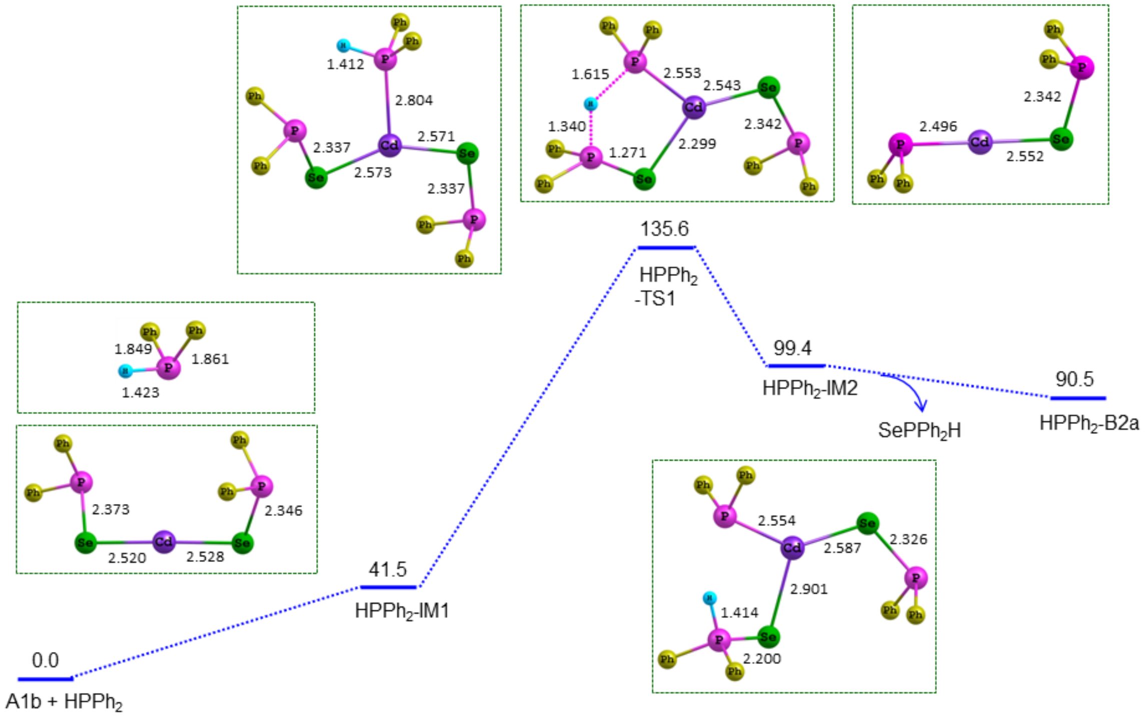

Supplementary Figure 29. The geometric structures of the species indicated and the schematic energy diagrams for the reaction of $Ph_2PSe–Cd–SePPh_2$ (**A1b**) + $HPPh_2$ → $Ph_2P–CdSe–PPh_2$ (**$HPPh_2$-B2a**) + $SePPh_2H$ calculated at the M06//B3LYP/6-31++G(d, p), SDD level are shown. Bond lengths are reported in Å. Relative Gibbs free energies (G_r) (kJ mol^{-1}) for the corresponding species are shown relative to **A1b** + $HPPh_2$. For the additive $HPPh_2$, the activation energy barriers for the P–H bond cleavage is 135.6 kJ mol^{-1}, resulting in the release of $SePPh_2H$ from **A1b** to **B2a**.

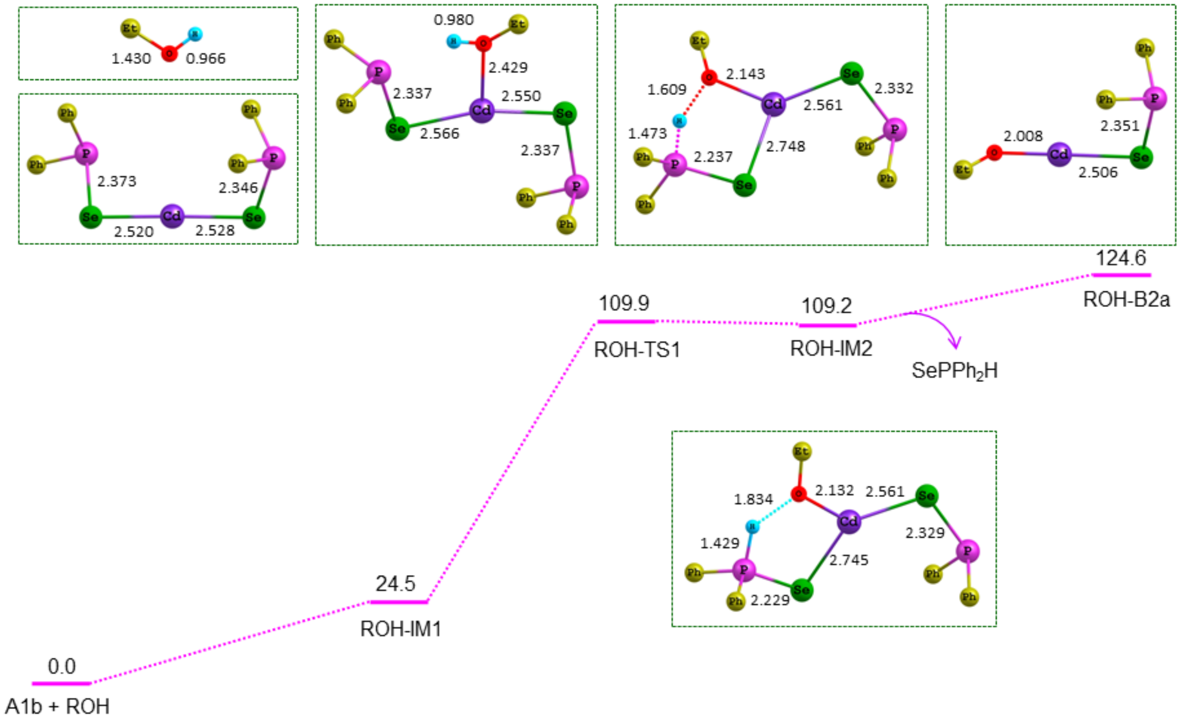

Supplementary Figure 30. The geometric structures of the species indicated and the schematic energy diagrams for the reaction of Ph_2PSe–Cd–$SePPh_2$ (**A1b**) + ROH → RO-CdSe-PPh_2 (**ROH-B2a**) + $SePPh_2H$ calculated at the M06//B3LYP/6-31++G(d, p), SDD level are shown. Bond lengths are reported in Å. Relative Gibbs free energies (G_r) (kJ mol^{-1}) for the corresponding species are shown relative to **A1b** + ROH. For the additive ROH, the activation energy barriers for the O–H bond cleavage is 109.9 kJ mol^{-1}, resulting in the release of $SePPh_2H$ from **A1b** to **B2a**.

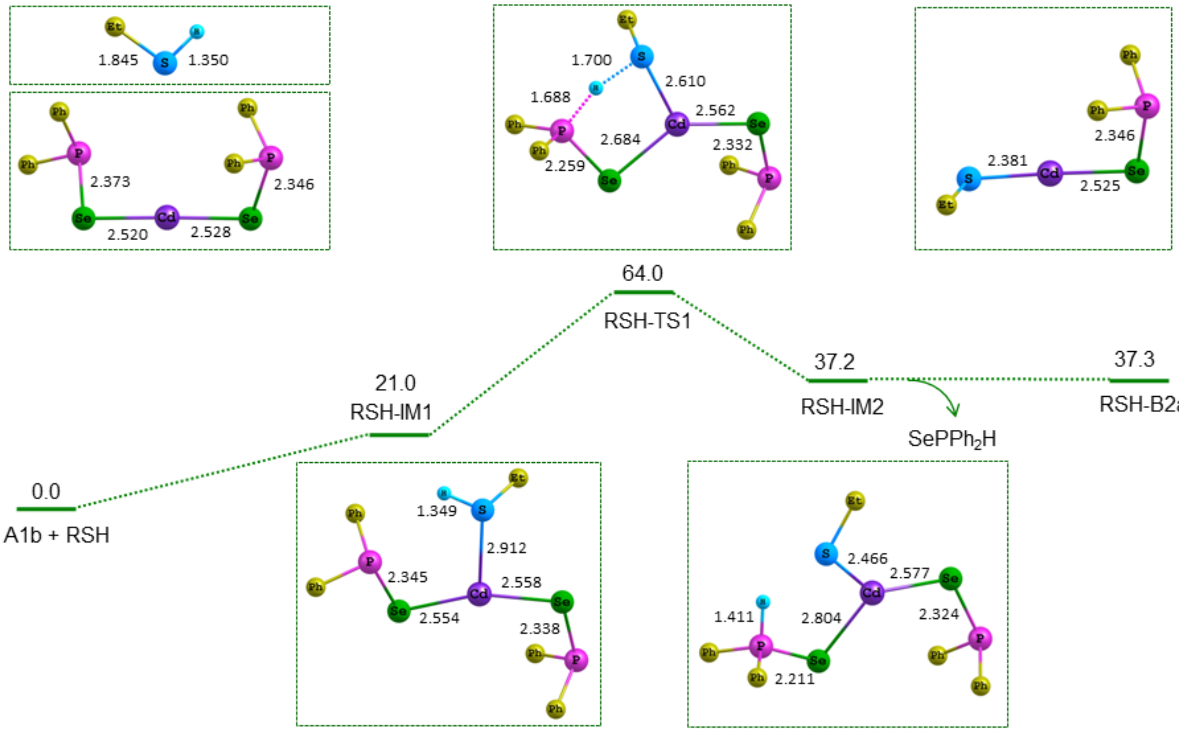

Supplementary Figure 31. The geometric structures of the species indicated and the schematic energy diagrams for the reaction of Ph_2PSe–Cd–$SePPh_2$ (**A1b**) + RSH → RS–CdSe–PPh_2 (**RSH-B2a**) + $SePPh_2H$ calculated at the M06//B3LYP/6-31++G(d, p), SDD level are shown. Bond lengths are reported in Å. Relative Gibbs free energies (G_r) (kJ mol^{-1}) for the corresponding species are shown relative to **A1b** + RSH. For the additive RSH, the activation energy barriers for the S–H bond cleavage is 64.0 kJ mol^{-1}, resulting in the release of $SePPh_2H$ from **A1b** to **B2a**.

Supplementary Table 1. ^{31}P NMR detected phosphorous-containing compounds **1** and **2** from reactions shown in Figs 1-5 and Supplementary Figs 1-5. Their chemical shifts are listed. Due to the equilibrium of **1** + $E=PPh_2H$ ⇔ **2** + $HPPh_2$ (Supplementary Table 2 and Note 2), when the assignment of **1** is correct, **2** ($Ph_2P(E)$–Y) should be straightforward. Thus, we further computed the ^{31}P NMR chemical shift of **1** (Ph_2P–Y, Y = –OOCR, $–PPh_2$, –NHR, –SH, and –OR) in toluene and in ODE, with H_3PO_4 as an external standard in water solution. Our calculated data (presented in parentheses with toluene above ODE) are obviously in good agreement with our experimental values (except for **1b**). (See Supplementary Note 1)

	$OOCC_{17}H_{33}$	PPh_2	$NHC_{17}H_{33}$	$SC_{12}H_{25}$	$OC_{12}H_{25}$	
Y	**a**	**b**	**c**	**d**	**e**	
	99 ppm	–14 ppm	42 ppm	29 ppm	112 ppm	experiment
**1**: Ph_2P–Y	(90 ppm)	(–3 ppm)	(43 ppm)	(28 ppm)	(118 ppm)	DFT-toluene
	(93 ppm)	(5 ppm)	(45 ppm)	(28 ppm)	(119 ppm)	DFT-ODE
**2**: $Ph_2P(Se)$–Y	77 ppm $J_{P\text{-}Se}$ (Hz) = 870	37/–13 ppm	58 ppm $J_{P\text{-}Se}$ (Hz) = 775	56 ppm $J_{P\text{-}Se}$ (Hz) = 800	85 ppm $J_{P\text{-}Se}$ (Hz) = 820	experiment
**2'**:$Ph_2P(S)$–Y	NA	45/–14 ppm	NA	NA	NA	experiment

Supplementary Table 2. Relative Gibbs free energies (ΔG_r) (kJ mol^{-1}) to **1** (Ph_2P–Y) + E=PPh_2H for all the species in the reaction of **1** (PH_2P–Y) + E=PPh_2H ⇔ **2** ($PH_2P(E)$–Y) + $HPPh_2$ calculated at the M06//B3LYP/6-31++G(d, p), SDD level in 1-octadecene under room temperature (298.15 K) and atmospheric pressure (1 atm). Y = –NHR, –SR, –OR, –OOCR, and –PPh_2 (with R = C_2H_5); E = S, Se, and Te. The DFT examination is in agreement with experimental observation. The equilibriums about the Se change are weighted towards the right, except for **1b** + Se=PPh_2H ⇔ **2b** + $HPPh_2$. Corresponding chemical reactions performed could be found elsewhere, with Ref 30/25 for c (Se), Supplementary Fig. 1 for d (Se), Supplementary Fig. 2 for e (Se), Ref 2/24 for a (Se), and Ref 31/21 for b (S and Se). (See Supplementary Note 2)

Reaction equations	ΔG_r		
	E		
	S	Se	Te
c Ph_2P–NHR + E=PPh_2H → $Ph_2P(E)$–NHR + $HPPh_2$	−25.8	−17.8	−11.9
d Ph_2P–SR + E=PPh_2H → $Ph_2P(E)$–SR + $HPPh_2$	−3.3	8.6	10.0
e Ph_2P–OR + E=PPh_2H → $Ph_2P(E)$–OR + $HPPh_2$	−36.8	−24.5	−18.6
a Ph_2P–OOCR + E=PPh_2H → $Ph_2P(E)$–OOCR + $HPPh_2$	−12.5	8.3	19.0
b Ph_2P–PPh_2 + E=PPh_2H → $Ph_2P(E)$–PPh_2 + $HPPh_2$	−3.9	34.9	32.9

Supplementary Table 3. Optimized geometric structures of typical **A** ($M(EPPh_2)_2$) species. Relative Gibbs free energies (G_r) (kJ mol^{-1}) to **A1a** (Ph_2PE–M–$EPPh_2$, E = Se, M = Cd) for all the species calculated at the M06//B3LYP/6-31++G (d, p), SDD level in ODE media. G_r in ODE solution and in gas phase are presented without and with parentheses, respectively. Also, some bond lengths (in Å) calculated are indicated in black; the NBO charges of P, Se, and Cd calculated are indicated in red. Such calculation level, G_r and the expression with bond length in black and charges in red apply to the rest of the tables (Supplementary Tables 4–19, 24 and 25). Our results suggest that the trend of energy differences between conformations in gas phase is, mainly, similar to that in ODE.

Species	Geometric Structure	G_r	Species	Geometric Structure	G_r
**A1a**	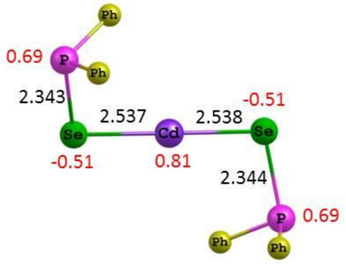	0.0 (0.0)	**A1b**	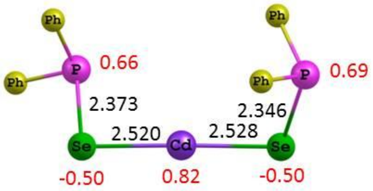	4.6 (3.9)
**A2**	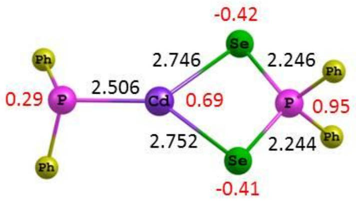	18.7 (1.4)	**A3**	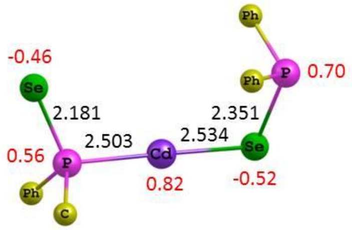	41.2 (44.8)
**A4**	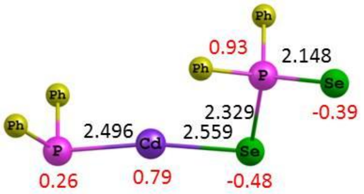	51.6 (56.2)	**A5**	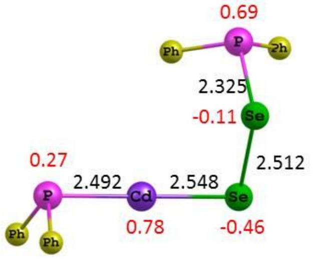	88.3 (77.1)
**A6**	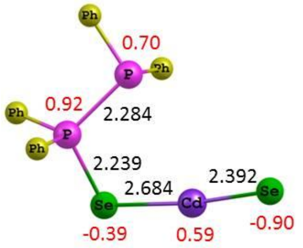	95.1 (125.3)	**A7**	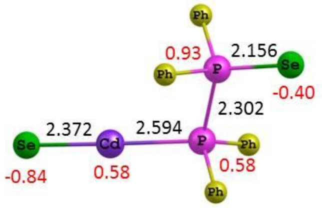	113.7 (141.0)

Supplementary Table 4. Optimized geometric structures of typical **B** ($MEPPh_2Y$) species. Relative Gibbs free energies (G_r) (kJ mol^{-1}) to **B1a** (E–M–PPh_2Y, E = Se, M = Cd, Y = –NHR, –SR) for all the species calculated.

Species	Geometric Structure	G_r	Species	Geometric Structure	G_r
		Y = –NHR			
**B1a**	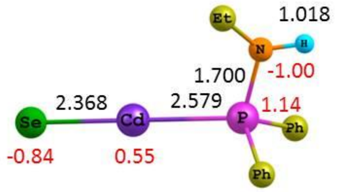	0.0 (0.0)	**B1b**	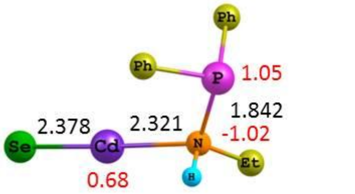	15.2 (33.2)
**B2a**	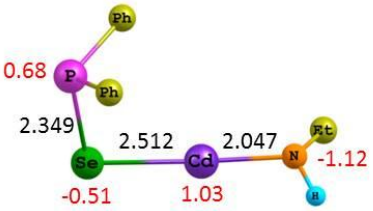	10.1 (–11.5)	**B2b**	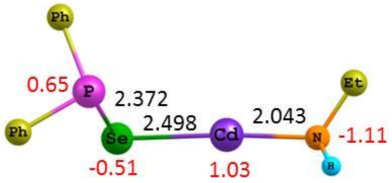	14.6 (–14.9)
**B3**	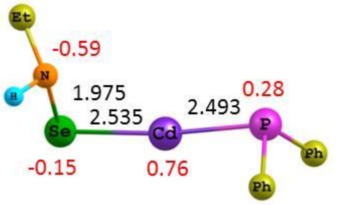	56.5 (25.9)			
		Y = –SR			
**B1a**	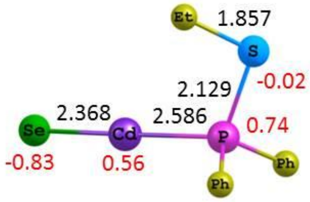	0.0 (0.0)	**B1b**	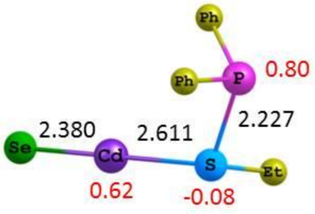	17.0 (19.0)
**B2a**	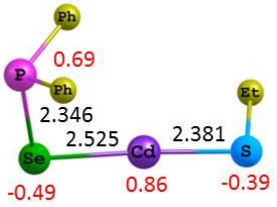	–82.5 (–94.8)	**B2b**	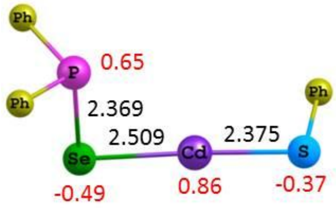	–78.0 (–100.3)
**B3**	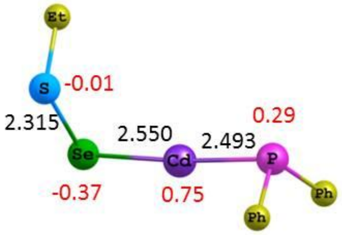	–3.3 (30.6)			

Supplementary Table 5. Optimized geometric structures of typical **B** ($MEPPh_2Y$) species. Relative Gibbs free energies (G_r) (kJ mol^{-1}) to **B1a** (E–M–PPh_2Y, E = Se, M = Cd, Y = –OR) for all the species calculated.

Species	Geometric Structure	G_r	Species	Geometric Structure	G_r
**B1a**	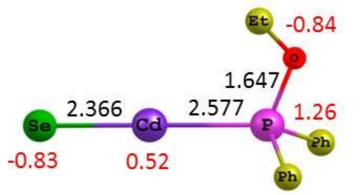	0.0<br>(0.0)	**B1b**	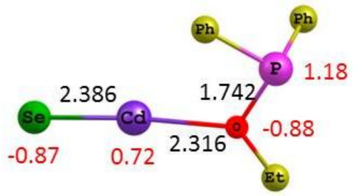	25.7<br>(35.9)
**B2a**	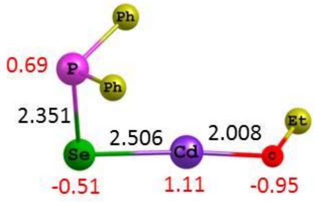	–6.9<br>(–19.3)	**B2b**	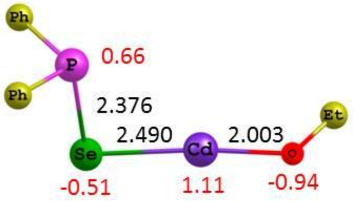	–1.4<br>(–22.0)
**B3**	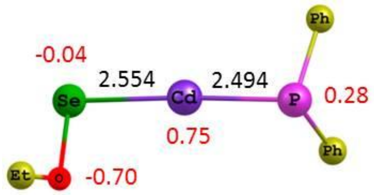	92.3<br>(63.3)			

Supplementary Table 6. Optimized geometric structures of typical **B** ($MEPPh_2Y$) species. Relative Gibbs free energies (G_r) (kJ mol^{-1}) to **B1a** (E–M–PPh_2Y, E = Se, M = Cd, Y= –OOCR, –PPh_2) for all the species calculated.

Species	Geometric Structure	G_r	Species	Geometric Structure	G_r
		Y = –OOCR			
**B1a**	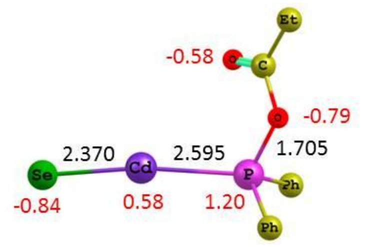	0.0 (0.0)	**B1b**	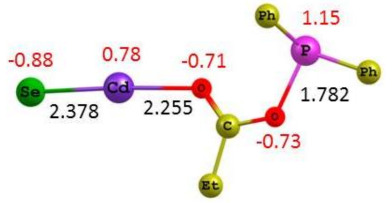	28.2 (16.5)
**B2a**	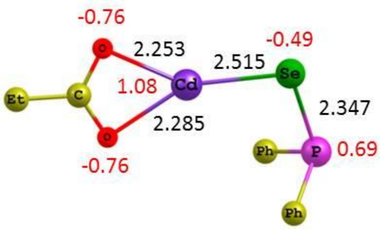	–111.3 (–118.7)	**B2b**	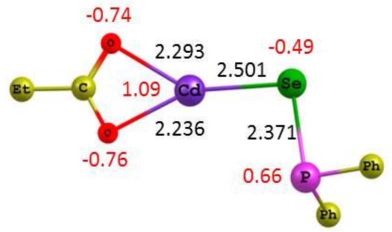	–104.9 (–119.3)
**B3**	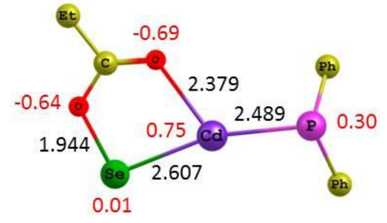	29.5 (–10.7)	**B4**	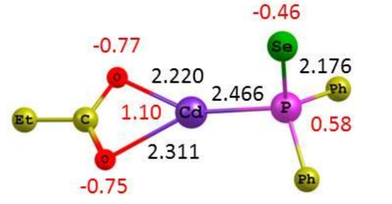	–69.8 (–75.6)
		Y= –PPh_2			
**B1**	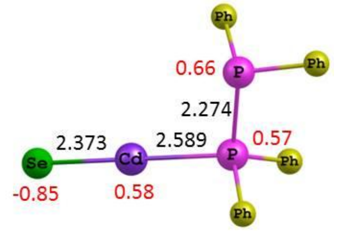	0.0 (0.0)			
**B2a**	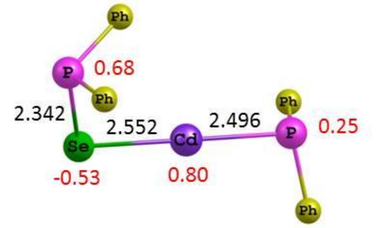	–58.7 (–89.3)	**B2b**	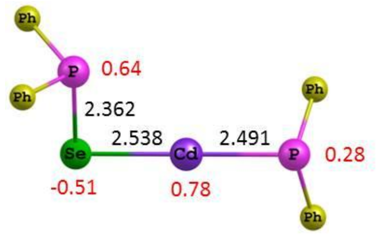	–49.7 (–91.5)

Supplementary Table 7. Optimized geometric structures of typical **C** (ME_2PPh_2Y) species. Relative Gibbs free energies (G_r) (kJ mol^{-1}) to **C1** (E–M–$EPPh_2Y$, E = Se, M = Cd, Y = –NHR) for all the species calculated.

<table>
<tr><th>Species</th><th>Geometric Structure</th><th>G_r</th><th>Species</th><th>Geometric Structure</th><th>G_r</th></tr>
<tr><td>C1</td><td>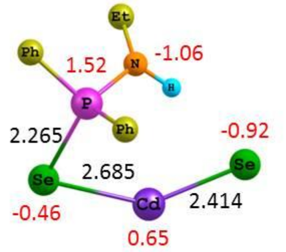
</td><td>0.0
(0.0)</td><td>C2</td><td>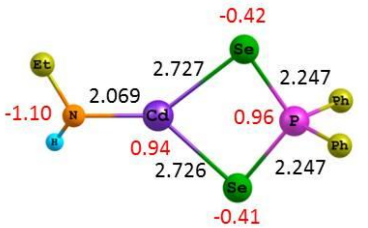
</td><td>18.7
(–22.5)</td></tr>
<tr><td>C3a</td><td>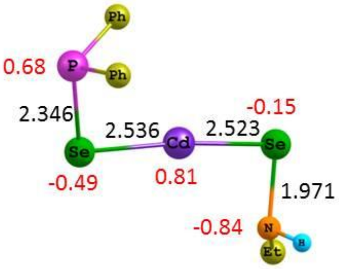
</td><td>43.4
(13.3)</td><td>C3b</td><td>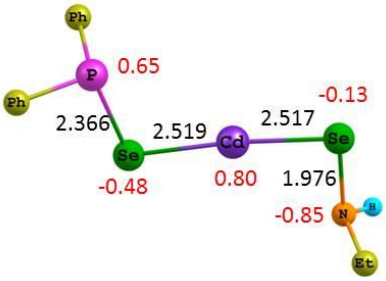
</td><td>46.6
(8.3)</td></tr>
<tr><td>C4</td><td>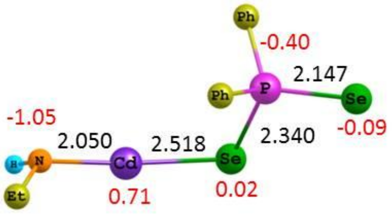
</td><td>48.2
(23.7)</td><td>C5</td><td>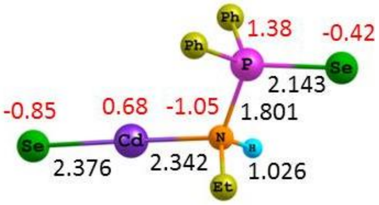
</td><td>50.9
(58.1)</td></tr>
<tr><td>C6</td><td>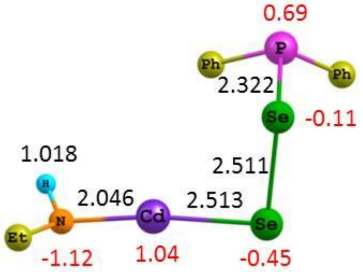
</td><td>81.8
(46.9)</td><td>C7</td><td>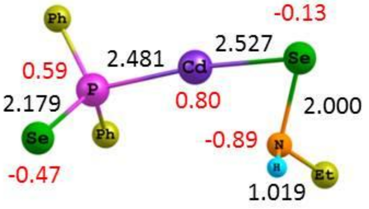
</td><td>90.4
(54.6)</td></tr>
</table>

Supplementary Table 8. Optimized geometric structures of typical **C** (ME_2PPh_2Y) species. Relative Gibbs free energies (G_r) (kJ mol^{-1}) to **C1** (E–M–$EPPh_2Y$, E = Se, M = Cd, Y = –SR) for all the species calculated.

Species	Geometric Structure	G_r	Species	Geometric Structure	G_r
**C1**	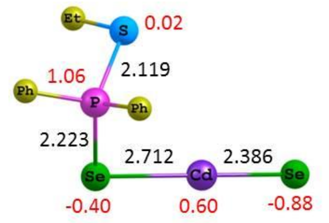	0.0 (0.0)	**C2**	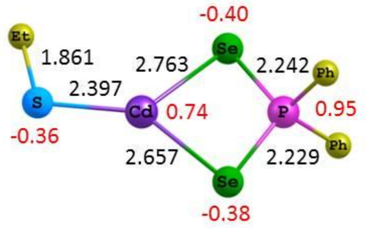	–125.1 (–155.4)
**C3a**	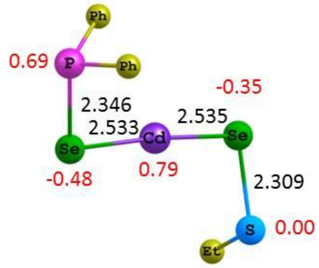	–54.3 (–82.1)	**C3b**	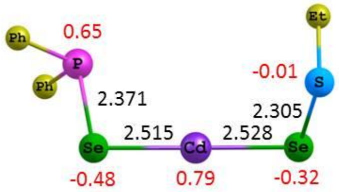	–46.1 (–85.1)
**C4**	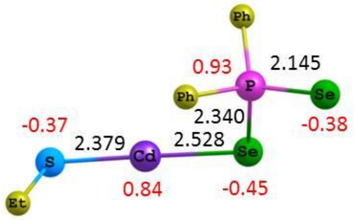	–77.3 (–92.3)	**C5**	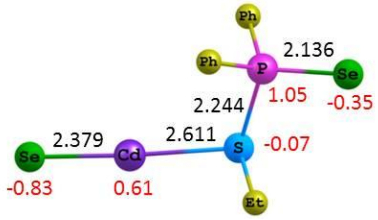	32.6 (25.2)
**C6**	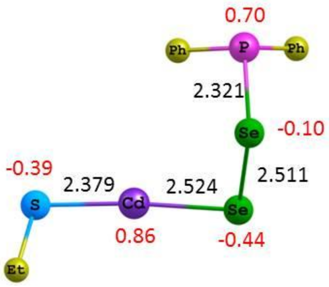	–46.9 (–71.2)	**C7**	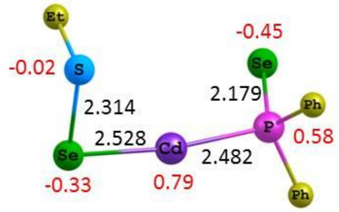	–10.9 (–38.7)

Supplementary Table 9. Optimized geometric structures of typical **C** (ME_2PPh_2Y) species. Relative Gibbs free energies (G_r) (kJ mol^{-1}) to **C1** (E–M–$EPPh_2Y$, E = Se, M = Cd, Y = –OR) for all the species calculated.

Species	Geometric Structure	G_r	Species	Geometric Structure	G_r
**C1**	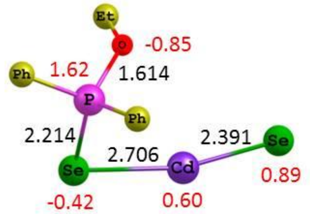	0.0<br>(0.0)	**C2**	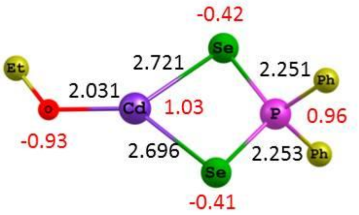	–2.3<br>(–34.5)
**C3a**	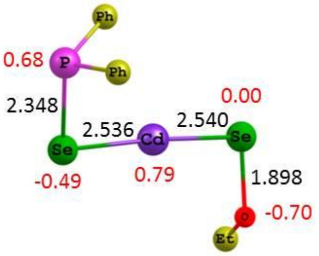	74.0<br>(44.8)	**C3b**	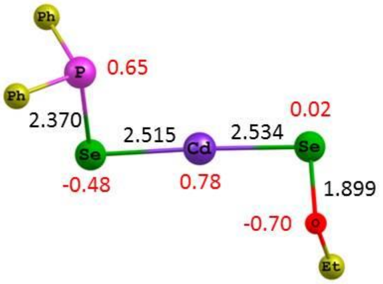	89.1<br>(50.0)
**C4**	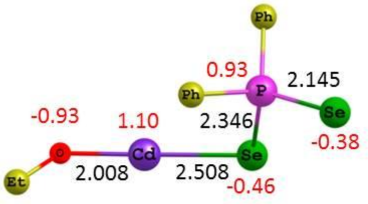	34.3<br>(17.7)	**C5**	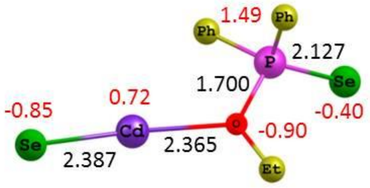	54.2<br>(56.3)
**C6**	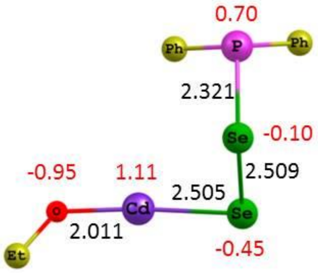	65.1<br>(38.1)	**C7**	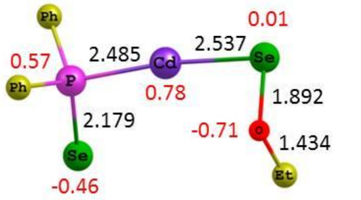	118.0<br>(85.6)

Supplementary Table 10. Optimized geometric structures of typical **C** (ME_2PPh_2Y) species. Relative Gibbs free energies (G_r) (kJ mol[-1]) to **C1** (E–M–$EPPh_2Y$, E = Se, M = Cd, Y= –OOCR) for all the species calculated.

Species	Geometric Structure	G_r	Species	Geometric Structure	G_r
**C1**	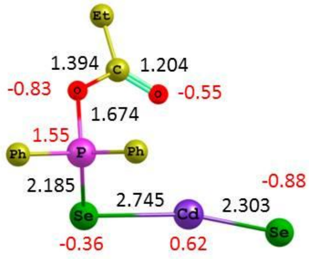	0.0<br>(0.0)	**C2**	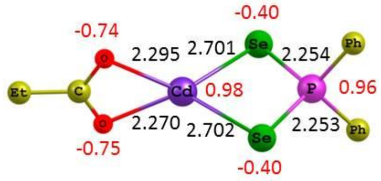	–128.5<br>(–154.7)
**C3a**	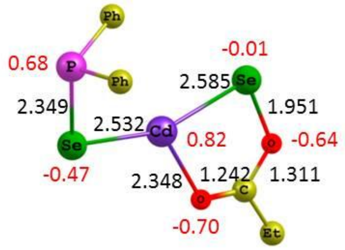	–8.5<br>(–42.2)	**C3b**	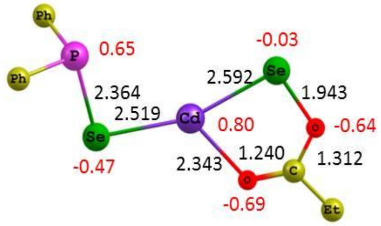	0.6<br>(–48.2)
**C3c**	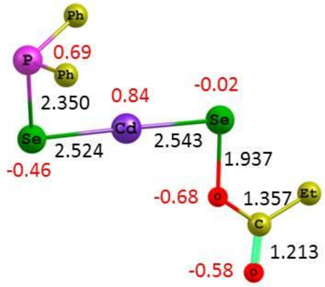	43.6<br>(20.5)	**C4**	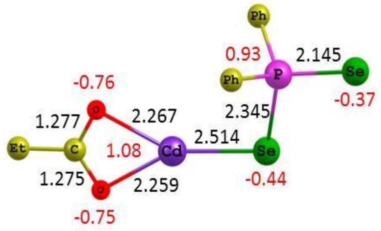	–89.4<br>(–100.3)
**C5**	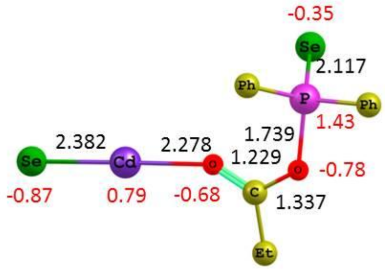	41.2<br>(29.2)	**C6**	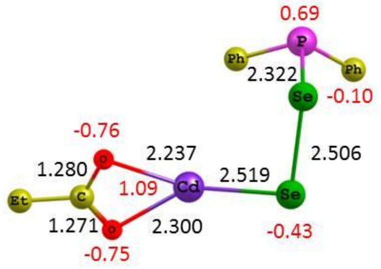	–61.8<br>(–78.1)
**C7**	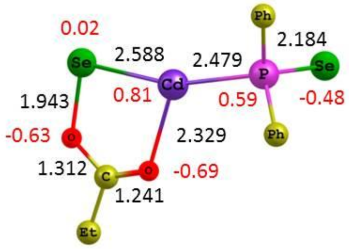	38.0<br>(–4.8)			

Supplementary Table 11. Optimized geometric structures of typical **E** ($M_2E_2(PPh_2)_2Y_2$) species. Relative Gibbs free energies (G_r) (kJ mol^{-1}) to **E1a** ($YPh_2P–M(E)_2M–PPh_2Y$, E = Se, M = Cd, Y = –NHR, –SR) for all the species calculated.

Species	Geometric Structure	G_r	Species	Geometric Structure	G_r
Y = –NHR					
**E1a**	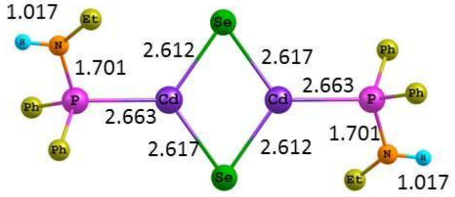	0.0 (0.0)	**E2**	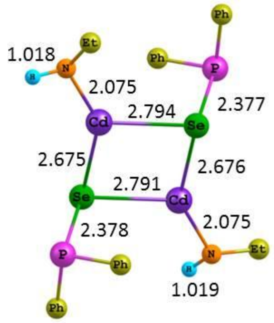	153.0 (102.9)
**E1b**	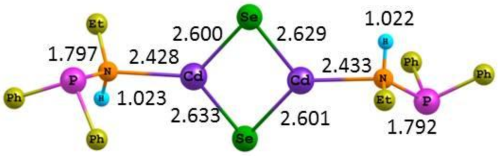	0.6 (–43.8)			
Y = –SR					
**E1a**	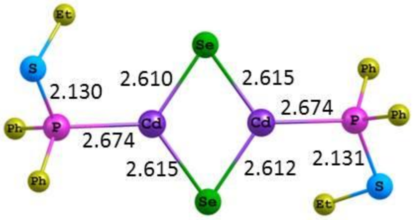	0.0 (0.0)	**E2**	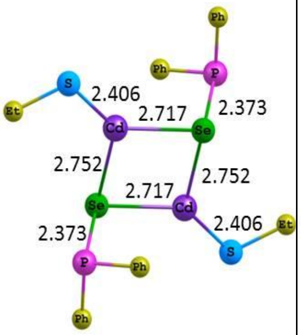	–32.7 (–19.7)
**E1b**	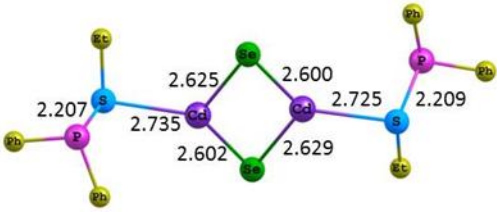	34.8 (41.9)			

Supplementary Table 12. Optimized geometric structures of typical **E** ($M_2E_2(PPh_2)_2Y_2$) species. Relative Gibbs free energies (G_r) (kJ mol^{-1}) to **E1a** ($YPh_2P–M(E)_2M–PPh_2Y$, E = Se, M = Cd, Y = –OR, –PPh_2) for all the species calculated.

Species	Geometric Structure	G_r	Species	Geometric Structure	G_r
Y = –NHR					
**E1a**	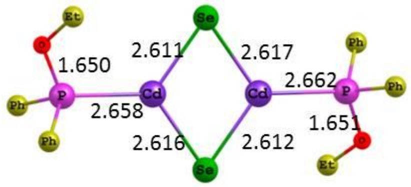	0.0 (0.0)	**E2**	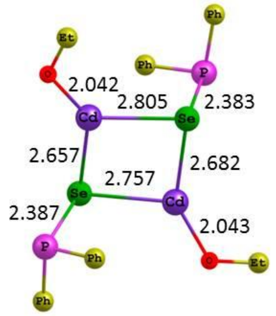	127.8 (136.2)
**E1b**	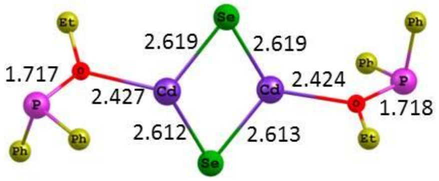	38.4 (64.5)			
Y = –PPh_2					
**E1**	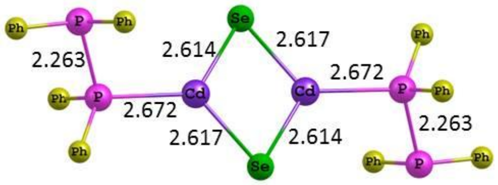	0.0 (0.0)	**E2**	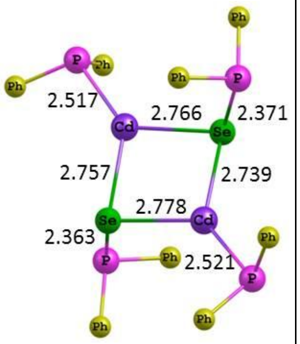	17.5 (10.6)

Supplementary Table 13. Optimized geometric structures of typical **E** ($M_2E_2(PPh_2)_2Y_2$) species. Relative Gibbs free energies (G_r) (kJ mol^{-1}) to **E1a** ($YPh_2P–M(E)_2M–PPh_2Y$, E = Se, M = Cd, Y = –OOCR) for all the species calculated. Relative Gibbs free energies in ODE solution and in gas phase are outside and in parentheses, respectively.

Y	Species	Geometric Structure	G_r
-OOCR	**E1a**	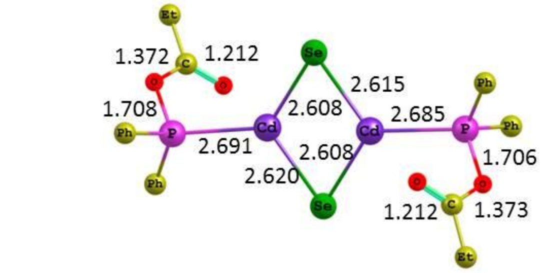	0.0<br>(0.0)
	**E2**	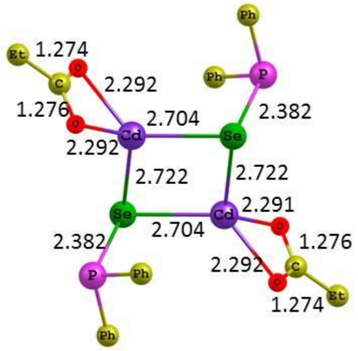	–107.3<br>(–90.8)
	**E1b**	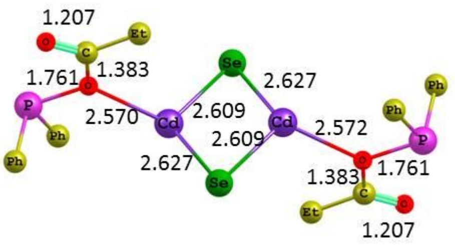	27.5<br>(179.5)
	**E1c**	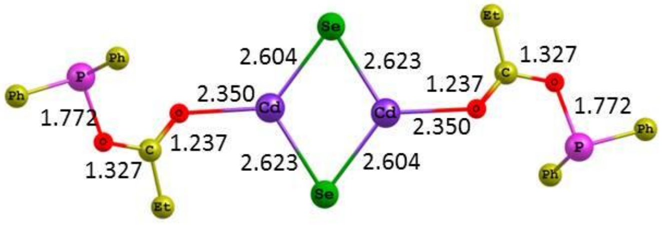	59.9<br>(16.4)

Supplementary Table 14. Optimized geometric structures of typical **F** ($M_2E_2(EPPh_2)_2Y_2$) species. Relative Gibbs free energies (G_r) (kJ mol^{-1}) to **F1** ($YPh_2P–E–M(E)_2M–E–PPh_2Y$, E = Se, M = Cd, Y = –NHR) for all the species calculated. Relative Gibbs free energies in ODE solution and in gas phase are outside and in parentheses, respectively.

<table>
<tr><th>Species</th><th>Geometric Structure</th><th>G_r</th><th>Species</th><th>Geometric Structure</th><th>G_r</th></tr>
<tr><td>F1</td><td>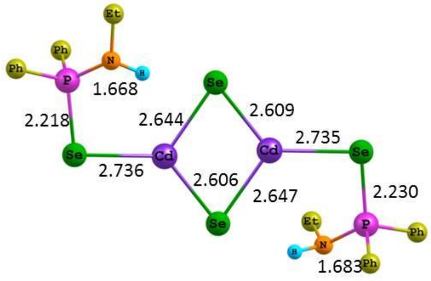
</td><td>0.0
(0.0)</td><td>F2</td><td>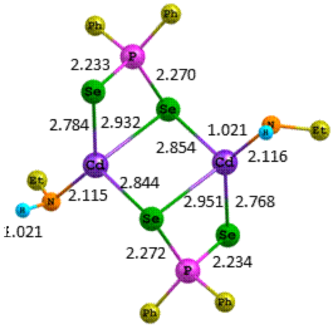
</td><td>196.6
(163.2)</td></tr>
<tr><td>F3a</td><td>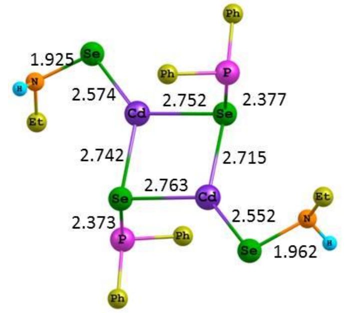
</td><td>205.8
(199.4)</td><td>F3b</td><td>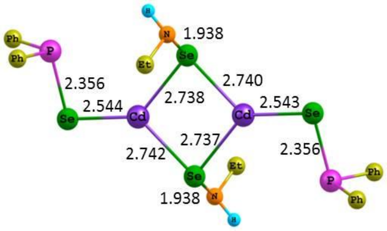
</td><td>231.4
(203.8)</td></tr>
<tr><td>F4</td><td>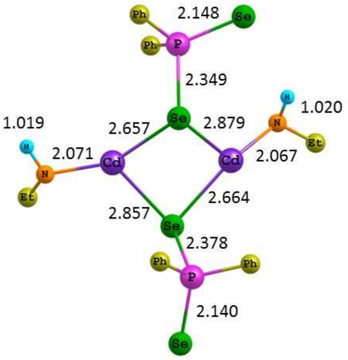
</td><td>258.0
(244.5)</td><td>F6</td><td>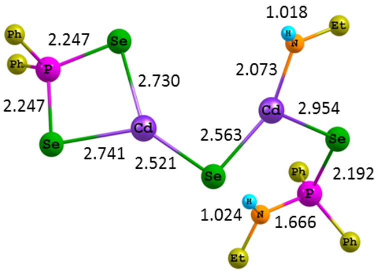
</td><td>96.6
(52.5)</td></tr>
<tr><td>F5</td><td>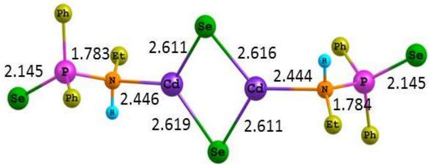
</td><td>56.2
(88.2)</td><td></td><td></td><td></td></tr>
</table>

Supplementary Table 15. Optimized geometric structures of typical **F** ($M_2E_2(EPPh_2)_2Y_2$) species. Relative Gibbs free energies (G_r) (kJ mol^{-1}) to **F1** ($YPh_2P–E–M(E)_2M–E–PPh_2Y$, E = Se, M = Cd, Y = –SR, –OR) for all the species calculated. Relative Gibbs free energies in ODE solution and in gas phase are outside and in parentheses, respectively.

Species	Geometric Structure	G_r	Species	Geometric Structure	G_r
Y = –SR					
**F1**	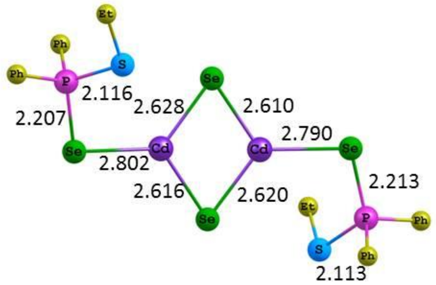	0.0<br>(0.0)	**F2**	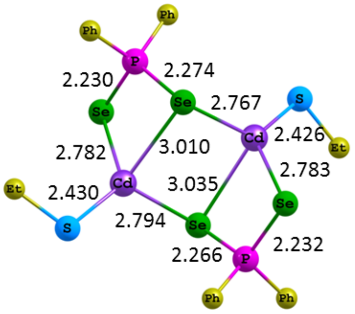	–78.0<br>(–87.4)
**F3**	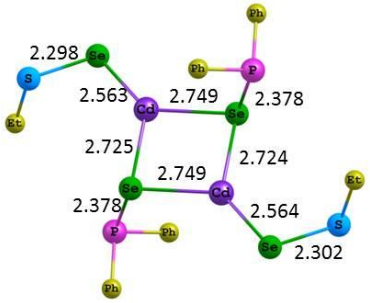	10.6<br>(9.5)			
Y = –OR					
**F1**	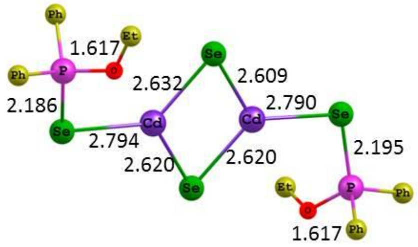	0.0<br>(0.0)	**F2**	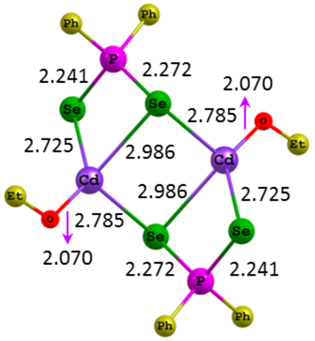	126.1<br>(96.3)
**F3**	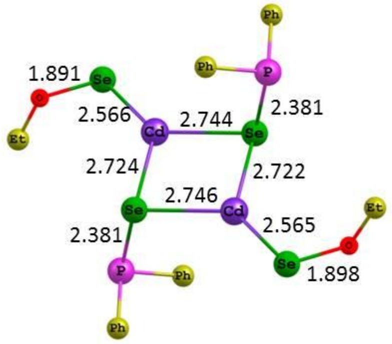	245.8<br>(228.3)			

Supplementary Table 16. Optimized geometric structures of typical **F** ($M_2E_2(EPPh_2)_2Y_2$) species. Relative Gibbs free energies (G_r) (kJ mol^{-1}) to **F1** ($YPh_2P–E–M(E)_2M–E–PPh_2Y$, E = Se, M = Cd, Y = –OOCR) for all the species calculated. Relative Gibbs free energies in ODE solution and in gas phase are outside and in parentheses, respectively.

Y	Species	Geometric Structure	G_r
-OOCR	**F1**	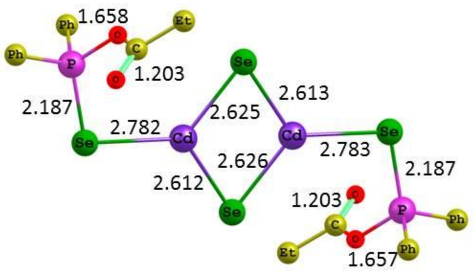	0.0<br>(0.0)
	**F2**	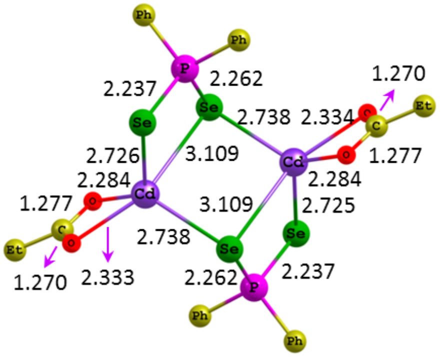	–78.8<br>(–94.7)
	**F3**	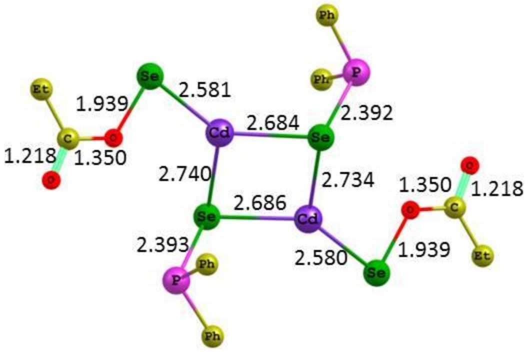	227.5<br>(218.4)

Supplementary Table 17. Optimized geometric structures of typical **D** ($M_2E_2(EPPh_2)_2(PPh_2)_2$) species. Relative Gibbs free energies (G_r) (kJ mol^{-1}) to **D1** (YPh_2P–E–$M(E)_2M$–E–PPh_2Y, E = Se, M = Cd, Y = –PPh_2) for all the species calculated. Relative Gibbs free energies in ODE solution and in gas phase are outside and in parentheses, respectively.

Y	Species	Geometric Structure	G_r
-PPh_2	**D1**	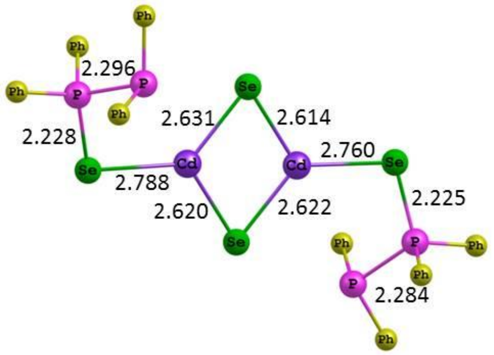	0.0<br>(0.0)
	**D2**	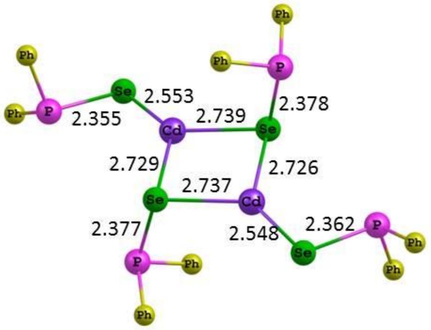	–86.6<br>(–106.5)
	**D3**	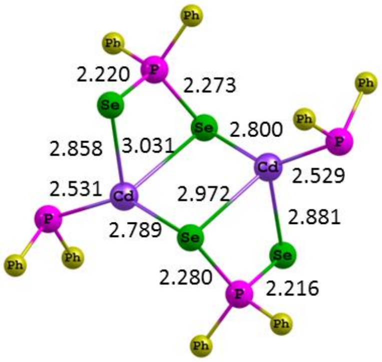	–51.1<br>(–52.5)

Supplementary Table 18. Relative Gibbs free energies (G_r) (kJ mol^{-1}) to **B1** (E–M–PPh$_2$Y, E = S, and Te, M = Cd, Y = –NHR) for all the species calculated. HOMO and LUMO are indicated (contour value: 0.030), which are similar for S and for Te.

E	Species	Geometric Structure	G_r	HOMO	LUMO
S	**B1**	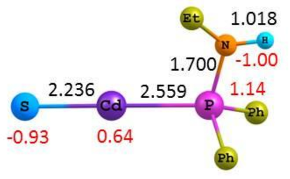	0.0	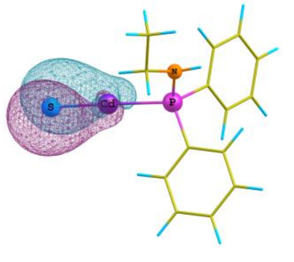	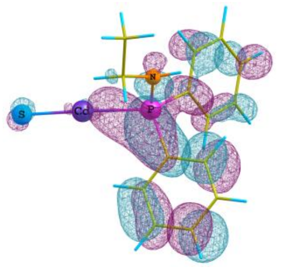
	**B2**	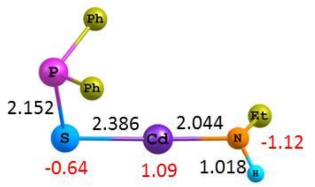	–24.3	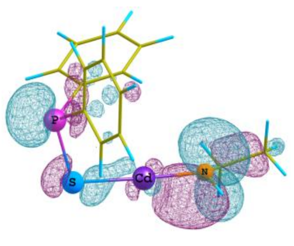	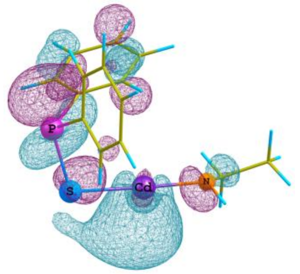
Te	**B1**	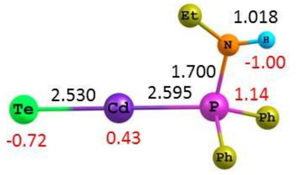	0.0	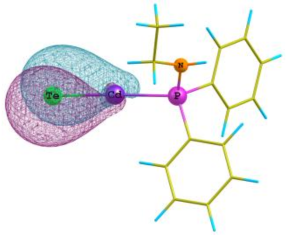	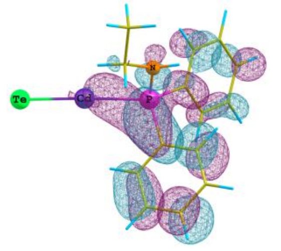
	**B2**	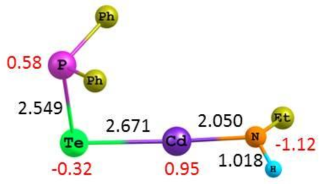	26.6	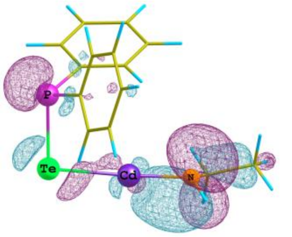	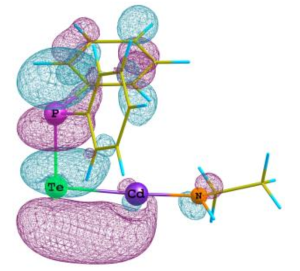

Supplementary Table 19. Relative Gibbs free energies (G_r) (kJ mol^{-1}) to **C1** (E–M–EPPh$_2$Y, E = S, and Te, M = Cd, Y = –NHR) for all the species calculated. HOMO and LUMO are indicated (contour value: 0.030). The former is similar for S and for Te.

E	Species	Geometric Structure	G_r	HOMO	LUMO
S	**C1**	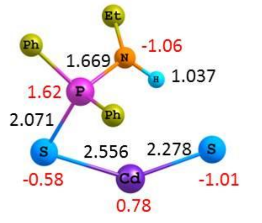	0.0	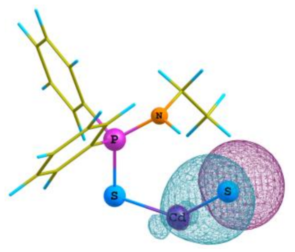	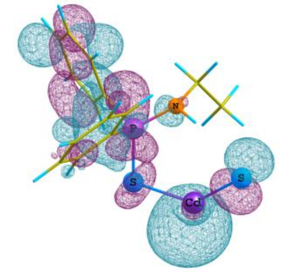
	**C2**	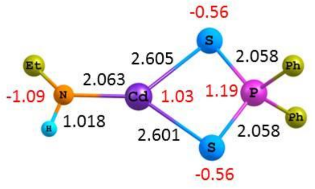	–30.9	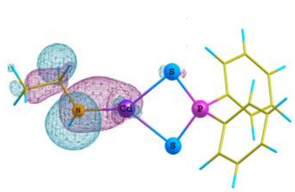	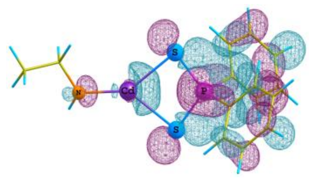
Te	**C1**	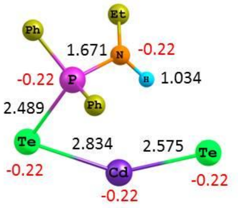	0.0	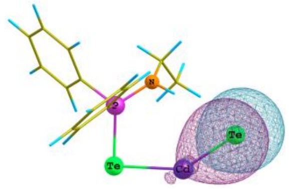	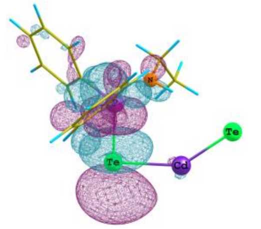
	**C2**	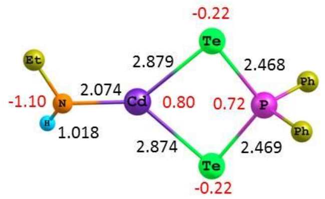	46.7	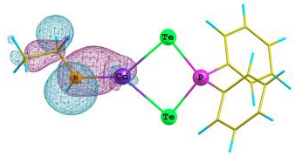	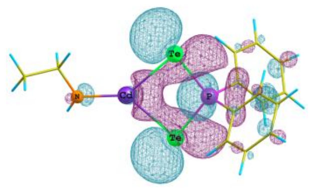

Supplementary Table 20. Relative Gibbs free energies (G_r, kJ mol^{-1}) to **A1a** (Ph_2PE–M–$EPPh_2$, E = Se, M = Cd) for all the species. The energies of HOMO (E_{HOMO}, eV) and LUMO (E_{HOMO}, eV) are indicated.

		–PPh_2			
Species	Isomer	G_r	E_{HOMO}	E_{LUMO}	ΔE
**A1a**	*trans*-Ph_2PE-M-$EPPh_2$	0.0	–6.0334	3.4998	9.5332
**A1b**	Ph_2PE-M-$EPPh_2$	4.6	–6.0632	3.4245	9.4877
**A2**	Ph_2P-M-E_2-PPh_2	18.7	–5.3995	3.5759	8.9754
**A3**	$Ph_2P(E)$-M-$EPPh_2$	41.2	–5.4757	3.3132	8.7889
**A4**	Ph_2P-M-$EP(E)Ph_2$	51.6	–5.6353	3.3929	9.0282
**A5**	Ph_2P-M-E-$EPPh_2$	88.3	–5.6881	2.8581	8.5462
**A6**	E-M-$EP(Ph_2)$-PPh_2	95.1	–4.1962	3.0190	7.2152
**A7**	E-M- $PPh_2P(E)Ph_2$	113.7	–4.6110	3.0372	7.6482

Supplementary Table 21. Relative Gibbs free energies (G_r, kJ mol^{-1}) to **B1a** (E–M–PPh_2Y, E = Se, M = Cd, Y = –NHR, –SR, –OR, –OOCR, and –PPh_2) for all the species. The energies of HOMO (E_{HOMO}, eV) and LUMO (E_{HOMO}, eV) are indicated.

		–NHR				–SR			
Species	Isomer	G_r	E_{HOMO}	E_{LUMO}	ΔE	G_r	E_{HOMO}	E_{LUMO}	ΔE
**B1a**	E-M-PPh_2Y	0.0	–4.4578	3.3851	7.8429	0.0	–4.5876	3.1749	7.7625
**B1b**	E-M-YPPh_2	15.2	–4.5098	3.4317	7.9415	17.0	–4.6151	2.9261	7.5412
**B2a**	Ph_2PE-M-Y	10.1	–5.8686	3.6141	9.4827	–82.5	–6.2217	3.5073	9.7290
**B2b**	*trans*-Ph_2PE-M-Y	14.6	–5.9127	3.7934	9.7061	–78.0	–6.1966	3.5848	9.7814
**B3**	YE-M-PPh_2	56.6	–5.6347	3.6737	9.3084	–3.3	–5.7217	3.3753	9.0970

		–OR				–OOCR			
Species	Isomer	G_r	E_{HOMO}	E_{LUMO}	ΔE	G_r	E_{HOMO}	E_{LUMO}	ΔE
**B1a**	E-M-PPh_2Y	0.0	–4.5453	3.1914	7.7367	0.0	–4.4616	3.0378	7.4994
**B1b**	E-M-YPPh_2	25.7	–4.5834	3.1976	7.7810	28.2	–4.6307	–2.1769	2.4538
**B2a**	Ph_2PE-M-Y	–6.9	–6.2838	3.4613	9.7451	-111.3	–6.2820	3.4883	9.7703
**B2b**	*trans*-Ph_2PE-M-Y	–1.4	–6.2885	3.4928	9.7813	-104.9	–6.1235	3.4754	9.5989
**B3**	YE-M-PPh_2	92.3	–5.7761	3.5018	9.2779	29.5	–5.5932	3.1007	8.6939

		–PPh_2			
Species	Isomer	G_r	E_{HOMO}	E_{LUMO}	ΔE
**B1**	E-M-PPh_2Y	0.0	–4.3967	3.1562	7.5529
**B2a**	Ph_2PE-M-Y	–58.7	–5.1679	3.5306	9.1485
**B2b**	*trans*-Ph_2PE-M-Y	–49.7	–5.6777	3.6140	9.2917

Supplementary Table 22. Relative Gibbs free energies (G_r, kJ mol^{-1}) to **C1** (E–M–EPPh$_2$Y, E = Se, M = Cd, Y = –NHR, –SR, –OR, and –OOCR) for all the species. The energies of HOMO (E_{HOMO}, eV) and LUMO (E_{HOMO}, eV) are indicated.

		–NHR				–SR			
Species	Isomer	G_r	E_{HOMO}	E_{LUMO}	ΔE	G_r	E_{HOMO}	E_{LUMO}	ΔE
**C1**	E-M-EPPh$_2$Y	0.0	–4.6249	3.3517	7.9766	0.0	–4.1942	2.9000	7.0942
**C2**	Ph$_2$PE$_2$-M-Y	18.7	–5.4104	3.5646	8.9751	–125.1	–5.8343	3.4819	9.3162
**C3a**	Ph$_2$PE-M-EY	43.4	–6.0422	3.5124	9.5546	–54.3	–6.1333	3.2943	9.4276
**C3b**	*trans*-Ph$_2$PE-M-EY	46.6	–6.0323	3.4999	9.5322	–46.1	–6.1675	3.1972	9.3647
**C4**	Y-M-EP(E)Ph$_2$	48.2	–5.7416	-2.0694	3.6722	–77.6	–5.8319	3.2795	9.1114
**C5**	E-M-YP(E)Ph$_2$	50.9	–4.7536	3.2066	7.9602	32.6	–4.8003	2.6655	7.4658
**C6**	Y-M-E-EPPh$_2$	81.8	–5.8927	2.7812	8.6739	–46.9	–6.2652	2.7197	8.9849
**C7**	Ph$_2$P(E)-M-EY	90.4	–5.4194	-2.1350	3.2844	–10.9	–5.5380	3.1480	8.6860
		–OR				–OOCR			
Species	Isomer	G_r	E_{HOMO}	E_{LUMO}	ΔE	G_r	E_{HOMO}	E_{LUMO}	ΔE
**C1**	E-M-EPPh$_2$Y	0.0	–4.4105	3.1728	7.5833	0.0	–4.1071	3.0307	7.1378
**C2**	Ph$_2$PE$_2$-M-Y	–2.3	–6.1950	3.3876	9.5826	–128.5	–6.4041	3.4326	9.8367
**C3a**	Ph$_2$PE-M-EY	74.0	–6.1683	3.3013	9.4696	–8.5	–5.9909	3.1948	9.1857
**C3b**	*trans*-Ph$_2$PE-M-EY	89.1	–6.1902	3.3399	9.5300	0.6	–5.7951	2.9954	8.9705
**C4**	Y-M-EP(E)Ph$_2$	34.3	–5.8893	3.2541	9.1433	–89.4	–5.8340	3.2404	9.0804
**C5**	E-M-YP(E)Ph$_2$	54.2	–4.8153	3.0137	7.8289	41.2	–4.6523	-2.2433	2.4090
**C6**	Y-M-E-EPPh$_2$	65.1	–6.3175	2.6485	8.9659	–61.8	–6.2813	2.7271	9.0084
**C7**	Ph$_2$P(E)-M-EY	118.0	–5.5519	3.2155	8.7674	38.0	–5.3625	2.8629	8.2254

Supplementary Table 23. Relative Gibbs free energies (G_r) (kJ mol^{-1}) to 2**A** + 2HY for all the species calculated. (**A** = Ph_2PSe–Cd–$SePPh_2$, Y = –NHR, –SR, –OR, –OOCR, and –PPh_2). The energy value of the left column **B** includes 2**B** + 2Se=PPh_2H, **E** includes **E** + 2Se=PPh_2H, Ph_2P-Y includes 2Ph_2P-Y + Cd_2Se_2 + 2Se=PPh_2H, **C** includes 2**C** + 2$HPPh_2$, **F** includes **F** + 2$HPPh_2$ and $Ph_2P(Se)$–Y includes 2$Ph_2P(Se)$–Y + Cd_2Se_2 + 2$HPPh_2$.

	Y				
	NHR	SR	OR	OOCR	PPh_2
**D**					–48.4 (**D2**)
$Ph_2P(Se)$-PPh_2					117.5
**B**	272.0 （**B1a**）	84.0 （**B2a**）	272.1 （**B1a**）	117.6 （**B2a**）	190.3 （**B2a**）
**E**	111.9 （**E1a**）	27.6 （**E2**）	88.6 （**E1a**）	47.1 （**E2**）	114.1 （**E1**）
Ph_2P-Y	71.0	26.6	64.3	97.8	47.7
**C**	109.5 （**C1**）	–92.4 （**C2**）	110.2 （**C1**）	–35.7 （**C2**）	
**F**	–60.2 （**F1**）	–105.0 （**F2**）	–45.8 （**F1**）	–44.9 （**F2**）	
$Ph_2P(Se)$-Y	35.4	43.7	15.3	114.3	

Supplementary Table 24. Optimized geometric structures of typical stable **B** species (B: E–M–P(Y)Ph_2, E=Se, M=Cd, Y= –NHR, –SR, –OR, –OOCR, and –PPh_2). HOMO and LUMO are indicated (contour value: 0.030).

Species	Geometric Structure	HOMO	LUMO
**B1a**<br>Y =<br>–NHR	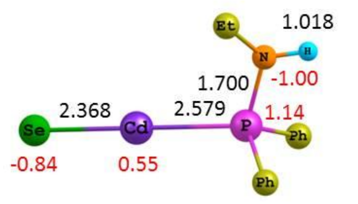	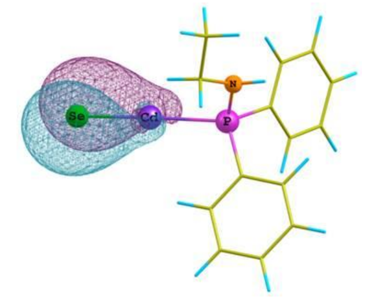	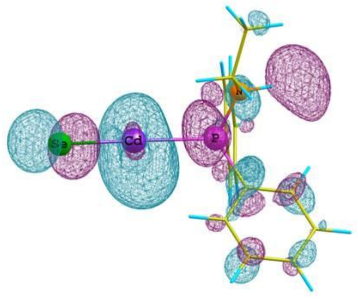
**B2a**<br>Y = –SR	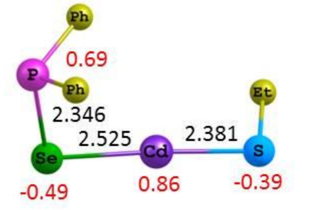	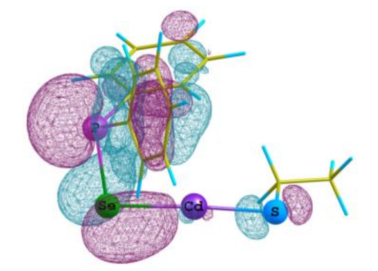	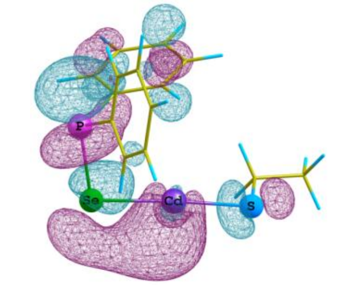
**B2a**<br>Y = –OR	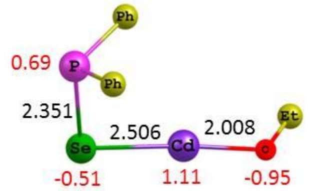	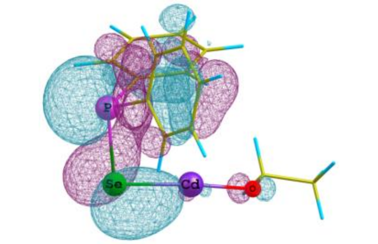	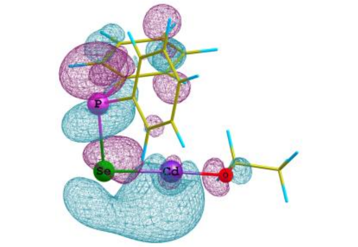
**B2a**<br>Y =<br>–OOCR	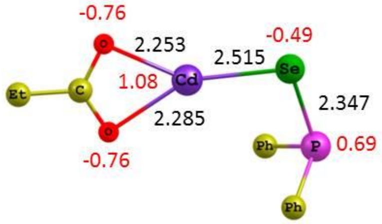	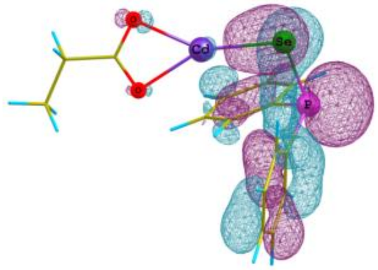	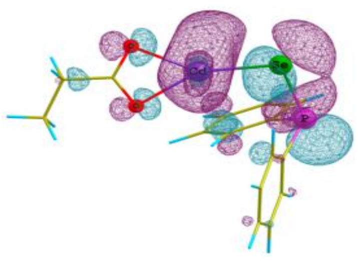
**B1**<br>Y =<br>–PPh_2	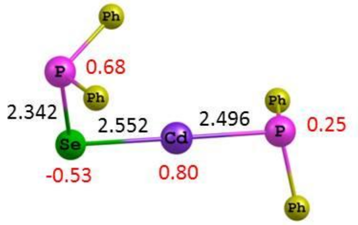	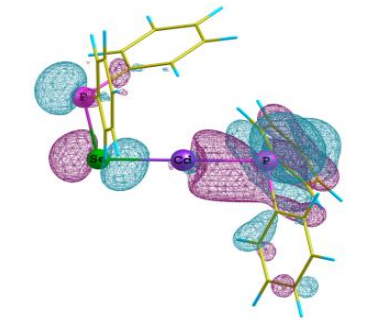	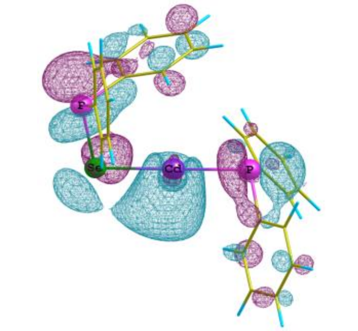

Supplementary Table 25. Optimized geometric structures of typical stable **A** (Ph_2PE–M–$EPPh_2$) and **C** (E–M–$EPPh_2Y$), E = Se, M = Cd, Y = –NHR, –SR, –OR, and –OOCR). HOMO and LUMO are indicated (contour value: 0.030).

Species	Geometric Structure	HOMO	LUMO
**A1a**	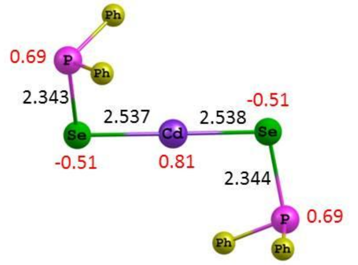	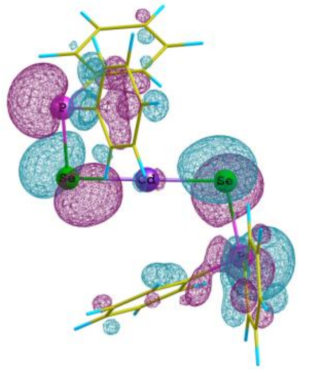	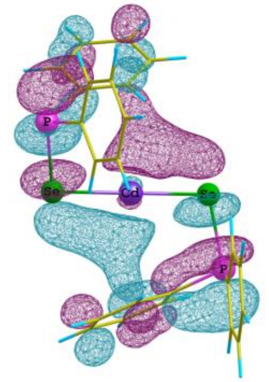
**C1**<br>Y =<br>–NHR	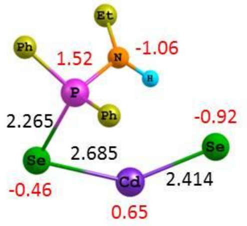	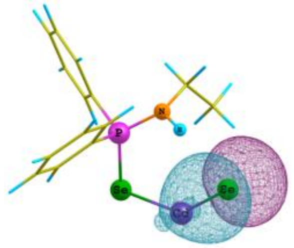	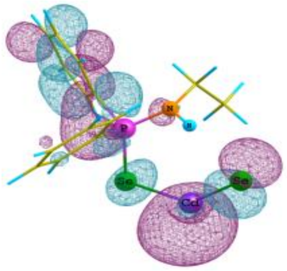
**C2**<br>Y = –SR	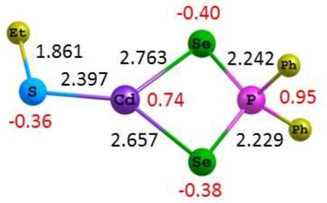	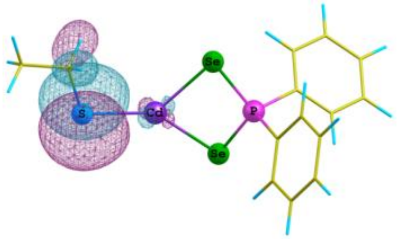	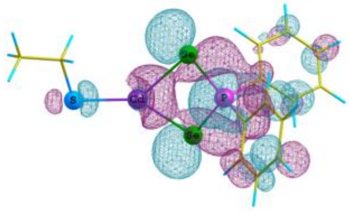
**C2**<br>Y = –OR	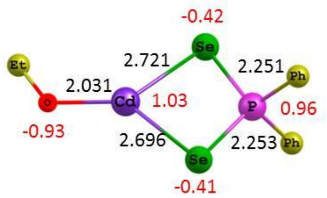	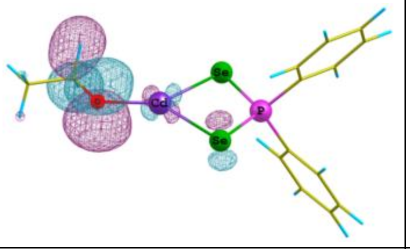	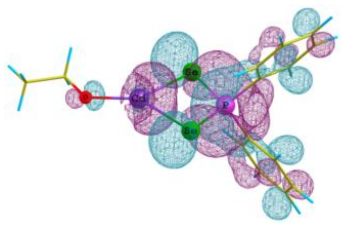
**C2**<br>Y =<br>–OOCR	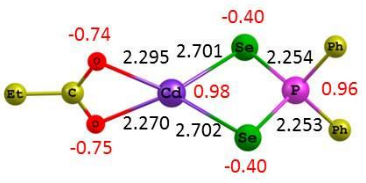	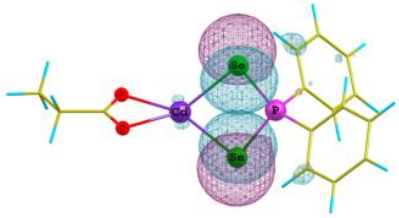	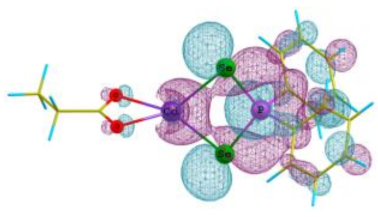

Supplementary Note 1. ^{31}P NMR study of Compound Ph_2P–Y (**1**) and $Ph_2P(E)$–Y (**2**).

As indicated in Supplementary Table 1, for the NMR spectrum calculation, a gauge-including-atomic-orbital (GIAO) method at the M06 /6-31++G(d, p), SDD theoretical level was used[16-19]. A polarized continuum model (PCM-SMD)[20,21] was utilized to simulate the solvent effects of toluene and 1-octadecene (ODE) with dielectric constant = 2.0. Full geometry optimizations were carried out to locate all of the stationary points both in toluene and ODE solutions, via a hybrid M06 functional method[22] and the 6-31++G(d, p) basis set[23,24], namely M06 /6-31++G(d, p). Systematic harmonic frequency calculations were performed to ensure all the structures obtained are true minima on the potential energy surfaces.

The DFT calculation for Me_3P=E provides the insight into the nature of the P=E bond and the thermal and photochemical instability of compounds with terminal P–Te bonds[25].

Previously, Compound **1a** and **1b** and **2a** were detected from the syntheses of PbSe[27], CdSe[2,27-30], ZnS[31], ZnSe[31], ZnSeS[31], and CdSeS NCs[32]. Ref 17 and Ref 18 reported Compound **1a** with Ph_2P–OOCPh (102 ppm), in addition to **1b**. Compound **1c** and **2c** were detected from the syntheses of CdSe in the presence of a primary amine $C_{12}H_{25}NH_2$ (with **2c** of $Ph_2P(Se)$–$NHC_{12}H_{25}$, 57 ppm)[27] or $C_{17}H_{33}NH_2$[30]. Compound **1d** was reported from the synthesis of Cu_2S, In_2S_3, and $CuInS_2$ in the presence of a primary thiol $C_{12}H_{25}SH$[15]. For details, see below (Ref x/y, x is what numbered below and y is what numbered in the main text)

Ref 31/21 (ACS Appl. Mater. Interfaces 2012) reported the synthesis of ZnSe, and ZnSeS alloyed QDs at 80 °C - 300 °C (Fig. 2 and Supplementary Fig. 2) and the ^{31}P NMR spectra collected at **80 °C** for ZnSe, ZnS and ZnSeS (Fig. 8). **1a** (99 ppm), **2a** (77 ppm) and **1b** (–14 ppm) were obtained.

Ref 15/22 (ACS Appl. Mater. Interfaces 2013) reported the synthesis of $CuInS_2$ alloyed QDs at from 50 °C - 160 °C (Figs 1 and 3, and Supplementary Fig. 1) and the in situ ^{31}P NMR spectra (Fig. 2) of the three reactions of CuI + S=PPh_2H, $InOAc_3$ + S=PPh_2H, and CuI + $InOAc_3$ + S=PPh_2H at **100 °C**. **1d** (29 ppm) was obtained.

Ref 2/23 (Angew. Chem. 2013) reported the in situ ^{31}P NMR spectra collected from 25 °C up to **140 °C** (Supplementary Fig. 4) of the three reactions of (a) $CdOA_2$ + SeTOP + $HPPh_2$, (b) $CdOA_2$ + SeTOP, and (c) $CdOA_2$ + Se=PPh_2H. **1a** (99 ppm) and **1b** (–14 ppm) were obtained for the first and last reactions. Absorption and emission spectra were also shown for the three reactions.

Ref 32/38 (Chem. Mater. 2016) reported the synthesis of CdSe, CdS and CdSeS alloyed QDs at 100 °C - 240 °C (Fig. 4) and the ^{31}P NMR spectra collected at **120 °C** (Fig. 5 and Supplementary Fig. 5a) which is the temperature to obtain CdSe and CdS and CdSeS QDs. **1a** (99 ppm) and **1b** (–14 ppm) were obtained.

Ref 26/15 (JACS 2010) performed **the study close to room temperature** (with **1a**, **2a**, and **1b** detected), to explore the effect of the unavoidable impurity $HP(C_8H_{17})_2$ in commercial $P(C_8H_{17})_3$ on the formation of the Pb–Se bond. Per this JACS 2010 (Ref 26) said that the growth temperature of current syntheses are high (~300 °C for CdSe) but with poor conversion yield (< 2 %) and reproducibility. Also, the typically synthetic

method has remained largely unchanged, with the use of tertiary phosphine chalcogenides and metal salts. Such a statement still holds, with a few papers addressing the use of a commercial secondary phosphine $HPPh_2$ to carry out syntheses at lower temperature but with enhanced yield and reproducibility (such as Ref 32/38).

To study the reaction mechanism of one organic reaction, such as glycosylation, it has been widely accepted to perform NMR at lower temperature (as compared to usual reaction temperatures) with even additives to trap intermediates. For example,

Nature Chem. ***4***, 663–667 (2012) "Dissecting the mechanisms of a class of chemical glycosylation using primary ^{13}C kinetic isotope effects". The NMR experiments were carried out at –72 °C.

Nature Chem. ***7***, 186–191 (2016) "Catching elusive glycosyl cations in a condensed phase with HF/SbF_5 superacid" The NMR experiments were carried out at –50 – 0 °C.

For a defined chemical reaction, the mechanism of the reaction is not dependent on its reaction temperature in a certain range. There is a required minimum energy to overcome the activation energy for the transition state of the rate-determining step; the reaction could only proceed above that corresponding temperature. In general, reaction mechanistic studies are carried out in a temperature range in which reaction intermediates could be "observed" (sometimes at very low temperature but within a suitable instrument operation window), along the progress of the reaction monitored by the decrease of starting materials or increase of products. The possible side reactions because of thermally instability of products should also be considered; and lower reaction temperature could avoid such side reactions.

Our mechanistic study is based on the monitoring of phosphorus compounds, which are formed accompanied by the monomer formation. The NMR monitoring was carried out at RT and up to 120 °C, depending on the individual experiment and the purpose of one experiment. It has to be pointed out that addition of $HPPh_2$ (to traditional approach such as $CdOA_2$ + SeTOP) led to monomer formation at much lower temperature. On the other hand, using relatively high temperatures with optimized temperature control in real QDs syntheses (such as for ZnSeS[31], CdSeS[32], and $CuInS_2$[15]) is to control nucleation and growth for the quality of photoluminescence of the resulting QD product.

Thus, it is reasonable to conclude or assume that our study has followed general mechanistic study methodology to study the monomer formation under realistic reaction conditions of the approach addressed.

The assignment of **1a**, **2a**, **1b**, **2b**, **1c**, and **2c** were addressed in the previous study (for **2** with E= Se). Refs 2 and 30 reported the direct syntheses of Compound **1a** and **2a**, and Compound **1c** and **2c,** respectively. Ref 31 dealt with the assignment of Compound **1a**, **1b**, **2b** and **2b'** (with E = S). Here, the experimental data that corroborates the assignment of Compound **1d** and **2d** is addressed in Supplementary Fig. 1, while Compound **1e** and **2e** in Supplementary Fig. 2. For details, see below

**1a**: oleoyloxy diphenylphosphine	Ref 31/21 S. Fig. 9A.	**1b** + RCOOH ↔ **1a** + $HPPh_2$
	Ref 2/24 S. Fig. 1a.	RCOONa + $ClPPh_2$ → **1a** + NaCl
	Ref 26/15 Table 1	
**2a**: oleoyloxy selenodiphenylphosphate	Ref 2/24 S. Fig. 1a.	**1a** + Se=PPh_2H ↔ **2a** + $HPPh_2$
	Ref 26/15 Table 1	

**1b**: tetraphenyldiphosphine	Ref 31/21 Fig. 9B. Commercial 1b purchased.	
	Ref 2/24 S. Fig. 1a.	**1a** + $HPPh_2$ → **1b** + RCOOH
	Ref 26/15 Table 1.	
	Ref 27/16 S. Fig. 3.	
**2b**: tetraphenyldiphosphine monoselenide	Ref 31/21 Fig. 9C.	**1b** + $Se{=}PPh_2H$ ↔ **2b** + $HPPh_2$
**2b′**: tetraphenyldiphosphine monosulfide	Ref 31/21 Fig. 9D.	**1b** + $S{=}PPh_2H$ ↔ **2b′** + $HPPh_2$
**1c**: oleylamino diphenylphosphine	Ref 30/25 S. Fig. 2A.	**2c** + $Se{=}PPh_2H$ ↔ **1c** + $HPPh_2$
	Ref 30/25 S. Fig. 2B.	RNH_2 + $ClPPh_2$ → **1c** + HCl
**2c**: oleylamino diphenylphosphine selenide	Ref 30/25 S. Fig. 2A.	$Ph_2P(Se)Cl$ + RNH_2 → **2c** + HCl
**1d**: dodecylthio diphenylphosphine	present study Supplementary Fig. 1.	
**2d**: dodecylthio diphenylphosphine selenide	present study Supplementary Fig. 1.	
**1e**: dodecyl diphenylphosphinite	present study Supplementary Fig. 2.	
**2e**: dodecyl selenodiphenylphosphate	present study Supplementary Fig. 2.	

It should be of help to provide the reason for the names provided for Compound **1** ($Ph_2P{-}Y$) and **2** ($Ph_2P(E){-}Y$) above. Basically, we tried to find similar compounds and their names provided (listed below); due to literature inconsistency (such as Chem Commun. 2005 vs Ref 26/15 and Ref 28/34), we also followed the principle indicated below.

In order to name our P-containing compound **1** and **2**, similar compounds were searched.

$CH_3COO{-}PPh_2$	acetoxydiphenylphosphinite	*J. C. S. Perkin I* 2416-2422 (1977)
$C_{17}H_{33}COO\text{-}PPh_2$	oleyloxydiphenylphosphine	*J. Am. Chem. Soc.* ***134***, 1400–1403 (2012)
$CH_3COO{-}P(Se)Ph_2$	acetoxy selenodiphenylphosphinite	*Chem. Commun.* 2692–2694 (2005)
$C_{17}H_{33}COO\text{-}P(Se)Ph_2$	9-octadecenoxydiphenylphosphine	*J. Am. Chem. Soc.* ***132***, 10973–10975 (2010)
$C_{17}H_{33}COO\text{-}P(Se)Ph_2$	oleyloxydiphenylphosphine selenide	*J. Am. Chem. Soc.* ***134***, 1400–1403 (2012)
$Ph_2P\text{-}PPh_2$	tetraphenyldiphosphine	*Can. J. Chem.* 49, 994–100 (1971)
$Ph_2P(S)PPh_2$	Tetraphenyldiphosphine monosulfide	*Chem. Commun.* **50**, 4707–4710 (2014)
$MeNH{-}PPh_2$	methylamino diphenylphosphine	*J. C. S. Dalton* 1424–1428 (1976)
$EtNH{-}PPh_2$	ethylamino diphenylphosphine	*Tervalent P-N Chem.* 1543–1547 (1964)
$EtNH{-}P(Se)Ph_2$	ethylamino diphenylphosphine selenide	
$EtS{-}PPh_2$	ethylthio diphenylphosphine	*J. Raman Spectrosc.* **23**, 107–124 (1992)
$EtS{-}P(Se)Ph_2$	ethylthio diphenylphosphine selenide	
$EtO{-}PPh_2$	ethyl diphenylphosphinite	*J. C. S. Perkin I* 2416 –2422 (1977)
$EtO{-}P(Se)Ph_2$	ethyl selenodiphenylphosphinite	
Ph_3P	triphenylphosphine	
Ph_3PSe	triphenylphosphine selenide	

We also followed the principle
phosphine + Se → phosphine selenide
phsophinite + Se → seleno phosphate

Supplementary Note 2. The reaction of **1** ($Ph_2P–Y$) + $E{=}PPh_2H$ ⇔ **2** ($Ph_2P(E)–Y$) + $HPPh_2$.

As shown in Supplementary Table 2, it is noteworthy that these E exchange reactions could be considered as "side reactions" in our reaction systems, but affecting the amount of **1**, **2**, and $HPPh_2$ observed. The amount of $HPPh_2$ could also be affected by other "side reactions" such as Reactions 6 and 7 listed below.

For example, the increase of **1a** and **1b** is accompanied by the disappearance of **$HPPh_2$** as demonstrated by Fig. 1 (about the reaction of $CdOA_2$ + SeTOP + $HPPh_2$ in our main text where the SeTOP amount is very low). For the reaction of $CdOA_2$ + SeTOP + $HPPh_2$, the formation of **1a**, **1b**, and **2a** have been documented by Refs 29/23 and 2/24 to be related to Cd–Se bond formation. And the related reactions or so-called background experiments have been investigated and reported which are summarized below (Ref x/y, where x is what numbered below and y is what numbered in the main text):

1) $CdOA_2$ + SeTOP — Refs 33/32 and 23/23 (S. Fig. 4A-b)
2) SeTOP + $HPPh_2$ ⇔ TOP + $Se{=}PPh_2H$ — Ref 26/15 suggested and Ref 33/23 demonstrated by ^{31}P NMR (S. Fig. 3)
3) $CdOA_2$ + TOP ⇔ TOP-$Cd(OA)_2$ complex — Ref 33/23 by ^{31}P NMR (Fig. 1)
4) $CdOA_2$ + $HPPh_2$ no reaction (140 °C) — Ref 33/23 by ^{31}P NMR (Fig. 1 and S. Fig. 2). **1b** (–14 ppm) was not detected, suggesting that R6/15 pathway is not related to Cd^0.
5) $CdOA_2$ + $Se{=}PPh_2H$ — Ref 33/23 by ^{31}P NMR (Fig. 2 and S. Figs 4A-c and 5A collected at -55 °C and up to 100 °C). **1a** and **1b** and **$HPPh_2$** were obtained.
Ref 2/24 by ^{31}P NMR (Fig. 1). **1a**, **2a**, **1b** and **$HPPh_2$** were obtained, with their relative amounts depending on Cd/Se feed ratios. Their formation was discussed in detail to be related to the monomer Cd_1Se_1 formation.

With a crossover reagent $Se{=}P(C_6H_{11})_2H$ instead of $Se{=}PPh_2H$,

5) $CdOA_2$ + $Se{=}P(C_6H_{11})_2H$ — Ref 2/24 by ^{31}P NMR (S. Table 1, C_6H_{11} = Cy) (S. Fig. 3b) **1a** (Cy_2P-OOCR, 134 ppm) and **2a** ($Cy_2P(Se)$-OOCR, 116 ppm) were obtained.
6) RCOOH + Ph_2P-PPh_2 (**1b**) → Ph_2P-COOR (**1a**) + $HPPh_2$ — Ref 31/21 by ^{31}P NMR (Fig. 9)
7) Ph_2P-COOR (**1a**) + $HPPh_2$ → RCOOH + Ph_2P-PPh_2 (**1b**) — Ref 2/24 by ^{31}P NMR (S. Fig. 1a)
8) Ph_2P-COOR (**1a**) + $Se{=}PPh_2H$ ⇔ Ph_2P-COOR (**2a**) + $HPPh_2$ — Ref 2/24 by ^{31}P NMR (S. Fig. 1c)
9) Due to the fast reaction of $CdOA_2$ + $Se{=}PPh_2H$ shown by Ref 29/23 (S. Fig. 5A taking place even at –55 °C),
Ref 2/24 designed a relatively slow reaction of $Cd(Se_2PPh_2)_2$ (**3**) + $CdOA_2$ + $HPPh_2$ to study the formation of monomer Cd_1Se_1. **1a**, **2a** and **1b** were obtained and their relative amount is affected by the feed molar ratios of the three starting materials.
Ref 2/24 further investigated the slow reaction with the presence of $Cd(OOCPh)_2$ instead of $CdOA_2$ or PhCOOH, and **1a** (Ph_2P–OOCPh, 102 ppm) and **2a** ($Ph_2P(Se)$–OOCPh, 79 ppm) were obtained, as shown by (Supplementary Table 1, Note 1, and Fig. 4).

So, according to the background experiments reported, the formation of the P-containing chemicals **1** ($Ph_2P–Y$) and **2** ($Ph_2P(E)–Y$) is related to the formation of monomers/solutes/NCs.

Simultaneously, there are “not-directly-related reactions” or so called “side-reactions” such as 2), 3), 6) – 8). At relatively high T, 6) is favored; at RT, 7) is favored. For the present RT reaction (such as Fig. 1a), the disappearance of $HPPh_2$ is in agreement with 7) reported and the increase of 1a demonstrates clearly that its formation is related to the monomer formation.

Supplementary Note 3. The comparison of the formation mechanisms of the monomers proposed by Ref 26 (JACS 2010) and by the present investigation, as shown in Supplementary Fig. 26. For the JACS 2010 study (top): the formation of **1a**, **2a**, and **1b** are from

2 eq. $RCOO(PbSe)PPh_2 \rightarrow$ **1a** ($Ph_2P–OOCR$) + $RCOO(PbSe)_2PPh_2$

2 eq. $RCOOPbSe_2PPh_2 \rightarrow$ **2a** ($Ph_2P(Se)–OOCR$) + $RCOOPbSePbSe_2PPh_2$

$Ph_2PPbPPh_2 \rightarrow$ **1b** ($Ph_2P–PPh_2$) + Pb^0

Note that the proposed path to **1b** does not address the experimental observation with the detection of **1a**, **2a**, and **1b** from $1PbOA_2$ + $1Se=PPh_2H$ and from $2CdOA_2$ + $1Se=PPh_2H$ at room temperature for 10 min (by its Figs S9 and S8, respectively). Although Figure S6 shows that **1b** was detected from $PbOA_2$ + $HPPh_2$ at 140 °C with Pb^0 observed after hours, its says no reaction was observed to occur for $CdOA_2$ + $HPPh_2$ at 140 °C for days.

For our study (bottom with the example of PbSe for comparison):

$Pb_2Se_2(PPh_2)_2Y_2$ (**E**) $\rightarrow$ **1** (**1a** $Ph_2P–OOCR$ or **1b** $Ph_2P–PPh_2$) + Pb_2Se_2

$Pb_2Se_2(SePPh_2)_2Y_2$ (**F**) $\rightarrow$ **2** (**2a** $Ph_2P(Se)–OOCR$) + Pb_2Se_2

Here, the formation of the P-containing compounds **1** and **2** is the latest step accompanied by the formation of M_2E_n monomers. Both our experimental and DFT investigations support our pathway proposed, which fits with different reaction systems and thus is GENERAL. Furthermore, we have used $Cd(Se_2PPh_2)_2$ + $Cd(OA)_2$ as one "captured" intermediate (Fig. 5 and Supplementary Fig. 24) to further support the reaction pathway proposed (Supplementary Fig. 25).

It is critical to point out that we compared the below two exoergic reactions; the energy barrier is about 4.2 kJ mol^{-1} for the two reactions. Obviously, what we proposed is much more favored than that by the work of JACS 2010, especially at low temperature.

$CdOA_2$ + 1SeDPP = $RCOOCdSePPh_2$ + RCOOH	(JACS 2010)	56.4 kJ mol^{-1}
$CdOA_2$ + 2SeDPP = $Ph_2PSeCdSePPh_2$ + 2RCOOH	(our study)	115.1 kJ mol^{-1}

Supplementary Note 4. The interaction between these intermediate molecules and solvent molecules.

With the recommended a_0 for SCRF calculation = 5.69 angstrom (10.75 Bohr) from the calculated mole volume (264 cm^3 mol^{-1}), and the dielectric constant of ε = 2.0 in the polarized continuum model (PCM-SMD), no simplification was applied in the present calculation for the solvent effect of 1-octadecene (ODE, $C_{16}H_{33}$–CH=CH_2), but with ethyl groups (C_2H_5) representing the alkyl group (R = -$C_{16}H_{33}$). Namely, ODE is represented by *n*-butene. As shown in Supplementary Fig. 27, for these complexes of **A1a**, **A1b**, **B1a**, **B1b**, **B2a**, **B2b**, and **B3** with *n*-butene, the relative Gibbs free energies are in the range of 22 kJ mol^{-1} to 37 kJ mol^{-1}. Thus, the interaction between these intermediate molecules and solvent molecules is very weak and the resulting complexes are unstable. Thereupon, the solvent molecules play a minor role on the stability of intermediate isomers/conformers.

The above consideration is more about electrostatics, while most people consider dispersion forces as the most important for VDW forces. There are four M06 schemes listed below[34] and we choose the first one for our intermediate molecules which contain "across-the-board" elements including metals.

(a) M06, good for transition metals and main group thermochemistry.
(b) M06-2X, not good for transition metals but excellent for main group chemistry.
(c) M06-L, the most accurate functional for transition metals.
(d) M06-HF .

For our intermediate molecules, M06 should have a reliable correction for dispersion forces. We look at delta E for our intermediate isomers. Based on our computation about electrostatics, we think, if there is some error with VDW forces considered, it should be small. If VDW effects are underestimated by M06, the error could be more or less cancelled. Thus, for our isomers, we think, VDW forces should not critically affect the energy landscape, namely the relative stability of the conformers suggested. VDW forces should also play a minor role on the relative stability of conformers proposed.

Supplementary Note 5. The kinetics associated with the putative pathway of the CdSe system: **A** + H–Y $\rightarrow$ **B** + Se=PPh_2H.

We have put efforts to address the kinetics associated with the putative pathway of the CdSe system: **A** + H–Y $\rightarrow$ **B** + Se=PPh_2H in Supplementary Fig. 25. We chose **A1b** (Supplementary Table 3) for **A** and **B2a** (Supplementary Tables 4-6) for **B**, with HY = RNH_2, $HPPh_2$, ROH and RSH; the geometric structures of the various species and the resulting schematic energy diagrams of the four reactions are shown as Supplementary Figs 28-31, respectively.

Therefore, regarding the typical reaction step of the H–Y (Y = N, P, O, and S) bond cleavage of the putative pathway of the CdSe system of **A** + H–Y $\rightarrow$ **B** + Se=PPh_2H, our computation suggests that the activation energy barriers decrease from H–N (148.1 kJ mol^{-1}), H–P (135.6 kJ mol^{-1}), H–O (109.9 kJ mol^{-1}), to H–S (64.0 kJ mol^{-1}). This kinetics trend supports our Fig. 1 experimental results showing that the disappearance of SeTOP is the slowest in Batch b) with HY = RNH_2. Also, this trend seems to be in agreement with our Fig. 5 experimental results showing that the disappearance of precursor **3** is the slowest in Batch a) with HY = RNH_2. Based on the energy calculation shown in Supplementary Tables 4-10 for **B** and **C**, it is reasonable that the two putative pathways of **A** + H–Y $\rightarrow$ **B** + Se=PPh_2H and **A** + H–Y $\rightarrow$ **C** + $HPPh_2$ share the same kinetics trend.

Supplementary Methods

Experimentation: for the present experimental study, its detailed information can be found elsewhere, such as the chemicals used, ^{31}P NMR, TEM, XRD and in situ absorption and emission measurements[2,15,29,31,35-39].

Chemicals. All chemicals used are commercially available from Sigma-Aldrich and were used as received (or otherwise specified). The metal sources are CdO (99.999 %), zinc oxide powder (ZnO, 99.24 % from J.T. Baker Chemical Co. (Philipsburg, NJ, USA)), PbO (99.999 %), germanium (II) chloride dioxane complex (1:1) ($GeCl_2$ • dioxane), copper (I) iodide (CuI, 98 %), and indium (III) acetate ($In(OAc)_3$, 99.99 %). The preparation of the corresponding metal precursors used followed published procedures for $Cd(OA)_2$[2,29,38], $Zn(OA)_2$[37,31], $Pb(OA)_2$[35,36], CuI/$SHC_{12}H_{25}$[15], and $In(OA)_3$[15]. 1-octadecene (ODE, tech. 90 %) was used. $Ge(OA)_2$ was prepared as following: the reaction of NaOA (4.538 mmol, 1.388 g, TCI, 97 %) with $GeCl_2$ (dioxane) (2.162 mmol, 0.5008 g, mw231.65) in ODE (9.0785 g), which was gently heated and stirred in glove box for about 1 hour to give cloudy solution. [$Ge(OA)_2$] = 2.162 mmol/10.9673 g = 197.1 mmol/Kg.

The used ligands and additives are oleic acid (OA, tech. 90 %), diphenylphosphine ($HPPh_2$, 99 %, Strem Chemicals). oleic amine (OLA, $C_{18}H_{35}NH_2$, tech. 70 %), 1-dodecanethiol ($C_{12}H_{25}SH$, 98 %), and lauryl alcohol ($C_{12}H_{25}OH$, 98 %).

The elemental chalcogens used are sulfur (S, precipitated, Anachemia), selenium (Se, 200 mesh, 99.999 %, Alfa Aeser), and tellurium (Te, 200 mesh, 99.8 %). n-trioctylphosphine (TOP, tech. 90 %) was used to prepare SeTOP and TeTOP. Se=PPh_2H and S=PPh_2H were prepared following published procedures[2,15,29,31,37,38]. Compound **3** was made in house[2,38].

For the assignment of Compound **1** and **2**, sodium hydride (NaH, 95 %, dry), chlorodiphenylphosphine (Ph_2P–Cl, 97 %, Alfa Aeser) were used.

Solvents used for purification were anhydrous, including toluene (99.8 %), hexane ($\geq$ 99 %), and methanol (99.8 %), along with acetone (99.5 %, ACP in Montreal, dried over 4 Å molecular sieves).

^{31}P NMR measurements. ^{31}P NMR was performed on a Bruker AV-III 400 spectrometer operating at 161.98 MHz, referenced with an external standard, 85 % H_3PO_4. Usually, we used D1=2 seconds (64 scans total taking ~3 mins) (unless mentioned otherwise). NMR samples were usually prepared and loaded in NMR tubes in a glovebox and properly sealed.

Figure 1. [Se] = ~15 mmol/Kg. $HPPh_2$ (0.01 mmol, 20.4 mg of 0.4865 mmol/g solution in ODE) was added to the mixture of $Cd(OA)_2$ (0.02 mmol, 81 mg, 0.246 mmol/g in ODE) and SeTOP (0.01 mmol, 29.4 mg of 0.339 mmol/g of SeTOP in ODE) in 0.5 g of toluene (tol) (a).
(b) with $C_{18}H_{35}NH_2$ (0.02 mmol, 0.1 ml of 0.2M RNH_2 in ODE).
(c) with $C_{12}H_{25}SH$ (0.02 mmol, 0.067ml of 0.3M solution in ODE).
(d) with $C_{12}H_{25}OH$ (0.02 mmol, 32.3 mg of 0.625 mmol/g in ODE).

Figure 2. [S] = ~17 mmol/Kg. S=PPh_2H (0.01 mmol, 32 mg of 0.3136 mmol/g solution in Tol) was added to

(a) $Cd(OA)_2$ (0.02 mmol) in Tol 0.48 g.
(b) the mixture of $Cd(OA)_2$ (0.02 mmol) and RNH_2 (0.04 mmol, 0.2ml of 0.2M RNH_2 in ODE) in 0.32g of tol.
(c) the mixture of $Cd(OA)_2$ (0.02 mmol), and RSH (0.04 mmol, 0.133 ml of 0.3M in ODE) in 0.38 g of tol.
(d) the mixture of $Cd(OA)_2$ (0.02 mmol) and ROH (0.04 mmol, 64 mg of 0.625 mmol/g in ODE) in 0.42 g of tol.

Figure 3. [Te] = ~ 16 mmol/Kg. $HPPh_2$ (0.02 mmol) was added to the mixture of $Cd(OA)_2$ (0.02 mmol), TeTOP (0.01 mmol, 50.6 mg of 0.2 mmol/g TeTOP in ODE) in 0.4g tol (a).

(b) with $C_{18}H_{35}NH_2$ (0.02 mmol).
(c) with $C_{12}H_{25}SH$ (0.02 mmol).
(e) with $C_{12}H_{25}OH$ (0.02 mmol).

Figure 4. [Cu] or [In] = ~20 mmol/Kg.

(a) CuI-$C_{12}H_{25}SH$ solution (0.013 mmol, 0.21 ml of 0.063 mmol/ml of CuI in $C_{12}H_{25}SH$) was added to a toluene solution of Se=PPh_2H (0.028 mmol, 87 mg of 0.3267 mmol/g toluene solution). 0.5 g tol was added.
(b) CuI-$C_{12}H_{25}SH$ solution (0.013 mmol) was added to a toluene solution of Se=PPh_2H (0.026 mmol) mixed with $C_{18}H_{35}NH_2$ (0.105 mmol). 0.4 g tol was added.
(c) Replace the amine with $C_{12}H_{25}OH$ (0.1049 mmol, 167 mg of 0.628 mmol/g in ODE).
(d) $In(OAc)_3$-$C_{12}H_{25}SH$ (Gel like) (0.01 mol, 412 mg of 0.02485 mmol/g) was mixed with Se=PPh_2H (0.021 mmol). 0.3g of tol was added.
(e) Repeat (d), at room temperature for 15 minutes.
(f) $In(OAc)_3$-$C_{12}H_{25}SH$ (Gel like) (0.01 mmol) was mixed with CuI-$C_{12}H_{25}SH$ solution (0.01 mmol). 0.25 g of Toluene was added and mixed well with a pipet. To this mixture, Se=PPh_2H (0.04 mmol, 123 mg of 0.3267 mmol/g) and transferred to NMR tube.

Figure 5. To a mixture of $(Ph_2PSe_2)_2Cd$ (0.005 mmol, 4.2 mg) and $Cd(OA)_2$ (0.03 mmol, 124 mg of 0.2459 mmol/g in ODE) was added

(a) $C_{18}H_{35}NH_2$ (0.08 mmol, 21.7 mg) in tol_d8 (0.6g) at RT.
(b) $C_{12}H_{25}SH$ (0.02 mmol, 4.5 mg) in tol (0.5g) at RT.
(c) $C_{12}H_{25}OH$ (0.08 mmol, 15.0 mg) in tol (0.5g) at RT.

Computation: our DFT calculations were performed using Gaussian 09[40], with ethyl groups (–C_2H_5) applied to represent the alkyl group of $C_{17}H_{33}$COO–, $C_{18}H_{35}$NH–, $C_{12}H_{25}$S–, and $C_{12}H_{25}$O–; no simplicity was applied for the phenyl group of –PPh_2[38]. Full geometry optimizations were carried out to locate all of the stationary points, via a hybrid B3LYP functional method[41] with the SDD basis set[42,43] and the corresponding effective core potential (ECP) for the Cd, Se, and Te atoms, and the all-electron 6-31++G(d, p) basis set[44,24] for the other atoms of C, H, O, N, P, and S, namely B3LYP/6-31++G(d, p), SDD. The use of ECP and all-electron basis follows Ref 38.

Systematic harmonic frequency calculations were performed to ensure all the structures obtained are true minima on the potential energy surfaces. A polarized continuum model (PCM-SMD)[20,21] with dielectric constant $\varepsilon = 2.0$ was utilized to simulate the solvent effect of 1-octadecene (ODE), via a hybrid M06 functional method[22] with the same basis sets as mentioned above, by performing single-point calculation on the optimized structures at the B3LYP/6-31++G(d, p), SDD level, namely M06//B3LYP/6-31++G(d, p), SDD. The charges and dominant occupancies of natural bond orbitals have been analyzed with the help of the natural bond orbital (NBO) analysis[45,46].

Unless otherwise mentioned, the Gibbs free energy of formation (ΔG) is relative to the initial reactants including ZPE correction obtained at M06//B3LYP/6-31++G(d, p), SDD level in ODE solution under room temperature and atmospheric pressure (25 °C and 1 atm). We would like to point out that B3LYP was used for full geometry optimization while M06 for the Gibbs free energy (ΔG) (in ODE) of the structure obtained. This is due to the fact that M06 takes too much more time for geometry optimization while provides higher accuracy for the energy calculation[34]. Similar results were obtained from our comparison of B3LYP and M06 for full geometry optimization followed by M06 for energy[47].

Supplementary Discussion

Description of the results in Supplementary Table 3, Table 4, Table 5, Table 6, Table 7, Table 8, Table 9, Table 10, Table 11, Table 12, Table 13, Table 14, Table 15, Table 16 and Table 17.

These tables present all the DFT structures considered in this work. **A** species all have the formula $CdSe_2(PPh_2)_2$ but differ in either connectivity or conformation. **A1a** is *trans* and **A1b** is *syn* with respect to the linear Se-Cd-Se fragment with the *trans* isomer more stable by 4.6 kJ mol^{-1} and both are the most stable constitutional isomers. **A2** is the next most stable with a 4-membered ring of Cd*-Se-P-Se-(Cd*). The (*) indicates that it is the same Cd. **A3** has Se-P-Cd connectivity which is different from **A1a** with P-Se-Cd connectivity suggesting that Se–Cd bonds are better than P–Cd bonds in these species. **A4** is the ring opened isomer of **A2** and is 32.9 kJ mol^{-1} less stable. In all cases this magnitude of difference is found for ring opening of analogous 4-membered rings in all calculated structures in this work. **A5** contains a Se–Se bond whereas **A6** and **A7** are two isomers with P–P bonds. All three isomers are high energy and are unlikely to be important in the mechanism.

All **B-Y(NHR)** have the formula $RHNCdSePPh_2$. **B1a-Y(NHR)** is the lowest energy as described in the main text as **B1** (Fig. 7) and has the Cd-P-N connectivity whereas **B1b-Y(NHR)** has the connectivity Cd-N-P which is 15.2 kJ mol^{-1} less stable. **B2a-Y(NHR)** as described in the main text as **B2** (Fig. 7) has a direct Cd–N terminal bond which is 10.1 kJ mol^{-1} less stable than **B1a-Y(NHR)** whereas **B2b-Y(NHR)** is a rotamer about a P–Se bond with the P lone pair pointing towards Cd and the attached phenyl groups away. The contra intuitive isomer with the P lone pair pointing away from Cd and the phenyls towards Cd is the lowest energy by 4.1 kJ mol^{-1}. This rotamer is the lowest energy in all calculated species with this conformational possibility. **B3-Y(NHR)** has a Se–N bond that is much higher in energy. In fact no low energy species with Se–Y bonds were found in this study.

All **B-Y(SR)** have the formula $RSCdSePPh_2$. As for **B-Y(NHR)**, **B1a-Y(SR)** has the connectivity Cd-P-S and **B1b-Y(SR)** has the connectivity Cd-S-P but both are much less stable than **B2a-Y(SR)** or its slightly less stable rotamer **B2b-Y(SR)**. Similarly **B3-Y(SR)** has an unfavorable Se–S bond.

All **B-Y(OR)** have the formula $ROCdSePPh_2$. As for **B-Y(NHR)**, **B1a-Y(OR)** has the connectivity Cd-P-O and **B1b-Y(OR)** has the connectivity Cd-O-P. However, comparing both with **B2a-Y(OR)** or its slightly less stable rotamer **B2b-Y(OR)** they are only slightly less stable. This difference is close to the accuracy of the DFT method. As well, **B3-Y(OR)** has an unfavorable Se–O bond and is much higher in energy.

All **B-Y(OOCR)** have the formula $RCOOCdSePPh_2$. As for **B-Y(SR)**, **B2a-Y(OOCR)** the directly bound to Cd isomer, that additionally has a Cd*-O-C-O-(Cd*) 4-membered ring, is the most stable. Along with its slightly less stable rotamer **B2b-Y(OOCR)**, both are > 100 kJ mol^{-1} more stable than then the **B1a-Y(OOCR)** with the connectivity Cd-P-OOCR or **B1b-Y(OOCR)** that has the connectivity Cd-O(CR)O-P. **B3-Y(OOCR)** has an unfavorable Se–O bond as well as a 5-membered ring but is much less stable. **B4-Y(OOCR)** is a close isomer of the **B2-Y(OOCR)** rotamers but with Cd-P-Se connectivity and not the more stable Cd-Se-P connectivity of the **B2-Y(OOCR)** rotamers.

The **B-Y(PPh_2)** all have the $CdSe(PPh_2)_2$ formula but were not considered in detail as we believe the most important $Y(PPh_2)$ species are the **A** species. As for **B2a-Y(SR)**, **B2a-Y(PPh_2)** the directly P bound to Cd isomer is the most stable. Its rotamer **B2b-Y(PPh_2)** is less stable as for all similar conformers and both are about 50 kJ mol^{-1} more stable than the **B1-Y(PPh_2)** isomer with the Cd-P-P connectivity. Thus, **B2-Y(PPh_2)** is about 50 kJ mol^{-1} more stable than the rotamers of **B1-Y(PPh_2)**.

All **C-Y(NHR)** have the formula $RHNCdSe_2PPh_2$. **C1-Y(NHR)** is the lowest energy species labelled as **C1** in Fig. 7 in the main text. This isomer has the Cd-Se-P-N connectivity that is as compared to **B1-Y(NHR)** the additional Se is inserted between the Cd and the P. The **C2-Y(NHR)**-isomer analogous to **B2-Y(NHR)** features a 4-membered ring containing both seleniums, Cd*-Se-P-Se-(Cd*), and is 18.7 kJ mol^{-1} less stable than **C1-Y(NHR)**. Two rotamers **C3a-Y(NHR)** and **C3b-Y(NHR)** of the isomers with unfavorable Se–N bonds were found. **C4-Y(NHR)** is the ring opened isomer of **C2-Y(NHR)** but it is 29.5 kJ mol^{-1} less stable. This energy difference for 4-membered ring versus ring opened is slightly lower than most other cases calculated in this work perhaps reflecting the stability of **C1-Y(NHR)** over **C2-Y(NHR)**. **C5-Y(NHR)** is **C1-Y(NHR)** with the alternate Cd-P-Se connectivity, **C6-Y(NHR)** has a Se–Se bond and **C7-Y(NHR)** is a **C2-Y(NHR)** type isomer but with the Se inserted between Cd and N. All three of these isomers are high energy and not likely relevant to the reaction mechanism.

The same eight isomers (**C1-Y(SR)** – **C7-Y(SR)**) were calculated as for **Y(NHR)** the overall trends for **C3-Y(SR)** to **C7-Y(SR)** are the same but the **C1-Y(NHR)** over **C2-Y(NHR)** stability is dramatically reversed compared to **C2-Y(SR)** being 125.1 kJ mol^{-1} more stable than **C1-Y(SR)**. Also, the 4-membered ring opened isomer **C4-Y(SR)** is 47.8 kJ mol^{-1} less stable than **C2-Y(SR)** further supporting a strong stability for this Cd*-Se-P-(Cd*) bonding arrangement in the presence of a Cd*–S bond.

The same eight isomers (**C1-Y(OR)** – **C7-Y(OR)**) were calculated as for **Y(NHR)** and **Y(SR)**, the overall trends for **C3-Y(OR)** to **C7-Y(OR)** are the same but the **C1-Y(NHR)** over **C2-Y(NHR)** stability and the **C2-Y(SR)** over **C1-Y(SR)** are levelled out such that **C2-Y(OR)** is only 2.3 kJ mol^{-1} more stable than **C1-Y(OR)**. This difference is within the absolute error bars of the DFT method such that these two isomers can be considered isoenergetic. Also, the 4-membered ring opened isomer **C4-Y(OR)** is 36.9 kJ mol^{-1} less stable than **C2-Y(OR)** which is about halfway between the ring opening difference for **Y(NHR)** and **Y(SR)**. We mention this value since such ring opening could be mechanistically important for **E2** to **E1** or **F2** to **F1** interconversions.

The same eight isomers (**C1-Y(OOCR)** – **C7-Y(OOCR)**) were calculated as for **Y(NHR)**, **Y(SR)** and **Y(OH)**, the overall trends for **C3-Y(OOCR)** to **C7-Y(OOCR)** are the same. As well, **Y(OOCR)** is more like **Y(SR)** than **(NHR)** such that **C2-Y(OOCR)** is 128.5 kJ mol^{-1} more stable than **C1-Y(OOCR)**. Due to the possibility of the two oxygens of the carboxylate binding an additional isomer **C3c-Y(OOCR)** was calculated but none of these isomers are likely to contribute to the mechanism based on energetic stability. The 4-membered ring opened isomer **C4-Y(OOCR)** is the second most stable isomer but is 39.1 kJ mol^{-1} less stable than **C2-Y(OOCR)** and except as a possible short lived intermediate or TS is not likely to be mechanistically important.

For the dimeric (Cd_2) species **E** and **F**, less isomers were calculated but the bonding trends were found to be similar to the **C** and **B** species except for some important details to be highlighted in the following descriptions. It should be recalled that **E1** and **F1** species are the proposed immediate precursors to **1** and **2**, respectively. All **E-Y(NHR)** have the formula $(RHNCdSePPh_2)_2$. In this respect **E1a-Y(NHR)** is significantly more stable than **E2-Y(NHR)** by 153 kJ mol^{-1}. Thus, **E2-Y(NHR)** is unlikely to be mechanistically important. **E1a-Y(NHR)** has pre-formed **1c** as part of its structure attached to Cd. **E1b-Y(NHR)** has the alternate Se-Cd-N-P connectivity from that of **E1a-Y(NHR)** with the Se-Cd-P-N connectivity. However, it is effectively isoenergetic to **E1a-Y(NHR)**. The relevance of this observation is not known.

All **E-Y(SR)** have the formula $(RSCdSePPh_2)_2$ and as for **B2-Y(SR)** being more stable than **B1-Y(SR)**, the dimer **E2-Y(SR)** is more stable than **E1a-Y(SR)**. But, the energy difference of 32.7 kJ mol^{-1} is

significantly less than the 82.5 kJ mol^{-1} difference for **B2a-Y(SR)** to **B1-Y(SR)**. This observation supports the proposed ligand loss mechanism through **E1** like dimeric species that is dimerization appears to favor isomers that lead to formation of species that have pre-formed **1**, **1d** in this case. Also, the **E1b-Y(SR)** isomer with the alternate Se-Cd-S-P connectivity from that of **E1a-Y(SR)** with the Se-Cd-P-S connectivity is less stable than **E1a-Y(SR)**.

All **E-Y(OR)** have the formula $(ROCdSePPh_2)_2$. The shift after dimerization towards species with preformed **1** (**1e** in this case) is also found for **E1a-Y(OR)** which is calculated to be significantly more stable than **E2-Y(OR)** by 127.8 kJ mol^{-1} whereas **B1-Y(OR)** was 6.9 kJ mol^{-1} less stable than **B2-Y(OR)**. In this case the isomer **E1b-Y(OR)** with alternate connectivity was much less stable than **E1a-Y(OR)**.

All **E-Y(OOCR)** have the formula $(RCOOCdSePPh_2)_2$. The trend for preformed **1** after dimerization is not found for **Y(OOCR)** as **E2-Y(OOCR)** is significantly more stable than **E1a-Y(OOCR)** by 107.3 kJ mol^{-1}. Isomers **E1b-Y(OOCR)** and **E1c-Y(OOCR)** with different connectivities are even less stable than **E1a-Y(OOCR)** and are unlikely to be mechanistically important.

The **F-Y(NHR)** have the formula $(RHNCdSe_2PPh_2)_2$. For these dimeric species the trend to in this case preformed **2** is also found. Notably, **F1-Y(NHR)** with preformed **2c** is 196.6 kJ mol^{-1} more stable than **F2-Y(NHR)**. **F3a-Y(NHR)** and **F3b-Y(NHR)** are isomers with generally unfavorable Se–N bonds. Calculated isomers **F4-Y(NHR)** and **F6-Y(NHR)** have different connectivities but neither are favorable. **F5-Y(NHR)** is the twice ring opened by breaking of two P–Se bonds isomer of **F2-Y(NHR)**. **F5-Y(NHR)** also has pre-formed **2c** but connected to Cd by a Cd–N bond rather than a Cd–Se bond. It is also significantly less stable than **F1-Y(NHR)** and except as a possible intermediate or TS not likely mechanistically important.

The **F-Y(SR)** all have the formula $(RSCdSe_2PPh_2)_2$. For these **Y(SR)** species the **F2-Y(SR)** isomer that results from direct dimerization of **C2-Y(SR)** are more stable than **F1-Y(SR)** by 78 kJ mol^{-1}. This compares to the **C2-Y(SR)** versus **C1-Y(SR)** energy difference of 125.1 kJ mol^{-1}. The isomer **C3-Y(SR)** with alternate connectivity, notably Se–P bonds, is also much less stable than **F2-Y(SR)**.

The **F-Y(OR)** all have the formula $(ROCdSe_2PPh_2)_2$. For these **Y(OR)** species the **F1-Y(OR)** isomer that results from direct dimerization of **C1-Y(OR)** and have pre-formed **2e** are more stable than **F2-Y(OR)** by 126.1 kJ mol^{-1}. This is very different from the very small energy difference of 2.1 kJ mol^{-1} for **C2-Y(OR)** versus **C1-Y(OR)**. This result also supports that dimerization favors isomers with preformed **2**. The isomer **C3-Y(OR)** with alternate connectivity, notably Se–P bonds, is also much less stable than **F1-Y(OR)**.

The **F-Y(OOCR)** all have the formula $(OOCRCdSe_2PPh_2)_2$. For these **Y(OOCR)** species the **F2-Y(OOCR)** isomer that results from direct dimerization of **C2-Y(OOCR)** are more stable than **F1-Y(OOCR)** by 78.8 kJ mol^{-1}. This is smaller than the 128.5 kJ mol^{-1} for **C2-Y(OOCR)** over **C1-Y(OOCR)** but still suggests that **F2-Y(OOCR)** would be formed. The isomer **C3-Y(OOCR)** with alternate connectivity, notably Se–P bonds, is also much less stable than **F2-Y(OOCR)**.

The **D** species are equivalent to **F-Y(PPh$_2$)** with the formula $(Cd(Se_2PPh_2)_2)_2$. The **D2** isomer is the direct dimer of proposed initial intermediate **A** and could be the immediate precursor of all other **E** and **F** dimers after reaction with the various YH and noting the equilibria with $E{=}PPh_2H$ and PPh_2H discussed in the main text. **D2** is calculated to be the most stable dimer among those calculated and is 35.5 kJ mol^{-1} more stable than **D3**. **D2** is even more stable than **D1** which is the direct dimer of **A1a**.

Supplementary References

1. Shin, J., Bertoia, J., Czerwinski, K. R. & Bae, C. A new homogeneous polymer support based on syndiotactic polystyrene and its application in palladium-catalyzed Suzuki–Miyaura cross-coupling reactions. *Green Chem*. **11**, 1576–1580 (2009).
2. Yu, K., Liu, X., Zeng, Q., Yang, M. Ouyang, J., Wang, X. &Tao Y. The formation mechanism of binary semiconductor nanomaterials shared by single-source and dual-source precursor approaches at ambient temperature. *Angew. Chem. Int. Ed.* **52**, 11034–11039 (2013).
3. Hendricks, M. P. *et al*. The importance of nanocrystal precursor conversion kinetics: mechanism of the reaction between cadmium carboxylate and cadmium bis(diphenyldithiophosphinate). *ACS Nano* ***6***, 10054–10062 (2012).
4. McCleverty, J. A. *et al*. Aspects of the Inorganic Chemistry of Rubber Vulcanisation. Part 4. Dialkyl- and diaryl-dithiophosphate and -dithiophosphinate complexes of zinc: phosphorus-31 nuclear magnetic resonance spectral studies and structures of $[NMe_4][Zn\{S_2P(OC_6H_4Me\text{-}p)_2\}_3]$ and $[NEt_4][Zn(S_2PPh_2)_3]$. *J. Chem. Soc. Dalton Trans*. 627–635 (1983).
5. Goda, K. *et al*. Reactions of alkyl diphenylphosphinates and related thio esters with some nucleophiles. S_N2 (S) reaction and Wittig type rearrangement. *Bulletin of the Chemical Society of Japan* **51**, 260–264 (1978)
6. Al-Shboul, T. M. A. *et al*. Oxidation products of calcium and strontium bis(diphenylphosphanide). *Inorg. Chem*. **51**, 7903–7912 (2012).
7. Kriz, J. et al. Interaction of hydrated protons with trioctylphosphine oxide: NMR and theoretical study. *J. Phys. Chem. A* **113**, 5896–5905 (2009).
8. Hilliard, C. R., Bhuvanesh, N., Gladysz, J. A. & Blumel, J. Synthesis, purification, and characterization of phosphine oxides and hydrogen peroxide adducts. *Dalton Trans.* **41**, 1742–1754 (2012).
9. Artem'ev. A. V. *et al*. Efficient general synthesisof alkylammonium diselenophosphinates via multicomponent one-pot reaction of secondary phosphines with elemental selenium and amines. *Synthesis* **21**, 3724–3730 (2010).
10. Davies, R. P. *et al.* Coordination chemistry of diselenophosphinate complexes: the X-ray single-crystal structures of $[K(Se_2PPh_2)(THF)_2]_2$ and $[In(Se_2PPh_2)_3]{\bullet}L$ (L = THF, PhMe). *Inorg. Chem.* **43**, 4802–4804 (2004).
11. Nguyen, L. M. *et al*. Convenient synthesis of copper 9I) thiolates and related compounds. *Inorg. Chim. Acta*. **358**, 1331–1336 (2005).
12. Dance, I. G., Guerney, P. J., Rae, A. D. & Scudder, M. L. Planar bridging thiolate in $(Ph_3P)_2Cu(\mu\text{-}SPh)_2Cu(PPh_3)_2$. *Inorg. Chem.* **22**, 2883–2887 (1983).
13. Lemmen, T. H. *et al.* Alcohol elimination chemistry of tetrakis(tert-butoxocopper). *Inorg. Chem.* **29**, 3680–3685 (1990).
14. Cain, M. F. *et al* Synthesis and structure of intermediates in copper-catalyzed alkylation of diphenylphosphine. *Inorg. Chem.* **49**, 7650–7662 (2010).
15. Yu, K., *et al*. Low-temperature approach to highly emissive copper indium sulfide colloidal nanocrystals and their bioimaging applications. *ACS Appl. Mater. Interfaces* **5**, 2870–2880 (2013).
16. Lee, A. M., Handy, N. C. & Colwell, S. M. The density functional calculation of nuclear shielding constants using London atomic orbitals. *J. Chem. Phys.* **103**, 10095–10109 (1995).

17. Schreckenbach, G. & Ziegler, T. Calculation of NMR shielding tensors using gauge-including atomic orbitals and modern density functional theory. *J. Chem. Phys.* **99**, 606–611 (1995).
18. Rauhut, G., Puyear, S., Wolinski, K. & Pulay, P. Comparison of NMR shieldings calculated from Hartree-Fock and density functional wave functions using gauge-including atomic orbitals. *J. Chem. Phys.* **100**, 6310–6316 (1996).
19. Cheesemann, J. R., Trucks, G. W., Keith, T. A. & Frisch, M. J. A comparison of models for calculating nuclear magnetic resonance shielding tensors. *J. Chem. Phys.* **104**, 5497–5509 (1996).
20. Cammi, R., Mennucci, B. & Tomasi, J. Fast evaluation of geometries and properties of excited molecules in solution: a tamm-dancoff model with application to 4-dimethylaminobenzonitrile. *J. Chem. Phys. A* **104**, 5631–5637 (2000).
21. Marenich, A. V., Cramer, C. J. & Truhlar, D. G. Uniform treatment of solute−solvent dispersion in the ground and excited electronic states of the solute based on a solvation model with state-specific polarizability. *J. Chem. Theory Comput*. **9**, 609–620 (2013).
22. Zhao, Y. & Truhlar, D. G. The M06 suite of density functionals for main group thermochemistry, thermochemical kinetics, noncovalent interactions, excited states, and transition elements: two new functionals and systematic testing of four M06-class functionals and 12 other functionals. *Theor. Chem. Acc.* **120**, 215–241 (2008).
23. Krishnan, R., Binkley, J. S., Seeger, R. & Pople, J. A. Self-consistent molecular orbital methods. XX. a basis set for correlated wave functions. *J. Chem. Phys.* **72**, 650–654 (1980).
24. McLean, A. D. & Chandler, G. S. Contracted gaussian basis sets for molecular calculations. I. second row atoms, Z=11-18. *J. Chem. Phys.* **72**, 5639–5648 (1980).
25. Chivers, T. & Laitinen, R. S. Tellurium: a maverick among the chalcogens. *Chem. Soc. Res*. ***44***, 1725–1730 (2015).
26. Evans, C. M., Evans, M. E. & Krauss, T. D. Mysteries of TOPSe revealed: insights into quantum dot nucleation. *J. Am. Chem. Soc.* **132**, 10973–10975 (2010).
27. Cossairt, B. M. & Owen, J. S. At the interface of small molecules and quantum dots. *Chem. Mater*. **23**, 3114–3119 (2011).
28. Garcia-Rodriguez, R. & Liu, H. Mechanism study of the synthesis of CdSe nanocrystals: release of selenium. *J. Am. Chem. Soc*. **134**, 1400–1403 (2012).
29. Yu, K., Liu, X., Zeng, Q., Leek, D. M., Ouyang, J., Whitmore, K. M., Ripmeester, J. A., Tao, Y. & Yang, M. Effect of tertiary and secondary phosphines on low-temperature formation of quantum dots. *Angew. Chem. Int. Ed*. **52**, 4823–4828 (2013).
30. Yu, K., Liu, X., Chen, Q. Y., Yang, H., Yang, M., Wang, X., Cao, H., Whitfield, D. M., Hu, C. & Tao, Y. Mechanistic study of the role of primary amines in precursor conversions to semiconductor nanocrystals at low temperature. *Angew. Chem. Int. Ed.* **53**, 6898–6904 (2014).
31. Yu, K., Hrdina, A., Ouyang, J., Kingston, D., Wu, X., Leek, D. M., Liu, X. & Li, C. Ultraviolet $ZnSe_{1-x}S_x$ gradiently-alloyed nanocrystals via a noninjection approach. *ACS Appl. Mater. Interfaces* **4**, 4302–4311 (2012).
32. Zhang, J., Yang, Q., Cao, H., Ratcliffe, C. I., Kingston, D., Chen, Q. Y., Ouyang, J., Wu, X., Leek, D. M., Riehle, F. S. & Yu, K. "Bright Gradient-Alloyed $CdSe_xS_{1-x}$ Quantum Dots Exhibiting Cyan-Blue Emission". *Chem. Mater*. **28***,* 618–625 (2016).
33. Liu, H., Owen, J. S. & Alivisatos, A. P. Mechanistic study of precursor evolution in colloidal group II-VI semiconductor nanocrystal synthesis. *J. Am. Chem. Soc.* **129**, 305–312 (2007).
34. Zhao, Y. & Truhlar, D. G. Density functionals with broad applicability in chemistry. *Accounts Chem. Res*. **41**, 157–167 (2008).

35. Ouyang, J., Schuurmans, C., Zhang, Y., Nagelkerke, R., Wu, X., Kingston, D., Wang, Z. Y., Wilkinson, D., Li, C., Leek, D. M., Tao, Y. & Yu, K. Low-temperature approach to high-yield and reproducible syntheses of high-quality small-sized PbSe colloidal nanocrystals for photovoltaic applications. *ACS Appl. Mater. Interfaces* **3**, 553–565 (2011).
36. Yu, K., Ouyang, J. & Leek, D. M. *In-situ* observation of nucleation and growth of PbSe magic-sized nanoclusters and regular nanocrystals. *Small* **7**, 2250−2262 (2011).
37. Yu, K., Hrdina, A., Zhang, X., Ouyang, J., Leek, D. M., Wu, X., Gong, M., Wilkinson, D. & Li, C. Highly-photoluminescent ZnSe nanocrystals *via* a non-injection-based approach with precursor reactivity elevated by a secondary phosphine. *Chem. Commun.* **47**, 8811–8813 (2011).
38. Yu, K. *et al*. Mechanistic study of the role of primary amines in precursor conversions to semiconductor nanocrystals at low temperature. *Angew. Chem. Int. Ed.* **53**, 6896–6904 (2014).
39. Yu, K., Zaman, B., Romanova, S., Wang, D. S. & Ripmeester, J. A. Sequential synthesis of type II colloidal CdTe/CdSe core-shell nanocrystals. *Small* **1**, 332–338 (2005).
40. Frisch, M. J., *et al*. *Gaussian 09, Revision C.01;* Gaussian, Inc., Wallingford, CT, **2010**.
41. Becke, A. D. Density-functional thermochemistry. III. the role of exact exchange. *J. Chem. Phys.* **98**, 5648–5652 (1993).
42. Andrae, D., Häuβermann, U., Dolg, M., Stoll, H. & Preuβ, H. Energy-adjusted ab initio pseudopotentials for the second and third row transition elements. *Theor. Chim. Acta***. 77**, 123–141 (1990).
43. Igel-Mann, G., Stoll, H. & Preuss, H. Pseudopotentials for main group elements (IIIa through VIIa). *Mol. Phys.* **65**, 1321–1328 (1988).
44. Krishnan, R., Binkley, J. S., Seeger, R. & Pople, J. A. Self-consistent molecular orbital methods. XX. a basis set for correlated wave functions. *J. Chem. Phys.* **72**, 650–654 (1980).
45. Reed, A. E., Weinstock, R. B. & Weinhold, F. Natural population analysis. *J. Chem. Phys.* **83**, 735–746 (1985).
46. Reed, A. E., Curtiss, L. A. & Weinhold, F. Intermolecular interactions from a natural bond orbital, donor-acceptor viewpoint. *Chem. Rev.* **88**, 899–926 (1988).
47. Qi, T.,Yang, H., Whitfield, D. M., Yu, K. & Hu, C. Insights into the mechanistic role of diphenylphosphine selenide, diphenylphosphine, and primary amines in the formation of CdSe monomers. *J. Phys. Chem. A* **120**, 918–931 (2016).
